# p‐Cymene Targets Multiple Oncogenic Pathways in Hepatocellular Carcinoma: Insights From Network Pharmacology and In Vitro Studies

**DOI:** 10.1002/fsn3.71108

**Published:** 2025-10-16

**Authors:** Nadia Anwar, Muhammad Nasir Hayat Malik, Muhammad Atif, Abdullah R. Alanzi, Hattan A. Alharbi, Waqas Younis, Munawar Abbas, Gideon F. B. Solre

**Affiliations:** ^1^ Faculty of Pharmacy The University of Lahore Lahore Pakistan; ^2^ Department of Pharmacognosy, College of Pharmacy King Saud University Riyadh Saudi Arabia; ^3^ College of Food Science and Engineering Henan University of Technology Zhengzhou China; ^4^ Department of Chemistry, Thomas J. R. Faulkner College of Science and Technology University of Liberia Monrovia Montserrado Liberia

**Keywords:** anti‐cancer, BCL2, CASP3, HCC, P53, p‐cymene, VEGF

## Abstract

This study explored the anticancer potential of p‐cymene against hepatocellular carcinoma (HCC) through computational and in vitro approaches. Bioinformatics analysis identified 635 potential targets of p‐cymene, with 216 overlapping HCC‐related proteins. Target interaction networks were constructed using STRING and Cytoscape, revealing key proteins involved in apoptosis, angiogenesis, and tumor progression. Molecular docking was performed using the molecular operating environment software, demonstrating strong binding affinities of p‐cymene with key overlapping HCC targets, including hypoxia‐inducible factor 1‐alpha (HIF1A), B‐cell lymphoma 2 (BCL2), cyclin‐dependent kinase 9 (CDK9), Janus kinase 2 (JAK2), vascular endothelial growth factor (VEGF), mitogen‐activated protein kinase 4 (MAPK4), tumor protein p53 (P53), signal transducer and activator of transcription 3 (STAT3), and caspase‐3 (CASP3). HepG2 cells were treated with increasing concentrations of p‐cymene (5–50 mM), and cytotoxicity was assessed using MTT, crystal violet, and trypan blue exclusion assays. Antioxidant activity was measured by evaluating superoxide dismutase (SOD) and glutathione (GSH) levels. Apoptotic markers, including CASP3, P53, VEGF, and BCL2, were quantified using ELISA. Results showed a dose‐dependent reduction in HepG2 cell viability, with significant cytotoxic effects at higher p‐cymene concentrations (30 and 50 mM). p‐Cymene reduced oxidative stress, evident from increased SOD and GSH levels, and triggered apoptosis, as indicated by increased CASP3 and P53 expression. Additionally, BCL2 and VEGF were downregulated, suggesting inhibition of cell survival and angiogenesis. These findings highlight p‐cymene's multi‐targeted anticancer effects in HCC cells, supporting its further evaluation in in vivo models and potential combination therapies for improved therapeutic outcomes.

## Graphical abstract

p‐Cymene exhibited a strong affinity for key hepatocellular carcinoma (HCC) targets and induced dose‐dependent cytotoxicity in HepG2 cells while enhancing antioxidant defense mechanisms. The treatment significantly modulated apoptotic and angiogenic markers, notably increasing Caspase‐3 and p53, and suppressing BCL2 and VEGF expression. Collectively, these findings position p‐cymene as a compelling candidate for multi‐targeted HCC therapy.

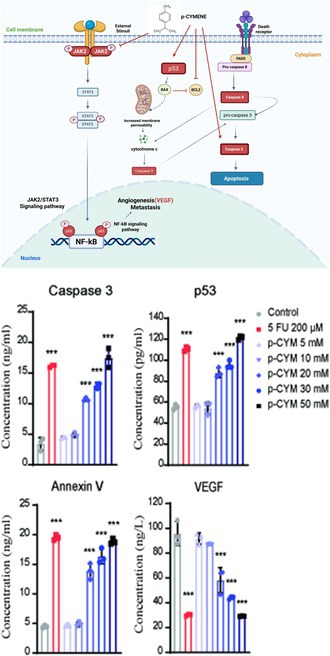

## 1 Introduction

Hepatocellular carcinoma (HCC) is the most common type of primary liver cancer and represents a significant global health challenge due to its high mortality rate and poor prognosis (Singal et al. 2023). It ranks as the sixth most prevalent cancer worldwide and is the third leading cause of cancer‐related deaths (Toh et al. 2023). According to recent estimates, nearly 800,000 new cases of HCC are diagnosed annually, with the highest prevalence observed in regions such as East Asia and sub‐Saharan Africa, where chronic hepatitis B and C infections are endemic (Carr 2021; Suresh et al. 2020). The burden of HCC continues to rise due to a complex interplay of risk factors, including cirrhosis, chronic alcohol consumption, non‐alcoholic fatty liver disease (NAFLD), and exposure to environmental carcinogens such as aflatoxins. The increasing incidence of metabolic disorders, particularly obesity and diabetes, further exacerbates the risk of HCC, making it a critical public health concern that requires urgent attention (Melaram 2021; Wallace et al. 2015).

Despite advancements in diagnostic techniques, HCC is often diagnosed at an advanced stage when curative treatment options become limited. Surgical resection and liver transplantation remain the gold standards for early‐stage HCC, offering the potential for long‐term survival (Addissouky et al. 2024). However, only a small proportion of patients are eligible for these interventions due to late‐stage diagnosis and underlying liver dysfunction. For patients with intermediate or advanced disease, treatment options primarily include locoregional and systemic therapies targeting molecular pathways involved in tumor progression (Koulouris et al. 2021). The introduction of tyrosine kinase inhibitors, such as Sorafenib and Lenvatinib, has marked a significant step in the management of advanced HCC by inhibiting angiogenesis and tumor growth (Pinto et al. 2023). Nevertheless, these treatments are associated with limited efficacy and significant adverse effects, including hypertension, hand‐foot syndrome, and gastrointestinal toxicity, which severely impact patients' quality of life (Li et al. 2023). These limitations highlight the urgent need for novel, more effective, and safer therapeutic strategies to improve clinical outcomes for HCC patients.

In recent years, there has been growing interest in exploring natural compounds as potential anticancer agents, particularly those derived from plants (Dehelean et al. 2021). Among these, p‐cymene, a naturally occurring monoterpene found in essential oils of various medicinal plants such as thyme, oregano, and cumin, has gained attention for its diverse pharmacological properties (Baginska et al. 2023). Studies have demonstrated that p‐cymene exhibits significant anti‐inflammatory, antioxidant, and anticancer activities, making it a promising candidate for cancer therapy (Singh and Barman 2021). Preliminary research suggests that p‐cymene can induce apoptosis in cancer cells, modulate key signaling pathways involved in tumor progression, and enhance the efficacy of conventional chemotherapy. Additionally, its relatively low toxicity compared to synthetic chemotherapeutic agents presents a potential advantage in reducing treatment‐related side effects (Acikgul et al. 2024; Baginska et al. 2023; Balahbib et al. 2021).

Given these considerations, this paper aims to provide a comprehensive analysis of the current landscape of HCC, emphasizing its existing therapeutic challenges and the emerging role of p‐cymene in cancer treatment. By examining the molecular mechanisms through which p‐cymene exerts its anticancer effects, this study seeks to contribute to the ongoing efforts in identifying novel treatment strategies for HCC. Understanding the therapeutic potential of p‐cymene could pave the way for future preclinical and clinical investigations, ultimately advancing the development of safer and more effective alternatives for HCC management.

## 2 Materials and Methods

### 2.1 Chemical Structure and Potential Targets of p‐Cymene

The chemical structure of p‐cymene was acquired from the PubChem database (https://pubchem.ncbi.nlm.nih.gov/) and downloaded in “SDF” format (Oriola 2024). The “SDF” file was subsequently submitted to the Swiss Target Prediction, WAY2DRUG, SuperPred, and Pharma Mapper databases to forecast the targets of p‐cymene, with a minimum probability (Kar et al. 2022). The relevant targets were added to the DrugBank database (https://go.drugbank.com/). Ultimately, the UniProt database (https://www.uniprot.org/) was employed to standardize the names of the targets following the elimination of duplication (UniProt Consortium 2023).

### 2.2 Identification of HCC Targets and Common Targets Between p‐Cymene and HCC

Prediction of disease‐related genes was the next step to uncover the molecular mechanism of p‐cymene. Three databases, GeneCards, DisGeNET, and TTD, were searched using keywords “Hepatocellular Carcinoma” to retrieve disease‐related genes (Wang et al. 2024). DisGeNET is a multipurpose data system that provides information related to genes, disorders, and their related empirical studies (Chang et al. 2024). The GeneCards database contains information related to the genome, proteome, and transcriptomes of an organism (Orlov and Anashkina 2023). The Venny 2.0 online tool was used to identify the overlap genes between predicted target genes of p‐cymene and HCC (Jiang, Huang, et al. 2024).

### 2.3 Constructing Protein–Protein Interaction Network and Screening Core Targets

The intersection targets were uploaded to the STRING database (https://string‐db.org/), the species was set as “ *Homo sapiens* ,” the minimum required interaction score was 0.4, and the disconnected nodes in the network were hidden (Singh et al. 2024). The node information was downloaded in the format of “TSV” and imported into Cytoscape 3.8.2 software to construct a protein–protein interaction (PPI) network (Chen and Wu 2024). The core targets of p‐cymene against HCC were obtained by using the cytoHubba plug‐in, which was used for molecular docking.

### 2.4 Gene Ontology and Kyoto Encyclopedia of Genes and Genomes Enrichment Analysis

Gene Ontology (GO) is a biological system used to study the impact of individual genes on organisms at different biological levels. Kyoto Encyclopedia of Genes and Genomes (KEGG) provides evidence of the involvement of individual genes in biological signaling pathways. In this study, GO enrichment analysis was performed using the DAVID Database, which includes biological processes (BP), cellular components (CC), molecular functions (MF), and KEGG pathways (Gene Ontology Consortium 2006; Dennis et al. 2003). We used the SRplot platform to visualize our findings in bubble charts and bar chart format, demonstrating its usefulness as an online data analysis tool.

### 2.5 Drug‐Target‐Pathway Network Construction

By importing the intersecting genes and KEGG signaling pathway entries into Cytoscape 3.9.1, a drug‐target‐pathway network was established (Pinero et al. 2021). In the network, nodes represent p‐cymene, intersecting genes, or pathways, while the edges between nodes indicate the involvement of genes in different pathways.

### 2.6 Molecular Docking

#### 2.6.1 Structures of Target Proteins

The study involved various proteins, including hypoxia inducible factor 1 subunit alpha (HIF1A; PDB ID: 1lqb), B‐cell lymphoma‐2 (BCL2; PDB ID: 2XA0), cyclin dependent kinase 9 (CDK9; PDB ID: 3BLR), Janus kinase 2 (JAK2; PDB ID: 3JY9), vascular endothelial growth factor (VEGF; PDB ID: 3V2A), mitogen activated protein kinase 4 (MAPK4; PDB ID: 4ZP5), tumor suppressor gene (P53; PDB ID: 5O1H), signal transducer and activator of transcription 3 STAT (STAT3; PDB ID: 6NJS), and caspase‐3 (CASP3; PDB ID: 1NME) (Saif et al. 2021). The targets were preprocessed, which included the removal of co‐crystallized ligands, water molecules, and other heteroatoms, as well as the addition of missing hydrogen atoms and optimization of side‐chain orientations. The protein structures were later subjected to energy minimization using the AMBER99 force field to relax any steric clashes or structural imperfections (Figure S1) (Saif et al. 2021).

#### 2.6.2 Preparation of Ligands

The 2D structures of the ligands ‘p‐cymene’ and ‘fluorouracil’ (5FU) were obtained from virtual databases in the PubChem repository of the National Center for Biotechnology Information (NCBI) (Table S1). Ligand preparation and molecular docking were performed using the Molecular Operating Environment (MOE) software (version 2019.0102). The 2D structures of the ligand molecules were constructed using the MOE builder tool and subsequently saved in the MOE database. Initial preprocessing of the ligands involved the removal of counterions and salts to ensure the correct protonation state. The ligands subsequently underwent energy minimization using the Merck Molecular Force Field (MMFF‐94x) to achieve their most stable conformations before proceeding to docking simulations (Mohamed et al. 2022).

#### 2.6.3 Prediction of Active Binding Sites

The Computed Atlas of Surface Topography of Proteins (CASTp) was used to predict the active binding sites of different protein structures (Michael et al. 2023). The binding site predicted for different proteins is listed in Table S2.

#### 2.6.4 Docking Analysis

Molecular docking and scoring calculations were performed using MOE (version v2019.0102). For visualizing the docking analysis of ligands against various protein targets, the Discovery Studio molecular graphics system was utilized. Initially, a ligand database was created using MOE and converted into a Microsoft Access database file (MDB) format. The input files were uploaded for docking analysis, which included 50 ligand conformations using MOE's default docking algorithm—Triangle Matcher for the method, and London dG and GBVI/WSA dG for the scoring functions. The most favorable docking results were identified through conformational visualization in Discovery Studio, with 2D poses being examined as well.

### 2.7 Cytotoxic Effects of p‐Cymene in HepG2 Cells

HepG2 cells were sourced from the Cells and Tissue Culture Laboratory of The University of Lahore. To determine the cytotoxic concentrations of p‐cymene, a cell viability assay was conducted. Dilutions of p‐cymene (5, 10, 20, 30, and 50 mM) were made from a 1 M stock solution prepared in 1% DMSO solution. HepG2 cells were cultivated in a 96‐well culture plate at 37°C. After 24 h, the cells were washed with 1× phosphate buffer saline (PBS) and exposed to the concentrations of p‐cymene for an additional 24 h (Bai and Rethnaswamy 2024). Following this incubation, the cells were washed again, and viability was assessed using the 3‐[4,5‐dimethylthiazol‐2‐yl]‐2,5 diphenyl tetrazolium bromide (MTT) and crystal violet assay kits (Cat. No. 11465007001; Sigma Aldrich, USA; Cat. No. 548‐62‐9; Sigma Aldrich, USA), following the manufacturers' guidelines (Malik et al. 2025).

### 2.8 Experimental Groups

The study design included the following experimental groups:

1. Control: Complete Dulbecco's modified Eagle medium (DMEM) medium
2. 5FU (200 μM): 200 μM of 5FU in complete DMEM medium
3. p‐CYM (5 mM): 5 mM of p‐cymene in complete DMEM medium
4. p‐CYM (10 mM): 10 mM of p‐cymene in complete DMEM medium
5. p‐CYM (20 mM): 20 mM of p‐cymene in complete DMEM medium
6. p‐CYM (30 mM): 30 mM of p‐cymene in complete DMEM medium
7. p‐CYM (50 mM): 50 mM of p‐cymene in complete DMEM medium

### 2.9 Cell Death Assessment

The trypan blue exclusion method was employed to distinguish between live and dead cells. The pretreated cells were rinsed three times with 1× PBS and stained with trypan blue (Cat. No. T6146; Sigma Aldrich, USA). Dead cells, which absorbed the blue dye, were counted using a compound microscope (Hossain et al. 2021; Malik et al. 2025).

### 2.10 Quantification of HCC Markers

An ELISA assay was performed to measure levels of caspase 3 (Cat. No. ab285337; Abcam, USA), annexin V (Cat. No. MBS9321844; MyBiosource, USA), the tumor suppressor gene (p53, Cat. No. MBS355295; MyBiosource, USA), and vascular endothelial growth factor (VEGF, Cat. No. ab100662; Abcam, USA) from cell lysates, according to the manufacturer's instructions (Malik et al. 2025).

### 2.11 Statistical Analysis

Data from three biological replicates were expressed as mean ± standard deviation (SD) and analyzed using one‐way ANOVA followed by Dunnett's multiple comparison tests. Dunnett's multiple comparison test was used to compare the mean of the treated group with the mean of the control group. In addition, a *t*‐test was applied to compare the positive control group with the negative control. All statistical analyses were conducted using Graph Pad Prism 8.02 software, with a significance threshold set at *p* < 0.05. Significance levels in the graphs were denoted as: ***p ≤ 0.001, **p ≤ 0.01, and *p ≤ 0.05 (treated groups vs. negative control); ^###^ *p* ≤ 0.001 (positive control vs. negative control).

## 3 Results

### 3.1 Predicted Pharmacological Spectrum of p‐Cymene

The biological spectrum of p‐cymene was assessed using the WAY2DRUG (Pass online) database. Proteins were chosen based on their significant upregulation or downregulation. The terms “Probable activity” (Pa) and “probable inactivity” (Pi) were employed to denote their likely functions. For the interpretation of the biological spectrum, only actions where Pa exceeded Pi were included in the current analysis. The predicted biological spectrum of p‐cymene is provided in Table S3.

### 3.2 Target Proteins of p‐Cymene Associated With HCC

**FIGURE 1:**
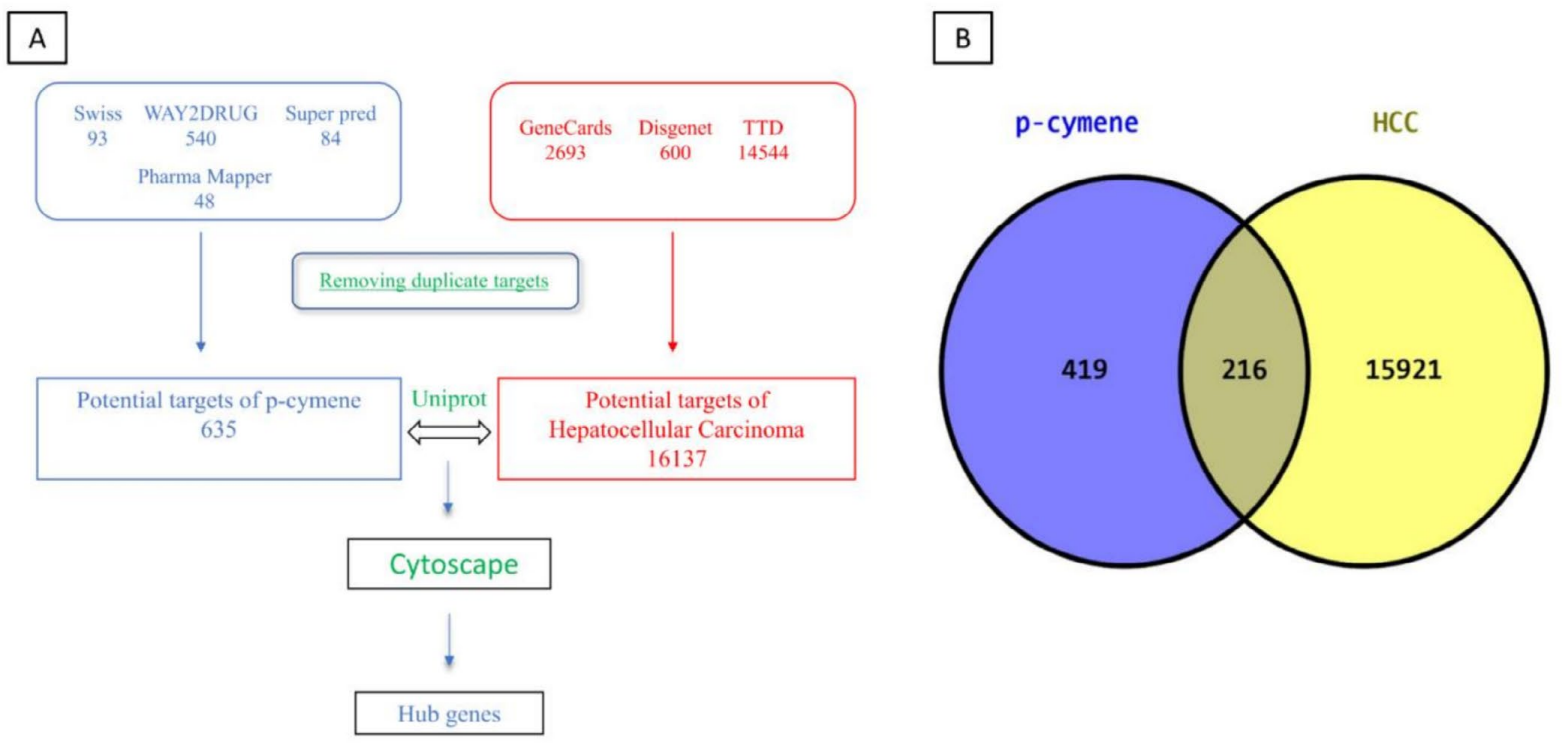
Potential targets of p‐cymene related to HCC. A) It highlights the initial databases used, including Swiss, WAY2DRUG, Super Pred, and others, which yielded 635 potential targets. B) After removing duplicates, 216 targets were found to overlap with HCC‐related protein.

In our investigation of the potential therapeutic targets of p‐cymene in relation to HCC, we employed an integrated approach utilizing various bioinformatics tools and databases. The initial screening identified a total of 635 potential targets for p‐cymene from several sources: SwissTargetPrediction, WAY2DRUG, SuperPred, and Pharma Mapper. Subsequent filtering for duplicate targets resulted in a refined list of 419 unique targets. To establish a comprehensive understanding of HCC‐related targets, we utilized the GeneCards database, which provided a robust dataset comprising 15,921 potential targets associated with HCC. The inclusion of additional databases such as DisGeNET (600 targets) and Therapeutic Target Database (TTD) (1454 targets) further enriched our dataset. The next step involved the integration of these datasets to identify common therapeutic targets. A comparative analysis revealed 216 overlapping targets between the p‐cymene target list and the HCC‐related targets. This intersection suggests that these 216 targets may represent crucial points of interaction for p‐cymene in the context of HCC treatment (Figure 1; Table 1).

**TABLE 1:** Overlapping targets between the p‐cymene target list and the HCC‐related targets.

Total common targets	Common targets between p‐cymene and HCC
216 targets	CYP2A6, ACHE, PPARA, PTGS1, HTR1D, ADORA3, AHR, CYP1A2, SLC6A2, HTR2A, SLC6A4, SLC6A3, KCNN4, KIF11, HTR2B, CA2, GSK3B, AR, MAOA, ESR1, ESR2, CYP2D6, HTR6, CYP2C9, CYP3A4, AOC3, CYP2C19, ESRRG, ADRA2A, CXCL8, PTGS2, SIGMAR1, SLC22A6, CNR2, HRH1, KDM1A, SLC18A2, SRD5A2, TYR, FAAH, CES1, KCNH2, CES2, CNR1, ADRA2B, SHBG, ADRA1A, HTR1B, IDO1, FABP4, FABP3, PLAU, ALOX5, CA4, IGF1R, HSD17B1, HDAC1, CACNA2D1, HTR3A, F2, PNMT, ANPEP, ADRA2C, CYP19A1, CHRM2, CHRM1, CHRM3, ADRB3, S1PR5, S1PR4, ALB, NOS1, S1PR3, S1PR1, SPHK2, MAOB, CYP2J2, MAPK6, CDC25A, MAPK3, PPP5C, CYP2B6, CA3, CYP3A5, DYRK2, TDP1, MTNR1A, CSNK1G1, PDE7B, OXTR, HCRTR1, AGTR2, SORT1, SRD5A1, PLG, PIN1, VEGFA, PDE9A, PDE3A, GLP1R, HMGCR, NPY2R, TNF, MPO, NOS3, P2RY1, PLA2G10, HDAC3, PIK3CG, DHFR, CA13, MGLL, PMM2, CETP, ECE1, SGK2, COMT, ADRA1B, CA5B, PFKFB3, KMO, QPCT, LIPE, MMP14, MBTD1, LDHB, GSTA1, HDAC11, CENPE, HTR1E, GLRA1, MCOLN3, CDK4, GABRG2, PLCG2, CHRNB4, PAX8, STAT1, PDE6H, HRH2, GPR55, CHRM4, TP53, GABRP, GABRE, GABRG1, LCK, IDE, ATP4A, ATP4B, P2RX3, P2RX4, ODC1, GABRR1, HGFAC, SCN5A, INSR, TLR4, TGFBR2, KCNQ2, NMBR, PSMD12, PSMD3, PSMA5, PSMD8, PSMC1, PSMD1, PSMD13, GABRA1, CCNA1, ADCYAP1R1, PTGER3, PDE3B, PDE10A, TBXA2R, GCGR, ITGB6, FPR1, PIM1, LDLR, DUT, NOTCH3, G6PD, KHK, MGAT2, PSMB2, CLK4, NFKB1, CDC25C, SLC6A9, CACNA1H, HTR7, FYN, NPC1, HDAC2, PROC, PIK3CD, CASP8, CASP3, P2RX7, APOA2, JAK2, BACE1, DPP4, CCNA2, GSTP1, MMP2, FABP7, RXRA, RARG, THRB, RARB, FGFR1, RXRB, RORA, VDR

### 3.3 Interaction Network of p‐Cymene Targets and Identification of Hub Genes

The interaction network analysis was constructed to illustrate the relationships between the identified potential targets of p‐cymene and those associated with HCC. The network comprises a total of 215 nodes, which represent the identified targets, and 1579 edges, reflecting the interactions among these targets. The average node degree was calculated to be 14.7, indicating a rich interconnectivity among the targets. To further understand the significance of these interactions, we evaluated the network using several statistical metrics. The average local clustering coefficient was found to be 0.904, suggesting a high degree of clustering among the nodes, which may indicate the presence of tightly knit groups of interacting proteins. Additionally, the PPI enrichment analysis yielded an expected number of edges of 642, with a PPI enrichment *p*‐value of < 1.0e‐16. This result indicates that the network has significantly more interactions than expected by chance, reinforcing the biological relevance of the identified targets in the context of both p‐cymene and HCC (Figure S2).

### 3.4 In Silico KEGG Pathway Analysis Suggests Potential Protective Mechanisms of p‐Cymene in HCC

To investigate the potential molecular mechanisms by which p‐cymene could exert protective effects against HCC, an in silico KEGG pathway enrichment analysis was performed. The computational analysis revealed that genes targeted by p‐cymene are potentially involved in several critical pathways related to oncogenesis, metabolic dysregulation, immune modulation, and viral infections, all of which play pivotal roles in HCC pathogenesis.

The results suggested that p‐cymene may modulate components of the pathways in cancer, indicating a potential to influence key processes such as abnormal cell proliferation, apoptosis resistance, angiogenesis, and metastasis. The involvement of the lipid and atherosclerosis pathway suggests that p‐cymene could affect lipid metabolism and inflammation, both of which are closely linked to liver cancer progression.

Moreover, p‐cymene‐associated targets were enriched in several infection‐related pathways, including Human papillomavirus infection, Hepatitis B, Hepatitis C, Kaposi sarcoma‐associated herpesvirus infection, Epstein–Barr virus infection, Influenza A, Human cytomegalovirus infection, and Toxoplasmosis. Given that chronic viral infections contribute significantly to hepatocarcinogenesis, these findings imply that p‐cymene may have a role in modulating infection‐driven inflammatory and oncogenic processes.

The analysis also highlighted involvement in pathways such as Chemical carcinogenesis—receptor activation, Drug metabolism—cytochrome P450, and the AGE‐RAGE signaling pathway in diabetic complications, suggesting that p‐cymene might reduce oxidative stress and chemical‐induced liver injury. Furthermore, the modulation of immune‐related pathways, particularly the IL‐17 signaling pathway, implies that p‐cymene could potentially regulate tumor‐associated inflammation, a known driver of HCC.

Enrichment in endocrine‐related pathways, including Endocrine resistance, Thyroid hormone signaling pathway, and Prolactin signaling pathway, points toward a possible influence of p‐cymene on hormonal signaling networks associated with liver tumor progression. Finally, the prediction of involvement in Transcriptional misregulation in cancer and Viral carcinogenesis pathways suggests that p‐cymene may have broader regulatory effects on transcriptional and virus‐related oncogenic mechanisms.

**FIGURE 2:**
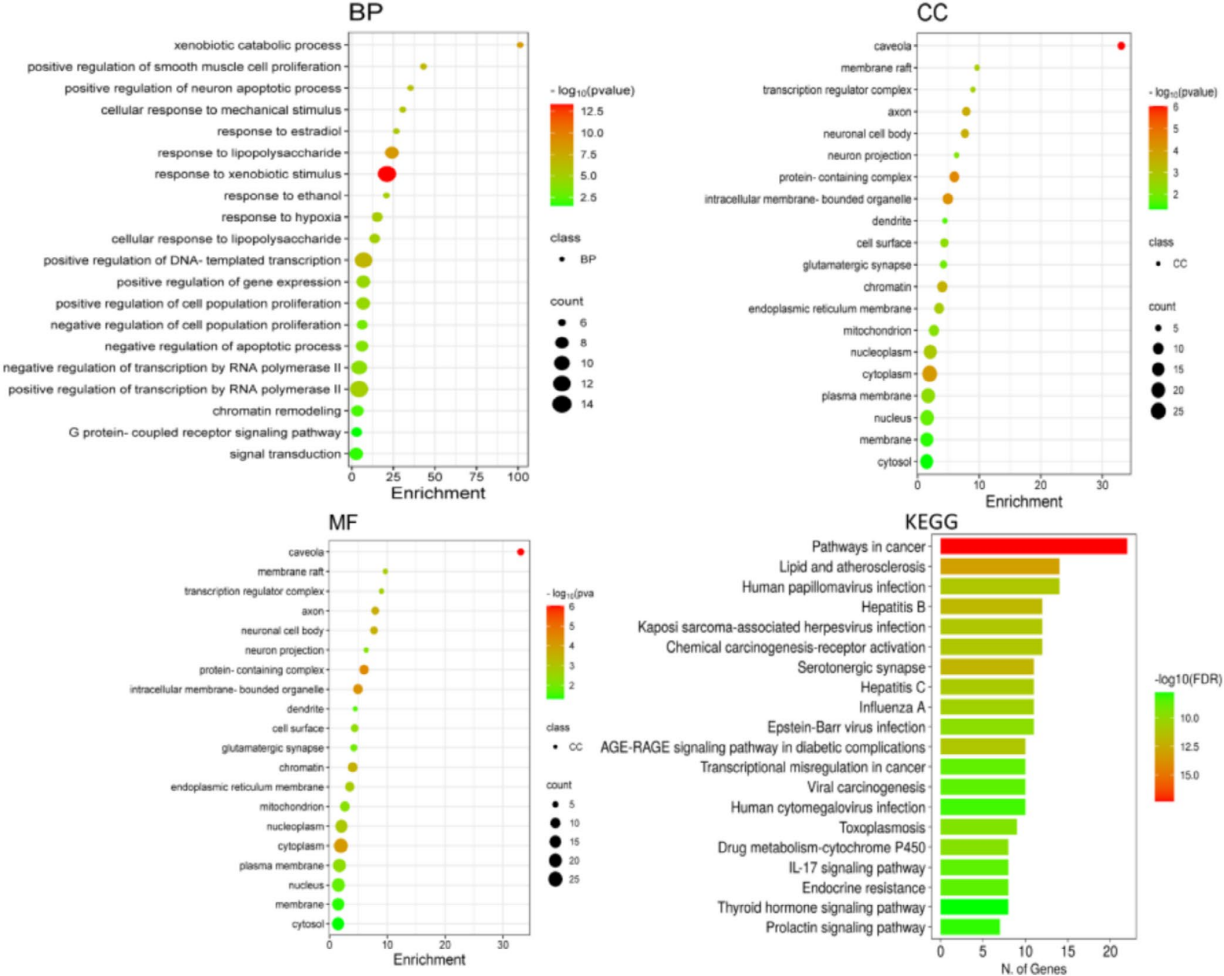
Enrichment analysis across three categories: Biological processes (BP), cellular components (CC), and molecular functions (MF), along with associated KEGG pathways. Each panel features a dot plot indicating the significance and number of genes linked to various enriched terms. The size and color of the dots represent the number of genes and the level of significance, respectively, emphasizing key pathways and functions relevant to the study, including those related to cancer and various signaling mechanisms.

Collectively, these in silico findings propose that p‐cymene may exert protective effects by targeting multiple pathways involved in carcinogenesis, metabolism, immune modulation, and infection response (Figure 2).

### 3.5 Compound Target Pathway Network Analysis

**FIGURE 3:**
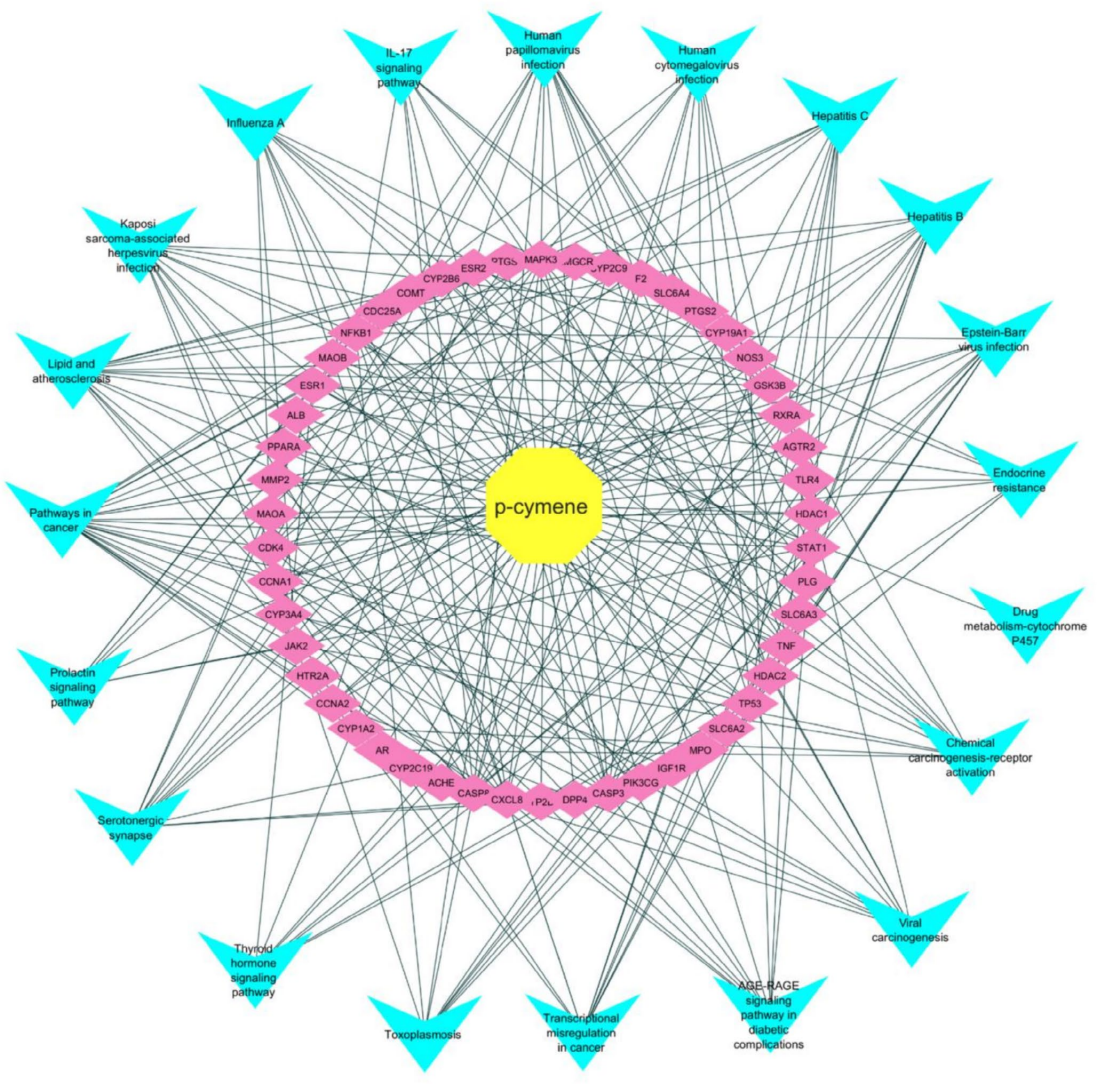
p‐Cymene's complex network of interactions. The figure displays a central yellow circle representing p‐cymene, surrounded by cyan triangular nodes that signify related entities. Thin gray lines connect these nodes, illustrating their interrelations. This starburst layout emphasizes p‐cymene's central role in a complex network of interactions.

The network analysis centered around p‐cymene, comprising 71 nodes and 261 edges, revealed a highly interconnected structure with an average of 7.352 neighbors per node, indicating efficient connectivity with a characteristic path length of 2.228 and a network radius of 2. Notably, the network exhibited moderate centralization (0.627) and high heterogeneity (0.965), suggesting significant variability in node connections (Figure 3).

### 3.6 Docking Analyses

The docking results for various proteins revealed notable interactions and binding affinities with different compounds, particularly p‐cymene and 5FU. For HIF1A (PDB ID: 1lqb), binding with p‐cymene showed an RMSD of 1.8106, indicating a moderate level of flexibility in the protein‐ligand interaction. Key interacting residues include ARG (A:68), which can contribute positively charged interactions, TYR (B:83) that can engage in π‐π stacking, and PRO (A:69) which provides structural stability. Additional residues such as MET (A:1), PHE (A:4), SER (A:65), and GLN (A:64) further stabilize the binding through hydrophobic and polar interactions. When docked with 5FU, HIF1A displayed a lower RMSD of 1.6872, illustrating a more favorable binding conformation. Interactions involved PHE (A:41), ALA (A:67), and SER (A:68), which likely engaged through hydrophobic contacts, while PRO (A:69), ASP (A:72), and ARG (A:68) contribute to a more complex interaction network, enhancing binding affinity. BCL2 (PDB ID: 2XA0) exhibited strong interactions with p‐cymene, with an RMSD of 1.1239. The interaction with residues like ARG (A:127) and PHE (A:124) suggested critical hydrophobic and ionic interactions, while PRO (A:123) added to the structural integrity of the binding site. The presence of GLY (B:128) and THR (B:125) further enhanced the interaction through their small side chains that allow for close packing. With 5FU, BCL2 showed an RMSD of 1.3077, indicating stable interactions. Notably, ALA (B:126) and THR (B:125) contributed to hydrophobic contacts, while the presence of PHE (B:124) and ARG (A:127) emphasized the importance of aromatic stacking and electrostatic attractions in stabilizing the ligand‐protein complex. CDK9 (PDB ID: 3BLR) revealed an RMSD of 0.8695 when bound to p‐cymene, suggesting a tight interaction. The critical residues ARG (A:68) and TYR (B:83) play significant roles in mediating strong ionic and hydrophobic interactions, while PRO (A:69) enhances the structure's stability. The binding with 5FU showed an even lower RMSD of 0.6697, indicating enhanced stability with interactions involving ALA (A:166) and VAL (A:79), which are likely to engage in hydrophobic interactions, complemented by residues like ASP (A:104) and PHE (A:105). JAK2 (PDB ID: 3JY9), when interacting with p‐cymene, showed an RMSD of 1.0936, with key interactions from MET (A:929) and PHE (A:995), which may form crucial hydrophobic contacts. The presence of GLY (A:993) and VAL (A:863) indicated a flexible interaction that accommodated the ligand effectively. The binding with 5FU displayed an RMSD of 1.0278, where PHE (A:995) and ILE (A:901) contributed to strong hydrophobic interactions, while the involvement of GLU (A:898) and LYS (A:999) suggested additional polar and ionic interactions. VEGF (PDB ID: 3V2A) showed an RMSD of 1.1798 with p‐cymene, with interactions from PRO (A:49) and ILE (R:215) suggesting hydrophobic interactions, while LYS (A:48) and MET (A:81) contributed to ionic and hydrophobic stabilization. The binding with 5FU has an RMSD of 1.2168, with PHE (A:47) and SER (A:50) enhancing the interaction through hydrophobic contacts. For MAPK4 (PDB ID: 4ZP5), the binding with p‐cymene presented an RMSD of 0.5342, indicating a strong interaction profile. The residues ASP (B:153), ARG (B:180), and MET (B:195) contributed to various interactions, including hydrogen bonds and Van der Waals forces. The binding with 5FU revealed an RMSD of 0.7788, with ILE (B:154) and ASP (B:153) forming critical hydrophobic interactions. P53 (PDB ID: 5O1H) displayed an RMSD of 1.4004 with p‐cymene, highlighting significant interactions from VAL (B:197) and ASP (B:186), which may form essential ionic or polar contacts. The binding with 5FU had a slightly lower RMSD of 1.0336, with interactions from LEU (B:201) and ASN (B:200) emphasizing the role of hydrophobic interactions in stabilizing the ligand‐protein complex. STAT3 (PDB ID: 6NJS) docking results with p‐cymene exhibited an RMSD of 0.5593, indicating a stable binding conformation. Key interacting residues include ASP (A:334), which can form hydrogen bonds, and LYS (A:573), which may contribute positively charged interactions that enhance binding stability. Additionally, ILE (A:569) and ARG (A:335) played significant roles through hydrophobic interactions, while ASP (A:570) and ASP (A:566) further stabilized the complex with their polar side chains, facilitating effective ligand binding through a network of ionic and hydrogen bonds. When bound to 5FU, STAT3 showed an RMSD of 1.0315, suggesting a slightly less stable interaction compared to p‐cymene. The critical residues in this case were again ASP (A:566) and ARG (A:335), which are involved in strong ionic interactions. The presence of ASP (A:570) and LYS (A:573) continues to emphasize the importance of polar interactions in maintaining the structural integrity of the binding site, ensuring effective ligand engagement. CASP3 (PDB ID: 1NME) docking with p‐cymene yielded an RMSD of 2.3805, indicating a more flexible interaction profile. The key interacting residues included GLN (A:161) and ALA (A:162), which are likely involved in hydrophobic interactions that stabilize the binding. SER (A:120) contributed to hydrogen bonding, while ARG (A:64) enhanced the interaction through its positive charge, allowing for ionic interactions. GLY (A:122) and CYS (A:163) further supported the binding through their small and reactive side chains, respectively, which can accommodate the ligand structure.

**FIGURE 4:**
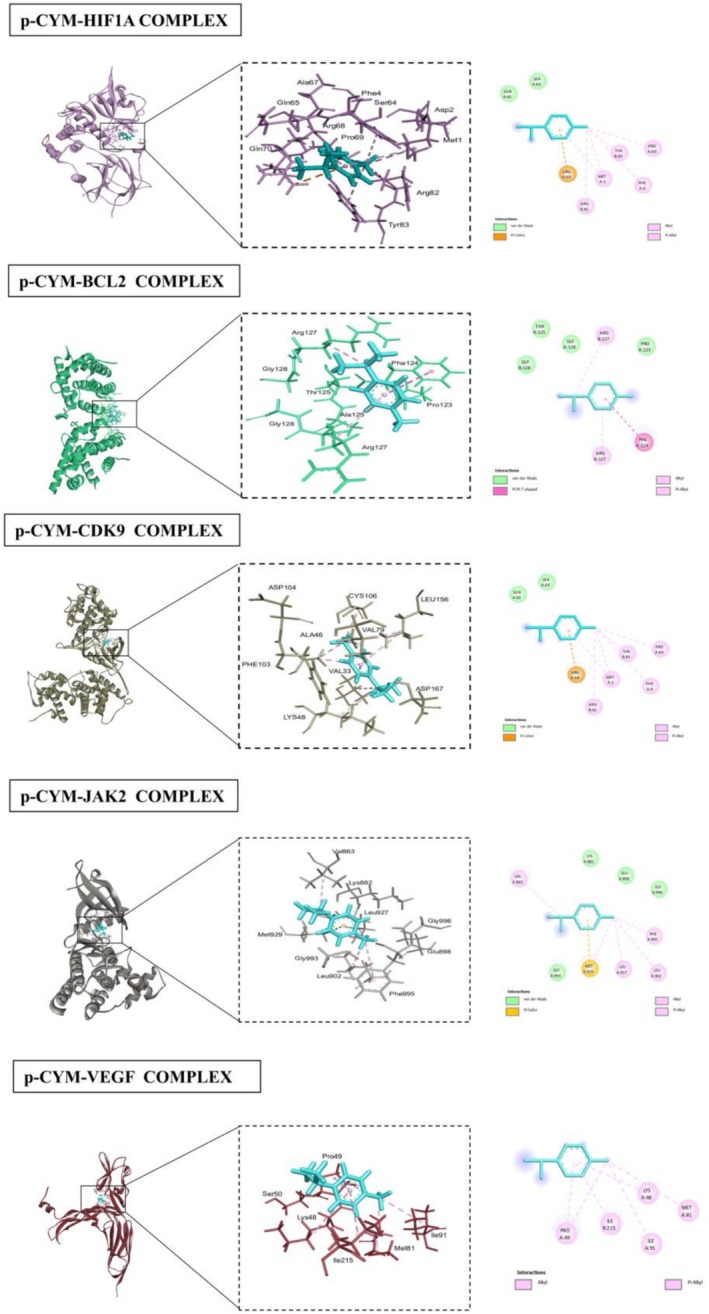
Interactions of p‐cymene with human proteins (HIF1A, BCL2, CDK9, JAK2, VEGF), highlighting key residues and binding sites.

**FIGURE 5:**
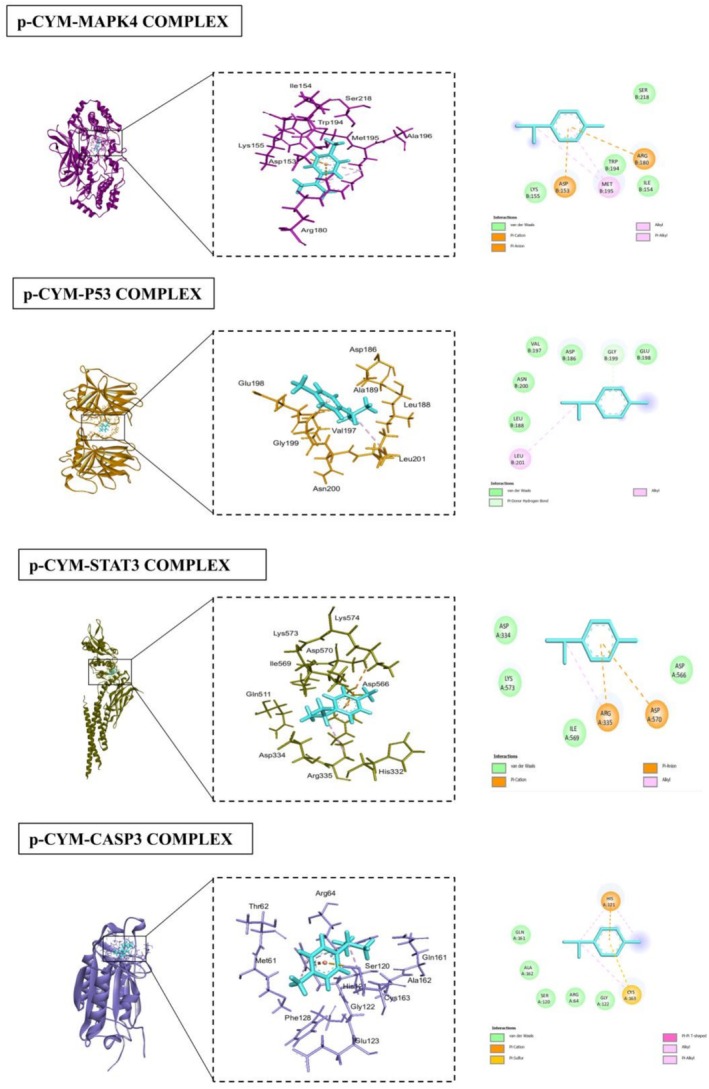
Interactions of p‐cymene with human proteins (MAPK4, P53, STAT3, CASP3), highlighting key residues and binding sites.

**FIGURE 6:**
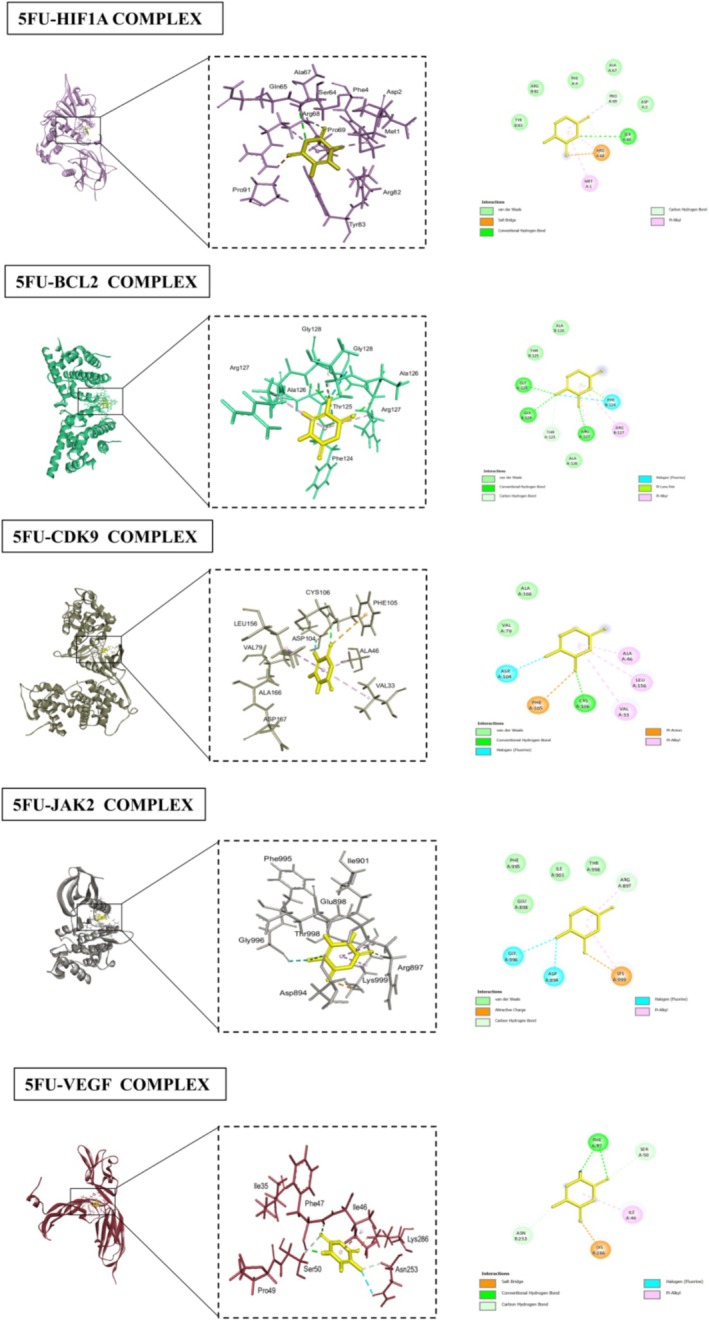
Interactions between 5FU and various human proteins (HIF1A, BCL2, CDK9, JAK2, VEGF), highlighting key residues and binding sites.

**FIGURE 7:**
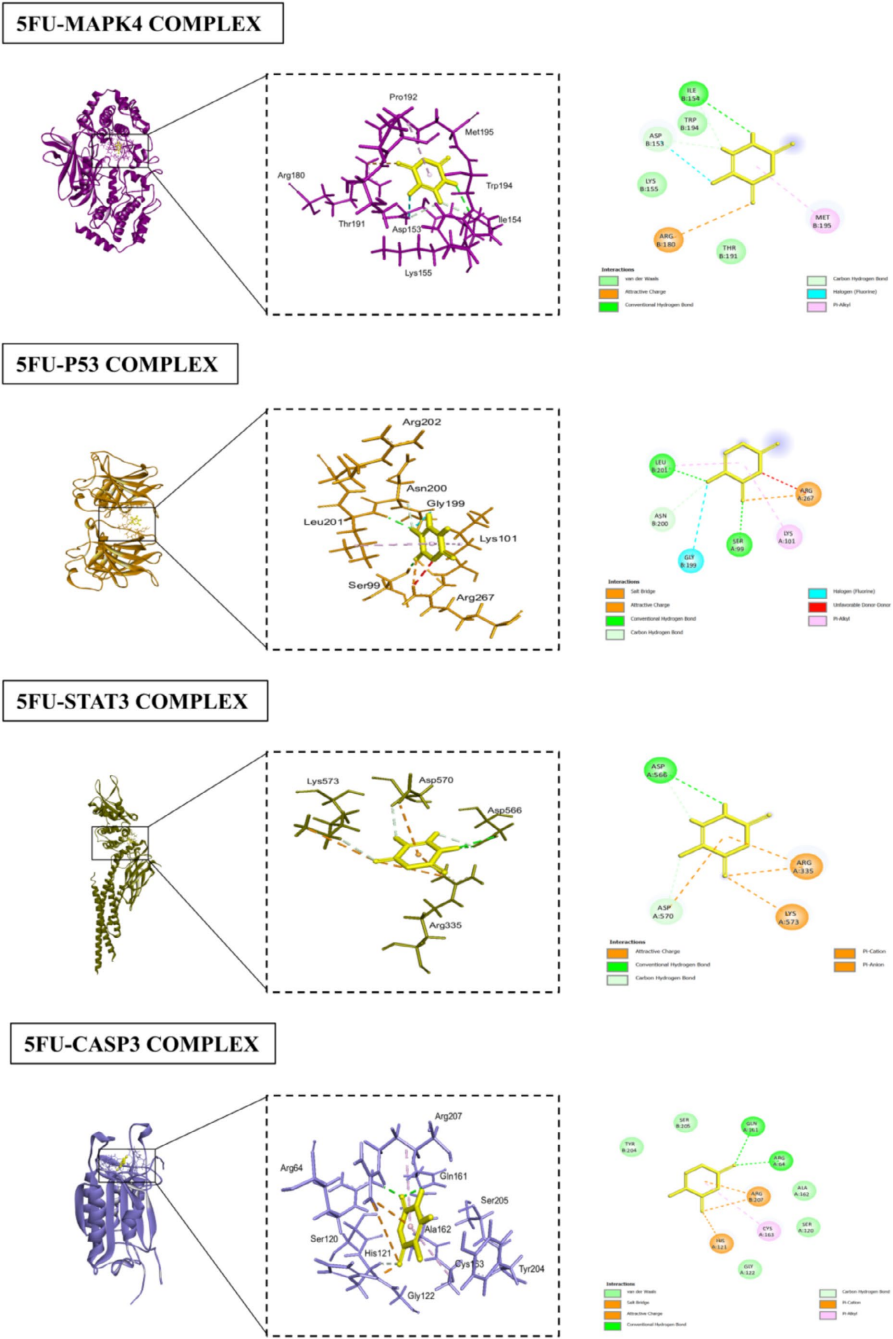
Interactions between 5FU and various human proteins (MAPK4, P53, STAT3, CASP3), highlighting key residues and binding sites.

When CASP3 interacted with 5FU, the RMSD dropped to 1.4584, suggesting a more favorable binding conformation. Important residues in this scenario included TYR (B:204) and SER (B:205), which likely engaged in strong hydrophobic interactions. GLN (A:64) and ARG (A:207) continued to provide stabilization through ionic interactions, while ALA (A:162) and CYS (A:163) enhanced the binding through their respective hydrophobic and reactive properties. Overall, these interactions highlight the versatility of CASP3 in accommodating different ligands while maintaining a stable binding environment (Table 2; Figures 4, 5, 6, 7).

**TABLE 2:** The binding affinities and interacting residues of p‐cymene and 5FU with various proteins highlighting their respective *S* scores and RMSD values.

Protein (PDB ID)	Compound	*S* score	RMSD refine	Interacting residues
HIF1A (1lqb)	p‐CYM	−4.3949	1.8106	ARG (A:68), TYR (B:83), PRO (A:69), MET (A:1), PHE (A:4), SER (A:65), GLN (A:64)
	5FU	−4.0570	1.6872	PHE (A:41), ALA (A:67), SER (A:68) PRO (A:69), ASP (A:72), ARG (A:68) MET (A:1), TYR (A:83)
BCL2 (2XA0)	p‐CYM	−4.4959	1.1239	ARG (A:127), PHE (A:124), PRO (A:123), GLY (B:128), GLY (A:125), THR (B:125)
	5FU	−4.0735	1.3077	ALA (B:126), THR (B:125), GLY (B:128), ARG (A:127), PHE (B:124), GLY (B:128), THR (A:125)
CDK9 (3BLR)	p‐CYM	−4.7124	0.8695	ARG (A:68), TYR (B:83), PRO (A:69), MET (A:1), PHE (A:4), ARG (B:82)
	5FU	−3.8691	0.6697	ALA (A:166), VAL (A:79), LEU (A:156), ASP (A:104), PHE (A:105), CYS (A:106), VAL (A:33), ALA (A:46)
JAK2 (3JY9)	p‐CYM	−4.5740	1.0936	MET (A:929), PHE (A:995), LEU (A:927), LEU (A:925), GLY (A:993), VAL (A:863), GLU (A:996)
	5FU	−4.2466	1.0278	PHE (A:995), ILE (A:901), THR (A:998), ARG (A:897), GLU (A:898), ASP (A:894), GLY (A:996), LYS (A:999)
VEGF (3V2A)	p‐CYM	−4.3485	1.1798	PRO (A:49), ILE (R:215), LYS (A:48), MET (A:81), ILE (A:91)
	5FU	−4.0227	1.2168	PHE (A:47), SER (A:50), ILE (A:46), ASN (R:253), LYS (R:286)
MAPK4 (4ZP5)	p‐CYM	−4.8946	0.5342	ASP (B:153), ARG (B:180), MET (B:195), LYS (B:155), TRP (B:194), SER (B:218)
	5FU	−4.5123	0.7788	ILE (B:154), ASP (B:153), LYS (B:155), ARG (B:180), TRP (B:154), MET (B:195), THR (B:191)
P53 (5O1H)	p‐CYM	−4.6948	1.4004	VAL (B:197), ASP (B:186), GLY (B:199), GLU (B:198), ASN (B:200), LEU (B:188), LEU (B:201)
	5FU	−4.2001	1.0336	LEU (B:201), ASN (B:200), GLY (B:199), SER (A:99)
STAT3 (6NJS)	p‐CYM	−4.2689	0.5593	ASP (A:334), LYS (A:573), ILE (A:569), ARG (A:335), ASP (A:570), ASP (A:566)
	5FU	−3.6239	1.0315	ASP (A:566), ARG (A:335), ASP (A:570), LYS (A:573)
CASP3 (1NME)	p‐CYM	−4.6482	2.3805	GLN (A:161), ALA (A:162), SER (A:120), ARG (A:64), GLY (A:122), CYS (A:163), HIS (A:121)
	5FU	−4.5401	1.4584	TYR (B:204), SER (B:205), GLN (A:64), ARG (A:207), ALA (A:162), CYS (A:163), HIS (A:121), GLY (A:122)

**FIGURE 8:**
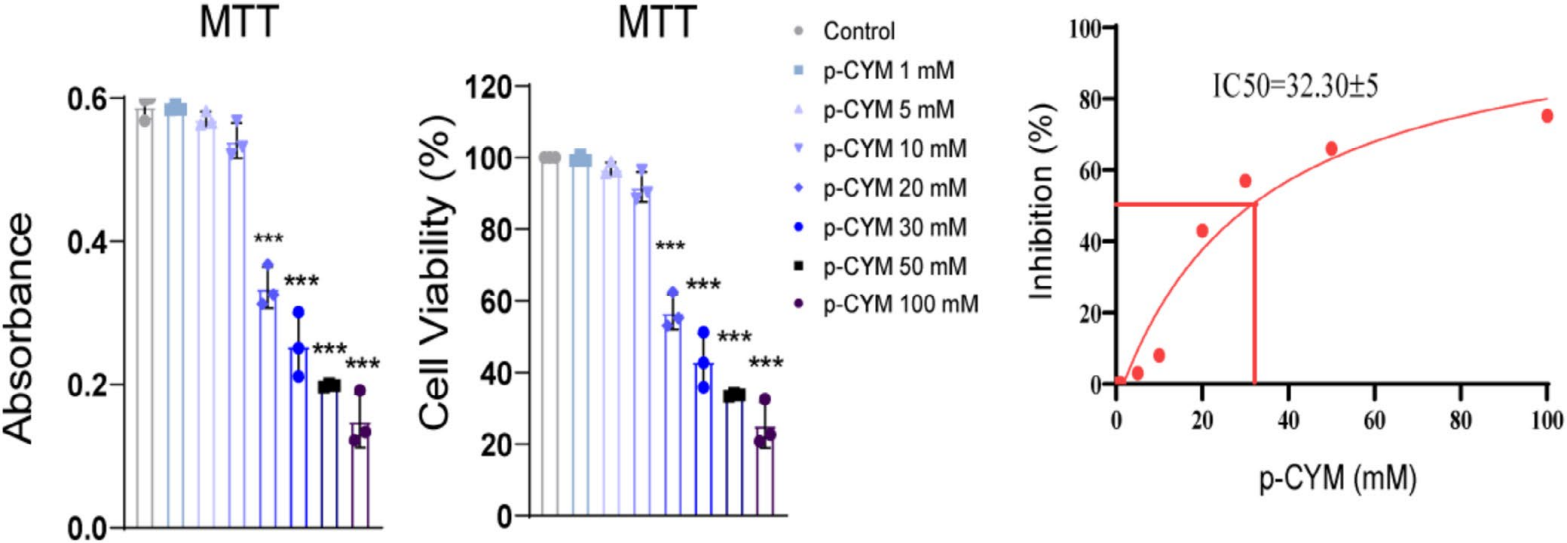
Half‐maximal inhibitory concentration of p‐cymene. p‐cymene dose dependently attenuated cell viability with an IC_50_ value of 32 mM. One‐way ANOVA followed by Dunnett's multiple comparison test was used to analyze the data, confirming the significant differences among the treatment groups. Significance levels were denoted as: ***p ≤ 0.001 (treated groups vs. negative control).

**FIGURE 9:**
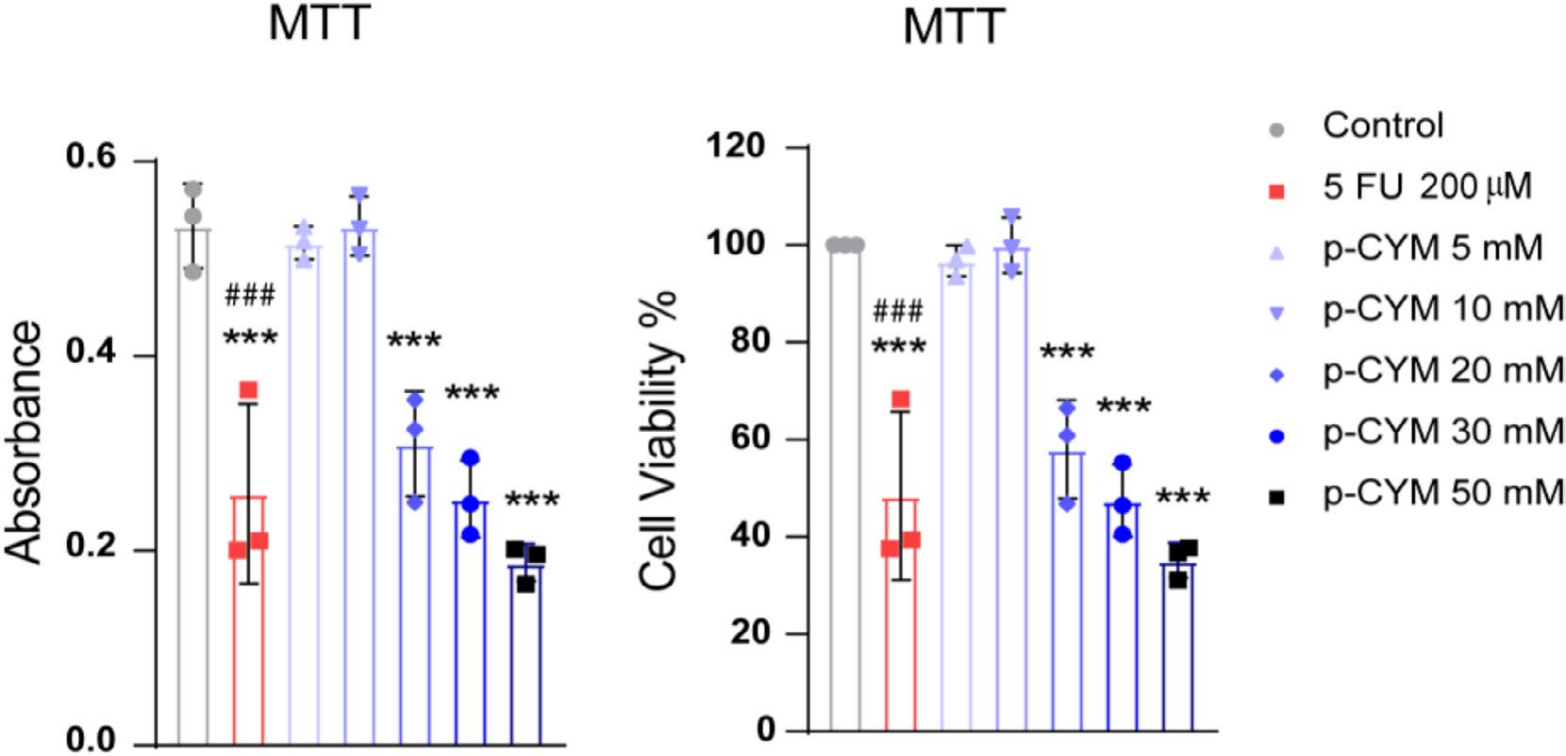
MTT assay assessing the impact of p‐cymene on HepG2 cell viability. The absorbance values indicate that p‐cymene treatment significantly reduces cell viability in a dose‐dependent manner compared to the control group. One‐way ANOVA followed by Dunnett's multiple comparison test was used to analyze the data, confirming the significant differences among the treatment groups. Significance levels were denoted as: ***p ≤ 0.001 (treated groups vs. negative control); ^###^ *p* ≤ 0.001 (positive control vs. negative control).

**FIGURE 10:**
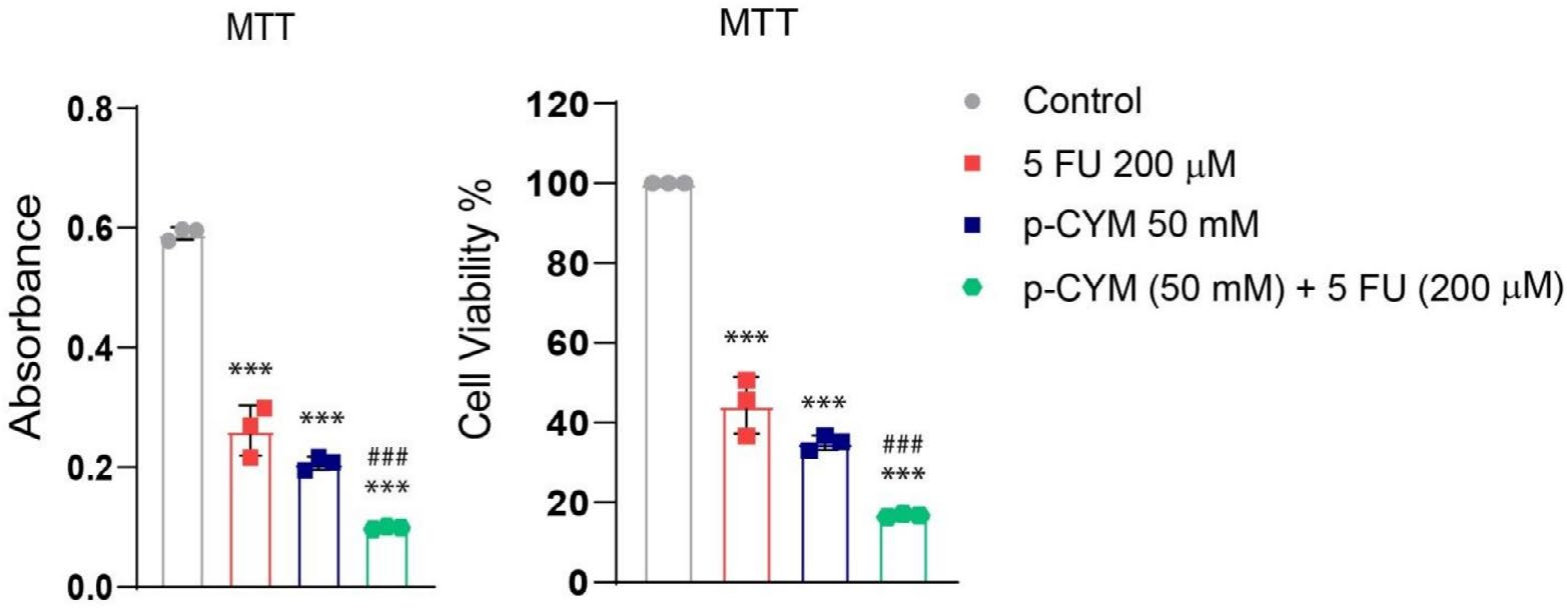
Effects of 5FU, p‐cymene, and their combination on cell viability assessed by MTT assay. Both single‐agent treatments significantly reduced cell viability compared to control (***p < 0.001), while the combination treatment further enhanced cytotoxicity, suggesting a potential synergistic effect. One‐way ANOVA followed by Dunnett's multiple comparison test was used to analyze the data, confirming the significant differences among the treatment groups. Significance levels were denoted as: ***p ≤ 0.001 (treated groups vs. negative control); ^###^ *p* ≤ 0.001 (positive control vs. negative control).

**FIGURE 11:**
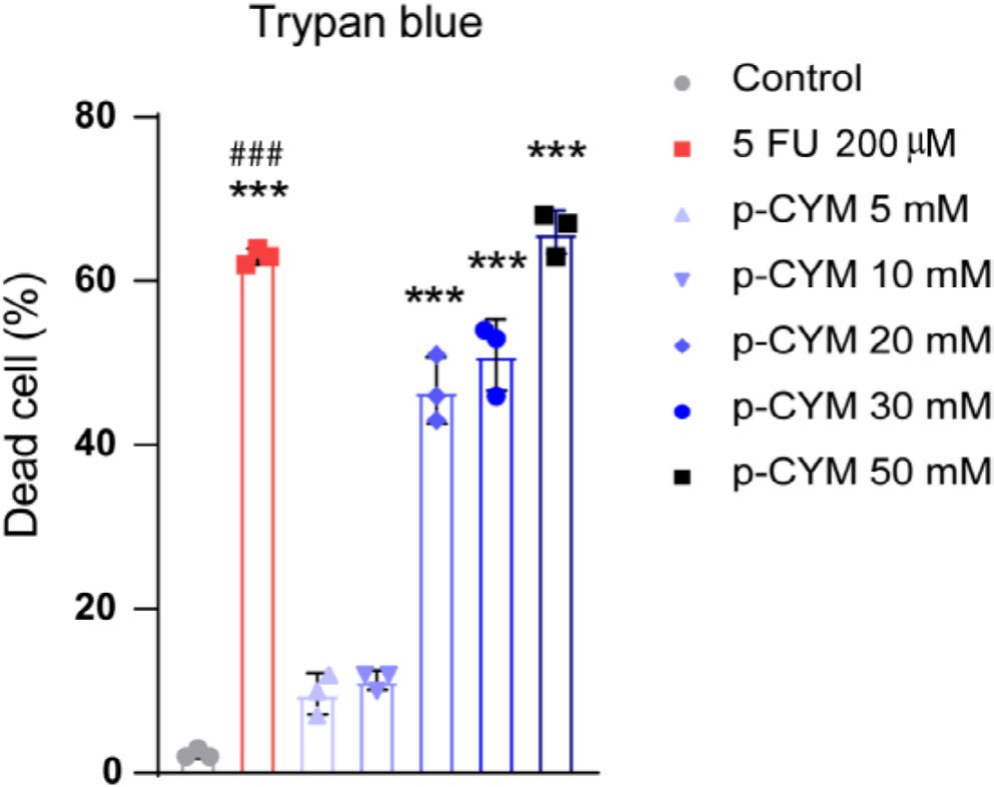
Trypan blue exclusion assay assessing cell death in HepG2 cell lines following treatment with p‐cymene. The data show a significant increase in the percentage of dead cells at higher concentrations of p‐cymene compared to the control group. Statistical analysis was conducted using one‐way ANOVA followed by Dunnett's multiple comparison test to confirm significant differences among the treatment groups. Significance levels were denoted as: ***p ≤ 0.001 (treated groups vs. negative control); ^###^ *p* ≤ 0.001 (positive control vs. negative control).

**FIGURE 12:**
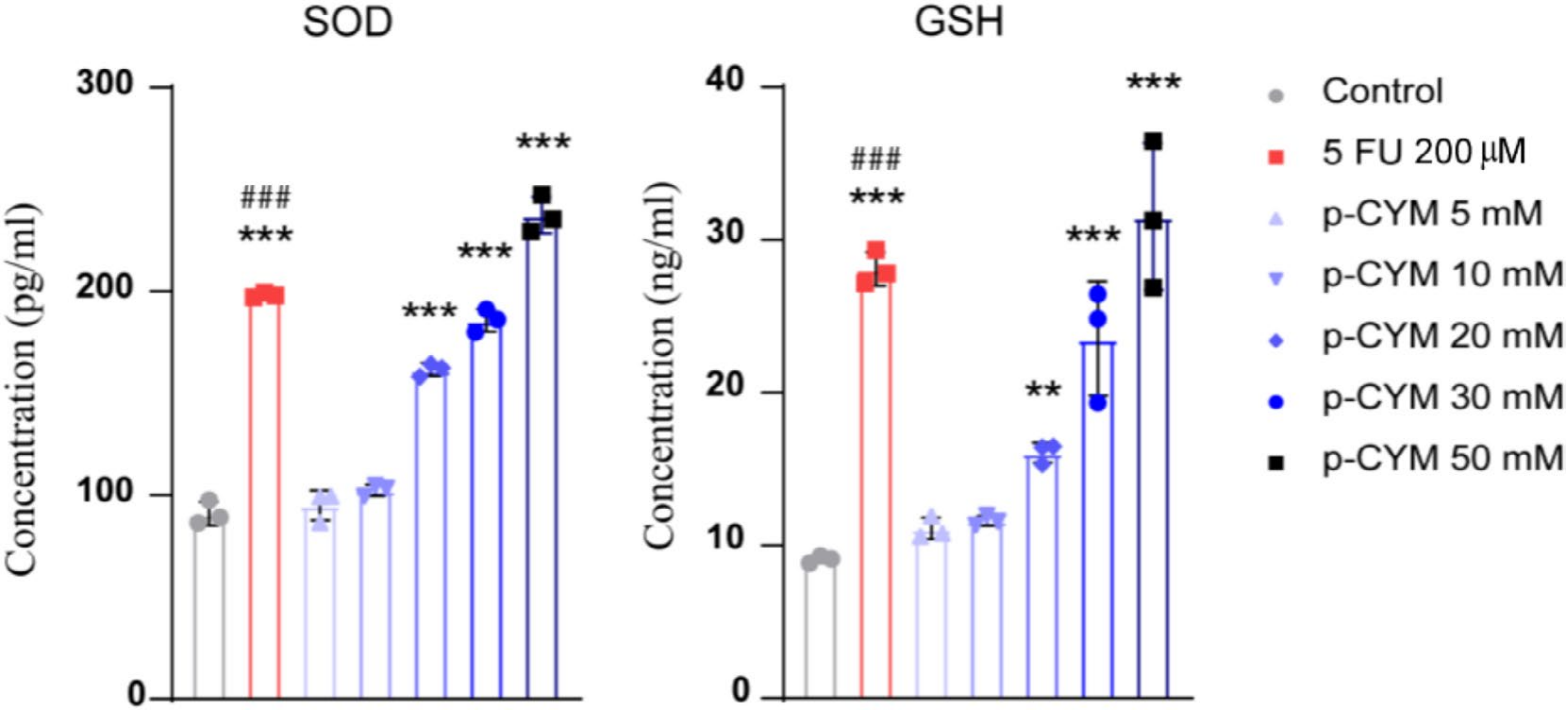
Effects of p‐cymene on the levels of SOD and GSH. A significant increase in SOD and GSH levels is observed at higher doses of p‐cymene. One‐way ANOVA followed by Dunnett's multiple comparison test. Significance levels were denoted as: ***p ≤ 0.001, **p ≤ 0.01 (treated groups vs. negative control); ^###^ *p* ≤ 0.001 (positive control vs. negative control).

**FIGURE 13:**
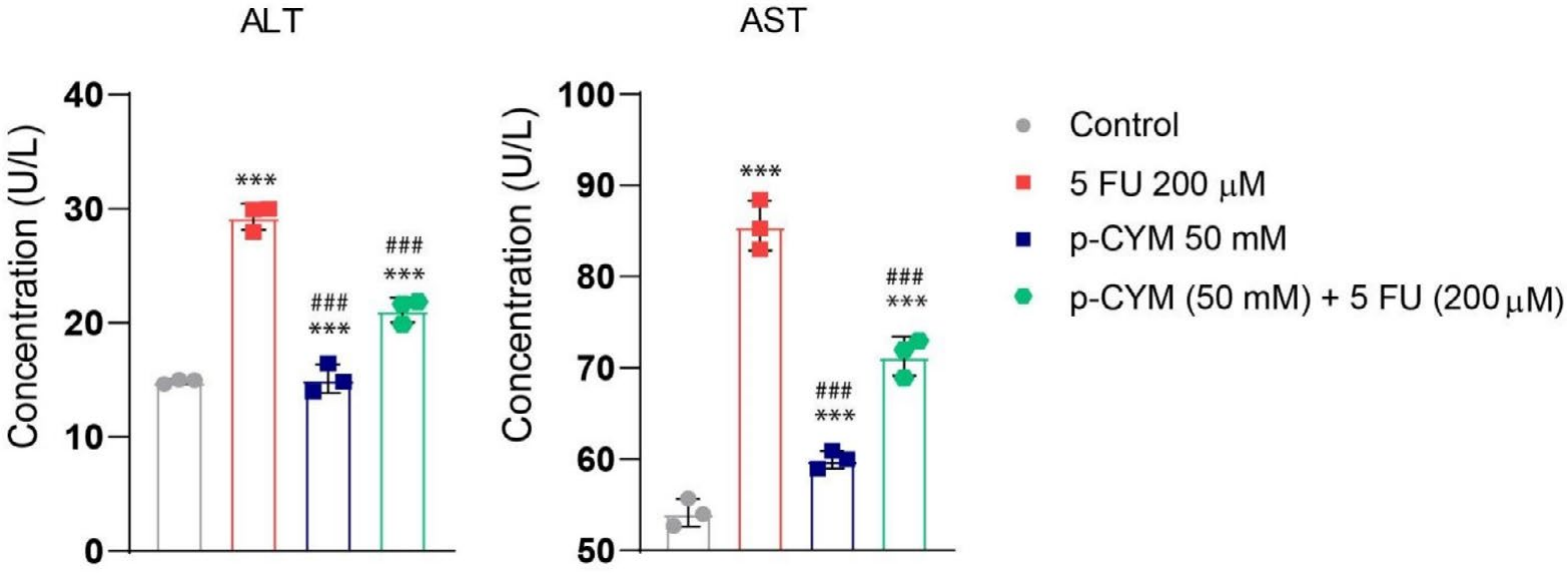
p‐cymene ameliorated the hepatotoxic effects of 5FU. Serum levels of ALT and AST were measured to assess hepatic injury after exposure to 5FU, p‐CYM, or their combination. 5FU treatment significantly elevated ALT and AST levels compared to the control group, indicating hepatocellular damage. Co‐treatment with p‐cymene and 5FU resulted in significantly lower enzyme levels than 5FU alone (***p < 0.001), suggesting a possible hepatoprotective effect. One‐way ANOVA followed by Dunnett's multiple comparison test. Significance levels were denoted as: ***p ≤ 0.001 (treated groups vs. negative control); ^###^ *p* ≤ 0.001 (positive control vs. negative control).

**FIGURE 14:**
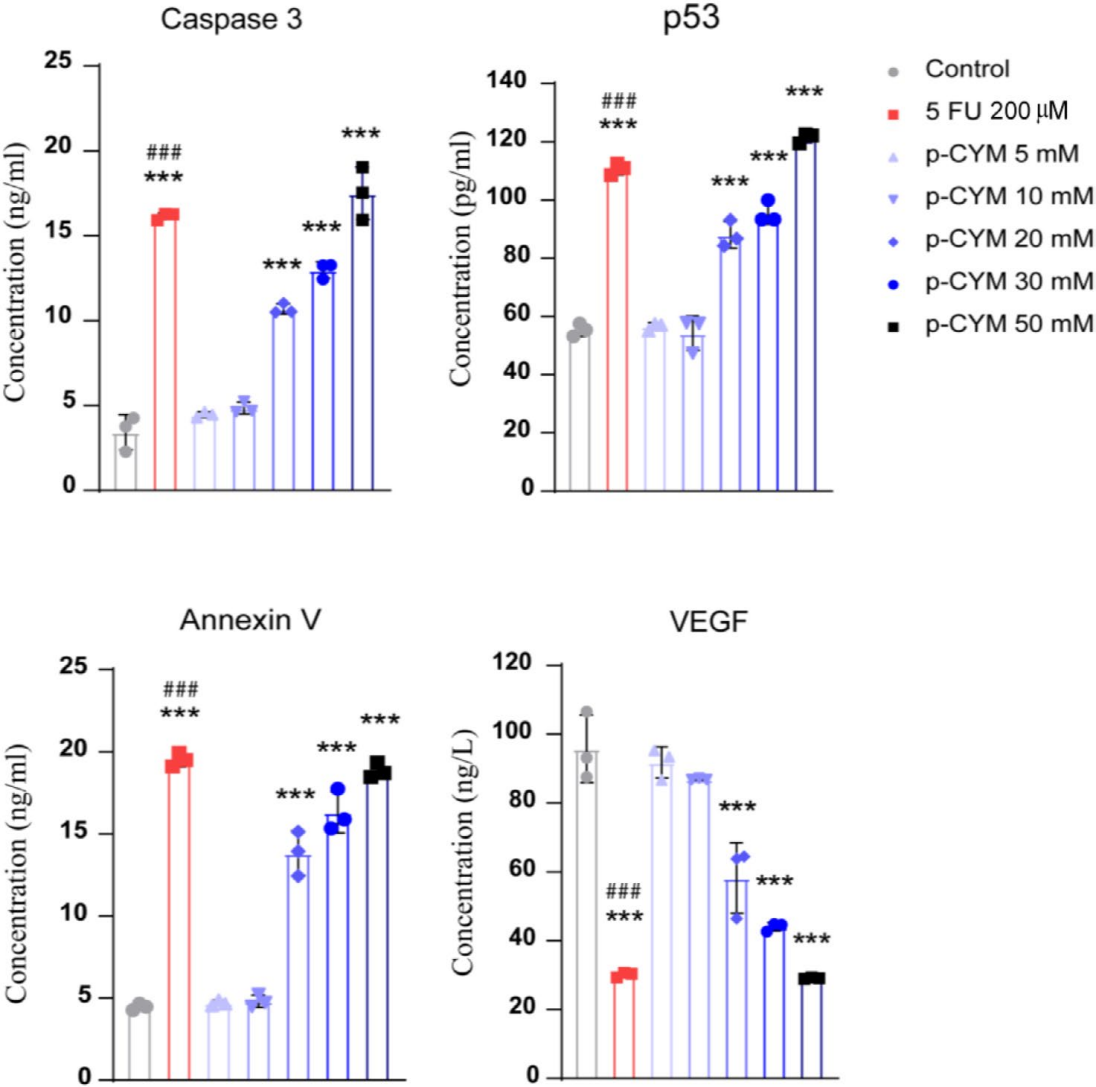
Effects of p‐cymene on the levels of CASP3, P53, Annexin‐V, and VEGF in HepG2 cells. Significant differences among groups were determined using one‐way ANOVA followed by Dunnett's multiple comparison test. Significance levels were denoted as: ***p ≤ 0.001 (treated groups vs. negative control); ^###^ *p* ≤ 0.001 (positive control vs. negative control).

### 3.7 p‐Cymene and 5FU Reduced Cell Viability in Concentration Dependent Fashion

In the experiments conducted on HepG2 liver cancer cells, both p‐cymene and 5FU treatments were evaluated for their cytotoxic effects using the MTT assay, which measures cell viability based on metabolic activity. p‐Cymene displayed a dose‐dependent reduction in cell viability, where higher concentrations of the compound significantly reduced cell survival. This suggests that p‐cymene exerts moderate cytotoxic effects, with an estimated IC_50_ of 32 mM. Though not as potent as conventional chemotherapy, these results indicate that p‐cymene has potential anticancer properties worth further exploration (Figure 8).

In contrast, 5FU treatment demonstrated a much stronger cytotoxic response, with cell viability sharply decreasing even at low concentrations. The estimated IC₅₀ for 5FU was 168 μM, consistent with its established use as a powerful chemotherapeutic agent. When compared, 5FU is clearly more effective at inhibiting HepG2 cell growth, but p‐cymene may offer benefits such as lower toxicity or suitability in combination therapies (Figure S3).

### 3.8 Impact of p‐Cymene on HepG2 Cell Viability

The MTT assay results demonstrated that p‐cymene significantly reduced the viability of HepG2 cells in a dose‐dependent manner compared to the control group. The absorbance measurements indicated that the control group exhibited the highest metabolic activity, correlating with robust cell proliferation, while treatments with p‐cymene at concentrations of 20, 30, and 50 mM led to a marked decrease in absorbance and cell viability percentages. Notably, higher concentrations of p‐cymene (30 and 50 mM) resulted in a significant reduction in cell viability (Figure 9).

The results from the crystal violet assay also revealed that p‐cymene significantly impaired the viability of HepG2 cells in a concentration‐dependent manner compared to the control group. The left panel displays absorbance values, where the control group showed the highest absorbance, indicating robust cell proliferation. In contrast, treatments with p‐cymene at concentrations of 20, 30, and 50 mM exhibited a marked decrease in absorbance, reflecting reduced cell viability (Figure S4). Overall, these findings suggest that p‐cymene possesses a potent cytotoxic effect on HepG2 cells, warranting further investigation into its therapeutic potential against HCC.

### 3.9 Synergistic Action of p‐Cymene and 5FU on Cell Viability

The cytotoxic effects of 5FU, p‐cymene, and their combination were assessed using the MTT assay. As illustrated in Figure 10, treatment with 5FU or p‐cymene alone led to a statistically significant reduction in cell viability compared to the untreated control group (*p* < 0.001). Specifically, cells treated with 5FU exhibited a reduction in absorbance and viability to approximately 45%, whereas p‐cymene treatment alone decreased viability to around 35%.

Interestingly, the co‐administration of p‐cymene and 5FU resulted in a more pronounced cytotoxic effect, reducing cell viability to nearly 15%. This combination treatment demonstrated a statistically significant difference not only from the control (*p* < 0.001), but also from 5FU, indicating a synergistic interaction between p‐cymene and 5FU.

These findings suggest that p‐cymene may enhance the cytotoxic potential of 5FU, possibly through synergistic mechanisms that warrant further mechanistic investigation.

### 3.10 p‐Cymene Induced Cell Death in HepG2 Cells: Trypan Blue Exclusion Assay

The trypan blue exclusion assay results indicated that p‐cymene induced a dose‐dependent increase in cell death in HepG2 cells. The control group exhibited the lowest percentage of dead cells, confirming the baseline viability of untreated cells. As p‐cymene concentration increased (30 and 50 mM), a significant rise in cell mortality was observed (***p < 0.001) compared to the control group. This dose–response relationship suggests that p‐cymene progressively compromises cell viability, leading to cytotoxicity. The trypan blue assay, which differentiates live from dead cells based on membrane integrity, further supports the conclusion that p‐cymene disrupts cellular homeostasis and promotes cell death in a concentration‐dependent manner (Figure 11).

### 3.11 Impact of p‐Cymene on Antioxidant Levels in HepG2 Cells: SOD and GSH Measurements

Treatment with p‐cymene significantly affected the antioxidant defense system in HepG2 cells, as indicated by changes in SOD and GSH levels. SOD, a key enzyme that neutralizes harmful superoxide radicals, shows a dose‐dependent redundant rise with p‐cymene. This induction in SOD implies that HepG2 cells treated with p‐cymene have a capacity to neutralize oxidative stress by scavenging reactive oxygen species (ROS).

Similarly, GSH, a crucial antioxidant responsible for detoxifying ROS and maintaining cellular redox balance, also increased in response to p‐cymene treatment. The rise was particularly pronounced at higher doses, with a significant reduction observed at 20 mM (Figure 12). Oxidative stress (excess ROS) is a major driver of HCC. It promotes DNA damage, mutations, inflammation, and ultimately cancer (Fu and Chung 2018; Mooli et al. 2022; Reuter et al. 2010). By increasing GSH and SOD, p‐cymene enhances the antioxidant defense system in liver cells (HepG2 cells). This reduces oxidative damage, protects DNA, inhibits chronic inflammation, and prevents oncogenic transformation—key steps in stopping or slowing HCC development.

In addition, KEGG analysis also indicated pathways related to chemical carcinogenesis, AGE‐RAGE signaling, and cytochrome P450 metabolism, which are closely tied to oxidative stress. Thus, the experimental finding (GSH and SOD upregulation) matches and supports the pathways predicted by the in silico study.

### 3.12 p‐Cymene Attenuates 5FU‐Induced Hepatotoxicity as Evidenced by ALT and AST Activity

To evaluate potential hepatotoxic effects associated with the tested compounds, serum levels of alanine aminotransferase (ALT) and aspartate aminotransferase (AST) were measured. The administration of 5FU resulted in a marked elevation of both ALT and AST levels compared to the control group, indicating a prominent hepatic injury. Specifically, ALT levels increased to approximately 30 U/L, and AST levels reached ~87 U/L following 5FU exposure. In contrast, treatment with p‐cymene alone led to significantly lower ALT and AST values (~17 U/L and ~60 U/L, respectively) compared to the 5FU group, suggesting a more favorable hepatic profile (*p* < 0.001 vs. 5FU).

Interestingly, the combination of p‐cymene and 5FU yielded intermediate enzyme levels (ALT at ~22 U/L and AST at ~73 U/L), both significantly lower than those observed with 5FU alone (*p* < 0.001), yet higher than p‐cymene alone (Figure 13). These findings imply that p‐cymene may exert a hepatoprotective effect when co‐administered with 5FU, partially mitigating 5FU‐induced hepatic damage.

### 3.13 Effects of p‐Cymene on Apoptotic and Tumor‐Suppressor Pathways in HepG2 Cell Line: Analysis of CASP3, P53, Annexin V, and VEGF

The results indicated that p‐cymene significantly modulated apoptotic and tumor‐suppressor pathways in HepG2 cells, as demonstrated by changes in key molecular markers. CASP3, a crucial executor of apoptosis, showed a dose‐dependent increase following p‐cymene treatment (EC_50_ = 27.68 mM), with particularly significant elevations at higher concentrations (***p < 0.001). This suggests that p‐cymene enhanced apoptotic activity by triggering caspase‐dependent cell death mechanisms. The activation of CASP3 is a hallmark of apoptosis, as it plays a central role in dismantling cellular components and facilitating programmed cell death. The observed increase in CASP3 levels implies that HepG2 cells are undergoing apoptosis in response to p‐cymene exposure.

In parallel, the tumor suppressor protein (P53) also exhibited a significant upregulation, particularly at concentrations of 20 mM and above (EC_50_ = 26.21 mM). P53 serves as a critical regulator of apoptosis and cell cycle arrest in response to cellular stress, including DNA damage and oxidative stress. Its increased expression in p‐cymene‐treated HepG2 cells suggests activation of P53‐dependent apoptotic pathways, further supporting the role of p‐cymene in promoting cell death. Additionally, Annexin V staining analysis revealed a substantial increase in apoptotic cells at higher concentrations (EC_50_ = 16.87 mM), reinforcing the conclusion that p‐cymene induces apoptosis rather than necrosis. Conversely, VEGF, a key factor involved in angiogenesis, was significantly downregulated following p‐cymene treatment (IC_50_ = 20.22 mM), suggesting that p‐cymene might impair the angiogenic potential of HepG2 cells. Reduced VEGF levels indicate possible inhibition of tumor‐associated blood vessel formation, which is crucial for sustaining cancer cell growth and metastasis. Collectively, these findings suggest that p‐cymene induces apoptosis through CASP3 activation, P53 upregulation, and Annexin V‐positive cell accumulation while simultaneously suppressing pro‐angiogenic signaling, highlighting its potential as an anticancer agent targeting both survival and proliferative pathways in liver cancer cells (Figure 14).

## 4 Discussion

HCC remains one of the most prevalent and lethal forms of liver cancer, necessitating the urgent exploration of novel therapeutic strategies (Bruni et al. 2024; Dorochowicz et al. 2023). Despite advancements in treatment modalities, HCC is often diagnosed at an advanced stage, limiting the efficacy of conventional therapies (Jiang, Meng, et al. 2024). Our study therefore investigated the therapeutic potential of p‐cymene in the context of HCC, particularly its ability to induce apoptosis and modulate key signaling pathways involved in tumor progression.

We employed a network pharmacology approach to elucidate the interactions between p‐cymene and potential molecular targets associated with HCC. By utilizing bioinformatics tools, we identified 635 potential targets of p‐cymene, refining this list to 216 overlapping targets with HCC‐related proteins. The interconnectivity among these targets, highlighted by PPI network analysis, suggests that p‐cymene may exert its anticancer effects through multiple signaling pathways. This multifaceted mechanism of action is particularly relevant in cancer treatment, where targeting multiple pathways can enhance therapeutic efficacy and reduce the likelihood of resistance (Garg et al. 2024). The significant clustering coefficient and PPI enrichment p‐value reinforce the biological relevance of these interactions (Zhang et al. 2021), indicating that p‐cymene could function as a multi‐targeted therapeutic agent in HCC.

In our study, p‐cymene exhibited significant cytotoxic effects against HepG2 cells at concentrations of 20–50 mM. Similarly, previous investigations have reported cytotoxic activities of essential oils containing p‐cymene or rich in related monoterpenes. For example, the leaf essential oil of *Lippia gracilis* Schauer, which contains p‐cymene among its components, demonstrated notable cytotoxicity against tumor cells, though often requiring relatively high concentrations (Ferraz et al. 2013). Likewise, essential oils of *Satureja thymbra* and *Satureja parnassica* and their major constituents, including p‐cymene, exhibited moderate antiproliferative effects (Fitsiou et al. 2016), with IC_50_ values ranging from hundreds of micromolar to millimolar levels. These findings align with our observations, suggesting that while p‐cymene possesses anticancer potential, it generally requires higher doses compared to conventional chemotherapeutics.

Although p‐cymene exhibited significant cytotoxic effects against HepG2 cells only at higher concentrations (20–50 mM) compared to 5FU (200 μM), this finding can be rationalized by the inherent differences between natural and synthetic compounds. As a plant‐derived monoterpene, p‐cymene is characterized by a generally low toxicity profile and biocompatibility, often requiring higher doses to exert cytotoxic effects compared to synthetic chemotherapeutic agents (De Oliveira et al. 2019; Miguel 2010). Moreover, p‐cymene is likely to modulate multiple pathways including oxidative stress, metabolic regulation, and immune signaling rather than directly inhibiting DNA synthesis like 5FU, accounting for its comparatively milder cytotoxicity. Importantly, the safety profile of p‐cymene suggests potential for use as an adjunctive therapeutic agent or for further optimization through advanced drug delivery systems to enhance its bioavailability and efficacy.

Cell viability assays, including MTT, Crystal Violet, and Trypan Blue exclusion assays, consistently indicated a reduction in cell proliferation and metabolic activity following p‐cymene treatment. The reduction in absorbance in the MTT assay suggests a decrease in mitochondrial function, an essential indicator of cell viability. Likewise, crystal violet staining confirmed a substantial decline in cell density, corroborating the cytotoxic effects observed in the MTT assay. The Trypan Blue exclusion assay further provided direct evidence of increased cell death, reinforcing the conclusion that p‐cymene effectively reduces the viability of HCC cells (Chaudhry et al. 2024; Khalef et al. 2024; Stindlova et al. 2024).

To further elucidate the molecular mechanisms underlying p‐cymene's anticancer effects, we analyzed the expression of critical biomarkers involved in HCC pathogenesis. One of the most notable findings was the significant downregulation of HIF1A and VEGF, both of which play essential roles in angiogenesis and tumor adaptation to hypoxic conditions (Lee et al. 2021; Li et al. 2022; Paredes et al. 2021; Shah et al. 2021). The suppression of these markers suggests that p‐cymene may hinder tumor vascularization, thereby limiting nutrient and oxygen supply to cancer cells.

In addition to inhibiting angiogenesis, p‐cymene demonstrated a strong pro‐apoptotic effect by modulating key regulators of programmed cell death. Specifically, we observed a downregulation of the anti‐apoptotic protein BCL2 and an upregulation of CASP3, a key executor of apoptosis (Bhushan et al. 2024; Dou et al. 2024; Kaloni et al. 2023; Mohan et al. 2024; Qian et al. 2022). This balance shift toward apoptosis suggests that p‐cymene effectively triggers cell death in HCC cells.

The dysregulation of cell cycle regulators is a hallmark of cancer progression. Our study revealed that p‐cymene significantly downregulated CDK9 and JAK2, both of which are associated with cell cycle progression and survival signaling (Anshabo et al. 2021; D'costa et al. 2023; Huang et al. 2022; Mengie Ayele et al. 2022; Selvaraj 2023). CDK9 plays a crucial role in transcriptional elongation and cell proliferation, while JAK2 activation is involved in key oncogenic pathways, including the JAK/STAT signaling cascade. The observed suppression of these proteins suggests that p‐cymene may induce cell cycle arrest, further contributing to its anticancer properties. Moreover, tumor suppressor proteins play a crucial role in maintaining genomic integrity and preventing malignant transformation. Our study found a significant upregulation of P53 following p‐cymene treatment, reinforcing its role in promoting apoptosis and enhancing DNA damage responses (Al‐Arafat et al. 2024; Zhang et al. 2024). Since P53 is often inactivated in HCC, its restoration by p‐cymene suggests a potential mechanism by which this compound can suppress tumor growth and enhance cellular responses to stress (Meireles Da Costa et al. 2021).

STAT3 is another key oncogenic factor involved in promoting cell proliferation, immune evasion, and survival (Sohrabi et al. 2024; Zhang et al. 2022). Our findings revealed that p‐cymene treatment resulted in significant STAT3 inhibition, potentially restoring immune surveillance mechanisms that tumors often evade (Wong et al. 2022). This suggests that p‐cymene not only exerts direct cytotoxic effects on HCC cells but may also enhance immune‐mediated tumor suppression.

Moreover, the combination of p‐cymene with 5FU demonstrated a marked enhancement in cytotoxic efficacy compared to either agent alone, indicating a potential synergistic interaction that may contribute to overcoming resistance commonly observed with 5FU monotherapy. Resistance to 5FU in cancer cells is frequently associated with upregulation of thymidylate synthase, enhanced drug efflux, increased DNA repair capacity, and alterations in apoptotic pathways (Zou et al. 2025). Mechanistically, p‐cymene may modulate redox balance, inhibit survival pathways such as NF‐κB, and interfere with drug efflux or metabolism, all of which are implicated in chemoresistance. P‐cymene, with known anti‐inflammatory and anti‐oxidant properties (Singh and Barman 2021), may sensitize resistant cancer cells by modulating key apoptotic mechanisms (Silva et al. 2021). The observed reduction in cell viability in the combination treatment group suggests that p‐cymene could interfere with intracellular signaling pathways that confer resistance to chemotherapeutic agents. Notably, the combination treatment also resulted in significantly lower ALT and AST levels compared to 5FU alone, indicating that p‐cymene exerts a hepatoprotective effect. This dual action, “potentiating anticancer activity while mitigating hepatotoxicity,” suggests that p‐cymene may improve the therapeutic index of 5FU and allow for more effective treatment regimens in resistant cancers. These findings warrant further mechanistic studies to elucidate the precise molecular targets of p‐cymene and to validate its role in modulating chemoresistance.

## 5 Conclusion and Future Directions

Overall, our study highlights the multifaceted anticancer potential of p‐cymene in HCC. By targeting multiple pathways, including angiogenesis inhibition, apoptotic pathway activation, and cell cycle regulation, p‐cymene emerges as a promising candidate for HCC therapy. The compound's ability to modulate key oncogenic markers such as HIF1A, VEGF, BCL2, CASP3, CDK9, JAK2, P53, and STAT3 underscores its broad‐spectrum anticancer effects.

Future research should focus on further delineating the precise signaling mechanisms modulated by p‐cymene and assessing its therapeutic efficacy in in vivo models. Additionally, investigating potential synergistic interactions with existing chemotherapeutics could pave the way for developing effective combination therapies. The findings from this study provide a strong foundation for further exploration into the clinical applicability of p‐cymene, ultimately contributing to improved treatment strategies and patient outcomes in HCC.

## Author Contributions

**Nadia Anwar:** data curation (equal), formal analysis (equal), investigation (equal), methodology (equal), resources (equal), software (equal), validation (equal), visualization (equal), writing – original draft (equal). **Muhammad Nasir Hayat Malik:** conceptualization (equal), data curation (equal), formal analysis (equal), methodology (equal), project administration (equal), resources (equal), supervision (equal), validation (equal), visualization (equal), writing – review and editing (equal). **Muhammad Atif:** data curation (equal), formal analysis (equal), investigation (equal), methodology (equal), resources (equal), software (equal), validation (equal), visualization (equal), writing – review and editing (equal). **Abdullah R. Alanzi:** data curation (equal), formal analysis (equal), funding acquisition (equal), investigation (equal), methodology (equal), resources (equal), software (equal), validation (equal), visualization (equal), writing – review and editing (equal). **Hattan A. Alharbi:** data curation (equal), formal analysis (equal), investigation (equal), methodology (equal), resources (equal), software (equal), validation (equal), visualization (equal), writing – review and editing (equal). **Waqas Younis:** data curation (equal), formal analysis (equal), investigation (equal), methodology (equal), resources (equal), software (equal), validation (equal), visualization (equal), writing – review and editing (equal). **Munawar Abbas:** data curation (equal), formal analysis (equal), investigation (equal), methodology (equal), resources (equal), software (equal), validation (equal), visualization (equal), writing – review and editing (equal). **Gideon F. B. Solre:** conceptualization (equal), data curation (equal), formal analysis (equal), methodology (equal), project administration (equal), resources (equal), software (equal), validation (equal), writing – review and editing (equal).

## Conflicts of Interest

The authors declare no conflicts of interest.

## Supporting information


**Data S1:** fsn371108‐sup‐0001‐DataS1.docx.

## Data Availability

The datasets used and/or analyzed during the current study are available from the corresponding author on reasonable request.
