# Supplementary material for: p‐Cymene Targets Multiple Oncogenic Pathways in Hepatocellular Carcinoma: Insights From Network Pharmacology and In Vitro Studies
Source: Food Sci Nutr. 2025 Oct 16;13(10):e71108. doi: 10.1002/fsn3.71108 (PMC12531119; doi:10.1002/fsn3.71108)
Supplement: Supplementary file 1 — Data S1: fsn371108‐sup‐0001‐DataS1.docx. [file FSN3-13-e71108-s001.docx]

**Table S1:** **Chemical compounds and their structures**

| **Compound** | **PubChem CID** | **2D Structure** | **3D Structure** |
| --- | --- | --- | --- |
| p-cymene | 7463 | 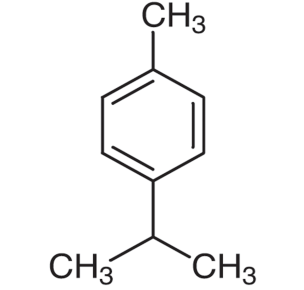 | 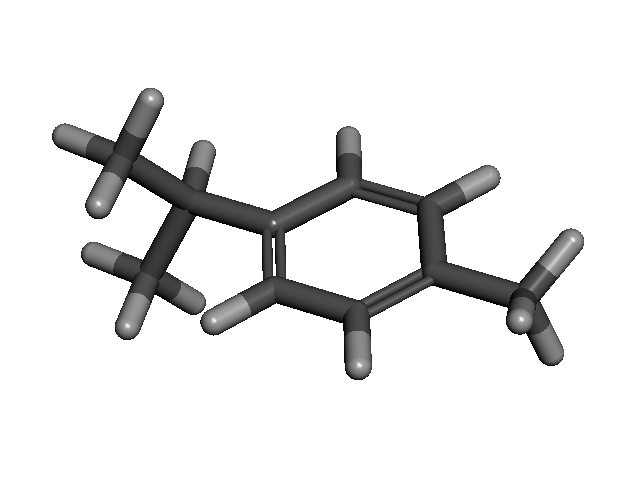 |
| 5FU | 3385 | 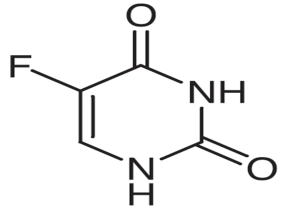 | 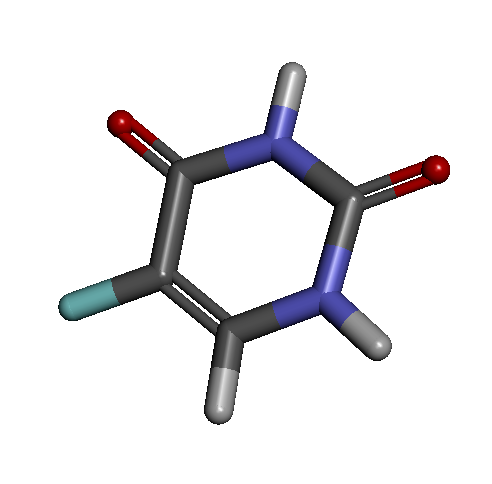 |

**Table S2: Predicted binding pocket residues of target proteins**

| **Proteins** | **Area (SA) Å^2^** | **Volume (SV) Å^3^** | **Binding pocket residues** |
| --- | --- | --- | --- |
| HIF1A (PDB ID: 1lqb) | 606.218 | 1192.204 | **Chain A**:(MET1 ASP2 VAL3 PHE4 SER64 GLN65 ALA67 ARG68 PRO69 GLN70)  **Chain B**:(ARG82 TYR83 THR88 GLU89 ILE90 PRO91 GLU92 PRO94)  **Chain C**:(ARG79 SER80 PRO81)  **Chain D**:(SER576 PHE577) |
| BCL2 (PDB ID: 2XA0) | 205.429 | 157.265 | **Chain A**:(GLN118 LEU119 HIS120 THR122 PRO123 PHE124 THR125 ARG129 THR132 VAL133 GLU136)  **Chain B:**(ARG127 PHE130 ALA131 THR132 VAL133 VAL134 GLU135 GLU136 PHE138 ARG139 TRP176 GLU179 TYR180 ARG183 HIS184)  **Chain C**:(LYS57 LYS58 LEU59 SER60 GLU61)  **Chain D**:(LYS58 LEU59 SER60) |
| CDK9 (PDB ID: 3BLR) | 1371.654 | 2664.636 | **Chain A**:(ILE25 GLY26 GLN27 THR29 PHE30 VAL33 ALA46 LYS48 LYS49 VAL50 LEU51 GLU55 PRO60 THR62 ALA63 GLU66 LEU70 VAL79 LEU101 PHE103 ASP104 PHE105 CYS106 ASP109 ASP149 LYS151 ALA153 ASN154 LEU156 ALA166 ASP167 PHE168 GLY169 LEU170) |
| JAK2 (PDB ID: 3JY9) | 949.120 | 755.957 | **Chain A**:(LEU855 GLY856 LYS857 GLY858 GLY861 SER862 VAL863 ALA880 LYS882 LEU884 GLN885 HIS886 SER887 GLU890 HIS891 ARG893 ASP894 PHE895 ARG897 GLU898 ILE901 LEU902 VAL911 LEU927 MET929 GLU930 TYR931 LEU932 PRO933 TYR934 GLY935 SER936 ARG938 ASP939 ARG975 ASP976 ARG980 ASN981 LEU983 GLY993 ASP994 PHE995 GLY996 LEU997 THR998 LYS999 VAL1000 PTR1008 LYS1009 VAL1010 LYS1011 GLU1012 PRO1013 GLY1014 GLU1015 SER1016 PRO1017 TYR1021) |
| VEGF (PDB ID: 3V2A) | 163.617 | 1141.824 | **Chain A**:(GLN132 HIS133 ARG164 TYR165 SER193 TYR194 ALA195 GLY196 MET197 ILE215 VAL216 VAL217 VAL218 LEU252 ASN253 VAL254 ASP276 LYS286 PHE288)  **Chain B**:(ASP34 PHE36 ILE46 PHE47 LYS48 PRO49 SER50 CYS51 GLN79 ILE80 MET81 ILE91) |
| MAPK4 (PDB ID: 4ZP5) | 4597.745 | 6600.539 | **Chain A**:(THR35 TYR36 GLY37 LYS54 MET56 GLU62 GLU65 ILE66 LEU68 GLU69 MET72 HIS151 ARG152 ASP153 ILE154 LYS155 ASN158 ASP171 PHE172 GLY173 ASN186 THR187 PHE188 ILE189 GLY190 THR191 PRO192 TYR193 TRP194 MET195 ALA196 VAL199 TYR210 SER218 CYS234 ASP235 MET236 HIS237 PRO238 MET239)  **Chain B** :(TYR36 GLY37 LYS54 MET56 ARG152 ASP153 ILE154 LYS155 ASN158 VAL170 GLN177 ASP179 ARG180 THR181 VAL182 GLY183 ARG184 ARG185 ASN186 THR187 PHE188 ILE189 GLY190 THR191 PRO192 TYR193 TRP194 MET195 ALA196 VAL199 ALA208 THR209 TYR210 SER218 HIS237 MET239) |
| P53 (PDB ID: 5O1H) | 2232.34 | 3165.54 | **Chain A**:(VAL97 PRO98 SER99 GLN100 LYS101 THR102 TYR103 ILE162 LYS164 SER166 MET169 LEU252 LEU264 ARG267)  **Chain B**:(PHE109 ALA138 THR140 LEU145 TRP146 VAL147 PRO151 VAL157 ASP184 SER185 ASP186 LEU188 ALA189 ARG196 VAL197 GLU198 GLY199 ASN200 LEU201 ARG202 ALA203 TYR205 VAL218 PRO219 CYS220 GLU221 PRO222 PRO223 GLU224 SER227 ASP228 CYS229 THR230 THR231 ILE232 HIS233 ASN235 MET237 LEU257) |
| STAT3 (PDB ID: 6NJS) | 89.624 | 652.55 | **Chain A**:(TRP243 GLN247 ALA250 CYS251 ILE252 GLY253 PRO256 ASN257 ILE258 LEU260 GLU324 ARG325 GLN326 CYS328 MET331 HIS332 PRO333 ASP334 ARG335 PRO336 LYS348 ILE467 CYS468 MET470 PRO471 TRP474 TRP510 GLN511 SER514 THR515 TRP562 ASP566 ASN567 ILE569 ASP570 LYS573) |
| CASP3 (PDB ID: 1NME) | 93.543 | 154.001 | **Chain A**:(GLY60 MET61 THR62 ARG64 SER120 HIS121 GLY122 GLU123 PHE128 GLN161 ALA162 CYS163 GLY165 THR166)  **Chain B**:(TYR204 SER205 TRP206 ARG207) |

**Table S3: Comprehensive overview of the predicted pharmacological activities of p-cymene**

**pa pi Predicted activity**

0,928 0,004 Ubiquinol-cytochrome-c reductase inhibitor

0,919 0,004 Mucomembranous protector

0,901 0,003 Glutamyl endopeptidase II inhibitor

0,898 0,001 Aryl-alcohol dehydrogenase inhibitor

0,890 0,006 Alkenylglycerophosphocholine hydrolase inhibitor

0,888 0,008 Testosterone 17beta-dehydrogenase (NADP+) inhibitor

0,881 0,002 Carminative

0,884 0,006 Antieczematic

0,875 0,004 2-Hydroxymuconate-semialdehyde hydrolase inhibitor

0,863 0,005 Membrane permeability inhibitor

0,869 0,015 Aspulvinone dimethylallyltransferase inhibitor

0,848 0,006 Alkylacetylglycerophosphatase inhibitor

0,846 0,004 Phosphatidylcholine-retinol O-acyltransferase inhibitor

0,861 0,021 CYP2C12 substrate

0,842 0,004 Venombin AB inhibitor

0,843 0,012 CYP2J substrate

0,833 0,005 5-O-(4-coumaroyl)-D-quinate 3'-monooxygenase inhibitor

0,846 0,018 Phobic disorders treatment

0,827 0,001 Benzaldehyde dehydrogenase (NAD+) inhibitor

0,828 0,010 Acylcarnitine hydrolase inhibitor

0,830 0,012 Sugar-phosphatase inhibitor

0,822 0,005 Omptin inhibitor

0,830 0,013 Antiseborrheic

0,831 0,015 Polyporopepsin inhibitor

0,814 0,004 All-trans-retinyl-palmitate hydrolase inhibitor

0,811 0,005 Alkane 1-monooxygenase inhibitor

0,815 0,011 HIF1A expression inhibitor

0,805 0,007 Ribulose-phosphate 3-epimerase inhibitor

0,804 0,009 Dehydro-L-gulonate decarboxylase inhibitor

0,796 0,004 Tpr proteinase (Porphyromonas gingivalis) inhibitor

0,796 0,005 Fatty-acyl-CoA synthase inhibitor

0,796 0,005 Fibrinolytic

0,797 0,006 Linoleate diol synthase inhibitor

0,795 0,007 Complement factor D inhibitor

0,801 0,017 Antineurotic

0,784 0,007 UDP-N-acetylglucosamine 4-epimerase inhibitor

0,779 0,008 Bisphosphoglycerate phosphatase inhibitor

0,784 0,015 Taurine dehydrogenase inhibitor

0,771 0,004 N-formylmethionyl-peptidase inhibitor

0,776 0,010 Fusarinine-C ornithinesterase inhibitor

0,782 0,016 CYP2J2 substrate

0,789 0,023 Chymosin inhibitor

0,789 0,023 Saccharopepsin inhibitor

0,789 0,023 Acrocylindropepsin inhibitor

0,775 0,014 Feruloyl esterase inhibitor

0,766 0,005 Electron-transferring-flavoprotein dehydrogenase inhibitor

0,763 0,006 Gluconate 5-dehydrogenase inhibitor

0,764 0,010 Glutathione thiolesterase inhibitor

0,758 0,007 Polyamine-transporting ATPase inhibitor

0,755 0,005 Chenodeoxycholoyltaurine hydrolase inhibitor

0,756 0,007 Vasoprotector

0,756 0,007 N-benzyloxycarbonylglycine hydrolase inhibitor

0,756 0,007 Limulus clotting factor B inhibitor

0,757 0,008 Nitrate reductase (cytochrome) inhibitor

0,758 0,010 NADPH-cytochrome-c2 reductase inhibitor

0,751 0,004 Insulin promoter

0,755 0,010 Oxidoreductase inhibitor

0,763 0,018 Nicotinic alpha6beta3beta4alpha5 receptor antagonist

0,750 0,006 CYP2B5 substrate

0,769 0,026 Chlordecone reductase inhibitor

0,750 0,010 IgA-specific serine endopeptidase inhibitor

0,744 0,004 Cyclohexyl-isocyanide hydratase inhibitor

0,748 0,012 Carboxypeptidase Taq inhibitor

0,739 0,004 Crotonoyl-[acyl-carrier-protein] hydratase inhibitor

0,737 0,005 Adenomatous polyposis treatment

0,735 0,005 tRNA-pseudouridine synthase I inhibitor

0,738 0,009 Acetylesterase inhibitor

0,739 0,011 Cl--transporting ATPase inhibitor

0,742 0,015 Arginine 2-monooxygenase inhibitor

0,731 0,004 Polyneuridine-aldehyde esterase inhibitor

0,731 0,005 Endopeptidase So inhibitor

0,737 0,011 Dimethylargininase inhibitor

0,729 0,005 Ferredoxin-NAD+ reductase inhibitor

0,729 0,005 Naphthalene 1,2-dioxygenase inhibitor

0,735 0,012 Arylsulfate sulfotransferase inhibitor

0,743 0,022 Pro-opiomelanocortin converting enzyme inhibitor

0,727 0,009 Methylamine-glutamate N-methyltransferase inhibitor

0,725 0,009 Pterin deaminase inhibitor

0,722 0,006 NADH kinase inhibitor

0,724 0,009 3-Hydroxybenzoate 6-monooxygenase inhibitor

0,722 0,008 Dextranase inhibitor

0,714 0,005 Arylmalonate decarboxylase inhibitor

0,714 0,006 Alkenylglycerophosphoethanolamine hydrolase inhibitor

0,715 0,007 Phenol O-methyltransferase inhibitor

0,712 0,005 Opheline kinase inhibitor

0,712 0,005 Taurocyamine kinase inhibitor

0,709 0,003 Plastoquinol-plastocyanin reductase inhibitor

0,709 0,004 H+-exporting ATPase inhibitor

0,719 0,015 Fragilysin inhibitor

0,745 0,047 Membrane integrity agonist

0,711 0,013 Membrane integrity antagonist

0,710 0,013 Phospholipid-translocating ATPase inhibitor

0,700 0,005 N-Acyl-D-aspartate deacylase inhibitor

0,701 0,007 Anesthetic general

0,705 0,012 27-Hydroxycholesterol 7alpha-monooxygenase inhibitor

0,698 0,009 Lysostaphin inhibitor

0,693 0,005 Gastrin inhibitor

0,718 0,031 Glycosylphosphatidylinositol phospholipase D inhibitor

0,690 0,005 Mannan endo-1,4-beta-mannosidase inhibitor

0,703 0,019 Glucan endo-1,6-beta-glucosidase inhibitor

0,691 0,007 Aminobutyraldehyde dehydrogenase inhibitor

0,690 0,006 Long-chain-aldehyde dehydrogenase inhibitor

0,695 0,012 Glucan endo-1,3-beta-D-glucosidase inhibitor

0,707 0,025 Nicotinic alpha2beta2 receptor antagonist

0,689 0,007 Cholestanetriol 26-monooxygenase inhibitor

0,700 0,020 Pseudolysin inhibitor

0,693 0,013 Phosphatidylserine decarboxylase inhibitor

0,694 0,016 Pullulanase inhibitor

0,690 0,013 Creatininase inhibitor

0,697 0,021 Protein-disulfide reductase (glutathione) inhibitor

0,685 0,009 Gamma-guanidinobutyraldehyde dehydrogenase inhibitor

0,697 0,025 Mucositis treatment

0,681 0,009 Antihypoxic

0,677 0,007 CYP2D16 substrate

0,678 0,009 Trimethylamine-oxide aldolase inhibitor

0,679 0,011 Cutinase inhibitor

0,677 0,009 Centromere associated protein inhibitor

0,680 0,014 Kidney function stimulant

0,670 0,005 Carbon-monoxide dehydrogenase inhibitor

0,672 0,007 Cathepsin T inhibitor

0,676 0,013 L-glutamate oxidase inhibitor

0,664 0,003 SULT1A3 substrate

0,673 0,012 Limulus clotting factor C inhibitor

0,668 0,009 Aspartate-ammonia ligase inhibitor

0,673 0,015 Thioredoxin inhibitor

0,663 0,005 2-Haloacid dehalogenase inhibitor

0,666 0,011 Urethanase inhibitor

0,676 0,021 Antidyskinetic

0,666 0,013 1,4-Lactonase inhibitor

0,661 0,012 Aspartate-phenylpyruvate transaminase inhibitor

0,659 0,011 CYP2A8 substrate

0,652 0,004 Plasmanylethanolamine desaturase inhibitor

0,666 0,019 Macrophage colony stimulating factor agonist

0,654 0,008 Tryptophanamidase inhibitor

0,661 0,015 Chloride peroxidase inhibitor

0,666 0,021 Phthalate 4,5-dioxygenase inhibitor

0,666 0,022 JAK2 expression inhibitor

0,651 0,008 CYP2F1 substrate

0,646 0,005 Albendazole monooxygenase inhibitor

0,670 0,032 NADPH peroxidase inhibitor

0,646 0,009 Prostaglandin-A1 DELTA-isomerase inhibitor

0,642 0,005 Endothelial growth factor antagonist

0,662 0,028 Sphinganine kinase inhibitor

0,654 0,021 Lysine 2,3-aminomutase inhibitor

0,637 0,004 Benzaldehyde dehydrogenase (NADP+) inhibitor

0,635 0,005 Glycolate dehydrogenase inhibitor

0,634 0,004 Acaricide

0,633 0,005 Antihelmintic (Nematodes)

0,632 0,004 N-hydroxy-2-acetamidofluorene reductase inhibitor

0,633 0,006 Peptidoglycan glycosyltransferase inhibitor

0,649 0,021 2-Hydroxyquinoline 8-monooxygenase inhibitor

0,646 0,019 CYP2B6 substrate

0,637 0,011 D-lactaldehyde dehydrogenase inhibitor

0,634 0,008 Aspergillopepsin I inhibitor

0,648 0,022 Exoribonuclease II inhibitor

0,652 0,028 G-protein-coupled receptor kinase inhibitor

0,652 0,028 Beta-adrenergic receptor kinase inhibitor

0,640 0,017 N-acetylneuraminate 7-O(or 9-O)-acetyltransferase inhibitor

0,637 0,014 Glucan 1,4-alpha-maltotriohydrolase inhibitor

0,625 0,004 Indoleacetaldoxime dehydratase inhibitor

0,625 0,005 Myeloblastin inhibitor

0,644 0,024 Antiinflammatory

0,623 0,005 2-Oxoaldehyde dehydrogenase (NADP+) inhibitor

0,634 0,016 CYP2C8 substrate

0,630 0,014 Acetylcholine neuromuscular blocking agent

0,619 0,005 CYP2C18 substrate

0,631 0,016 Glutamine-phenylpyruvate transaminase inhibitor

0,630 0,018 Formaldehyde transketolase inhibitor

0,623 0,011 Antiinfective

0,613 0,002 3,4-Dihydroxy-9,10-secoandrosta-1,3,5(10)-triene-9,17-dione 4,5-dioxygenase inhibitor

0,640 0,030 Phosphatase inhibitor

0,613 0,003 NF-E2-related factor 2 stimulant

0,616 0,006 N-(long-chain-acyl)ethanolamine deacylase inhibitor

0,618 0,009 Glutarate-semialdehyde dehydrogenase inhibitor

0,616 0,006 Gluconolactonase inhibitor

0,618 0,009 Allyl-alcohol dehydrogenase inhibitor

0,625 0,016 Calcium channel (voltage-sensitive) activator

0,642 0,034 Lysase inhibitor

0,623 0,016 4-Nitrophenol 2-monooxygenase inhibitor

0,625 0,020 ADP-thymidine kinase inhibitor

0,636 0,032 Glucose oxidase inhibitor

0,611 0,006 DELTA14-sterol reductase inhibitor

0,610 0,005 Acetylgalactosaminyl-O-glycosyl-glycoprotein beta-1,3-N-acetylglucosaminyltransferase inhibitor

0,616 0,012 CYP2A4 substrate

0,607 0,004 CYP2B11 substrate

0,607 0,005 Muscular dystrophy treatment

0,613 0,011 Erythropoiesis stimulant

0,619 0,019 Oxygen scavenger

0,608 0,008 Methanol dehydrogenase inhibitor

0,608 0,009 Glucan 1,4-alpha-maltotetraohydrolase inhibitor

0,608 0,012 Phosphoinositide 5-phosphatase inhibitor

0,605 0,010 N-acylmannosamine kinase inhibitor

0,619 0,024 Lipoprotein lipase inhibitor

0,618 0,024 CYP3A2 substrate

0,600 0,007 Mannitol-1-phosphatase inhibitor

0,604 0,011 (R)-Pantolactone dehydrogenase (flavin) inhibitor

0,599 0,006 6-Pyruvoyltetrahydropterin synthase inhibitor

0,608 0,016 Spermidine dehydrogenase inhibitor

0,609 0,017 Leukopoiesis stimulant

0,597 0,005 FMO1 substrate

0,600 0,009 Nitrite reductase (NO-forming) inhibitor

0,605 0,016 MAP kinase stimulant

0,606 0,018 Cholesterol antagonist

0,595 0,007 4-Hydroxyglutamate transaminase inhibitor

0,599 0,011 APOA1 expression enhancer

0,595 0,008 Antinociceptive

0,591 0,006 Ornithine cyclodeaminase inhibitor

0,600 0,016 CYP2A2 substrate

0,607 0,023 Platelet aggregation stimulant

0,592 0,009 Pyruvate decarboxylase inhibitor

0,597 0,014 IgA-specific metalloendopeptidase inhibitor

0,592 0,009 Opine dehydrogenase inhibitor

0,590 0,007 4-Chlorophenylacetate 3,4-dioxygenase inhibitor

0,601 0,019 CYP3A1 substrate

0,589 0,007 Arylesterase inhibitor

0,589 0,009 Di-trans,poly-cis-decaprenylcistransferase inhibitor

0,601 0,021 Biotinidase inhibitor

0,591 0,011 Alanine-tRNA ligase inhibitor

0,597 0,018 Fibrolase inhibitor

0,581 0,004 CYP2C10 substrate

0,592 0,015 S-alkylcysteine lyase inhibitor

0,592 0,015 Sulfite reductase inhibitor

0,588 0,012 Poly(beta-D-mannuronate) lyase inhibitor

0,590 0,014 Poly(alpha-L-guluronate) lyase inhibitor

0,581 0,006 CYP2D15 substrate

0,590 0,016 Corticosteroid side-chain-isomerase inhibitor

0,596 0,022 Hydrogen dehydrogenase inhibitor

0,579 0,007 Protein-Npi-phosphohistidine-sugar phosphotransferase inhibitor

0,593 0,022 Alopecia treatment

0,590 0,019 Histidine N-acetyltransferase inhibitor

0,577 0,007 2-Hydroxy-3-oxoadipate synthase inhibitor

0,579 0,009 Sulfite dehydrogenase inhibitor

0,584 0,014 Steroid N-acetylglucosaminyltransferase inhibitor

0,574 0,004 Trans-2-enoyl-CoA reductase (NAD+) inhibitor

0,574 0,004 Pyruvate dehydrogenase (cytochrome) inhibitor

0,587 0,019 (S)-6-hydroxynicotine oxidase inhibitor

0,573 0,005 Thiosulfate dehydrogenase inhibitor

0,575 0,007 Sorbitol-6-phosphate 2-dehydrogenase inhibitor

0,598 0,031 2-Dehydropantoate 2-reductase inhibitor

0,578 0,012 Na+-transporting two-sector ATPase inhibitor

0,573 0,009 Antiseptic

0,573 0,010 CYP2E1 inducer

0,579 0,016 Prolyl aminopeptidase inhibitor

0,565 0,005 Mannan endo-1,6-alpha-mannosidase inhibitor

0,587 0,027 Prostaglandin-E2 9-reductase inhibitor

0,583 0,023 Fructose 5-dehydrogenase inhibitor

0,571 0,012 Shikimate O-hydroxycinnamoyltransferase inhibitor

0,568 0,012 Octopamine antagonist

0,575 0,018 Methylumbelliferyl-acetate deacetylase inhibitor

0,575 0,019 Platelet adhesion inhibitor

0,560 0,004 Alcohol dehydrogenase [NAD(P)+] inhibitor

0,561 0,005 N-acetyl-gamma-glutamyl-phosphate reductase inhibitor

0,572 0,017 Antipruritic, allergic

0,561 0,007 Dolichyl-phosphatase inhibitor

0,561 0,007 Camphor 1,2-monooxygenase inhibitor

0,572 0,018 Amine dehydrogenase inhibitor

0,563 0,011 Glyoxylate oxidase inhibitor

0,587 0,035 Cytoprotectant

0,561 0,009 Gingipain K inhibitor

0,564 0,013 Isopenicillin-N epimerase inhibitor

0,553 0,003 CYP2C29 substrate

0,560 0,011 UGT1A6 substrate

0,555 0,006 BRAF expression inhibitor

0,554 0,006 Urease inhibitor

0,564 0,016 Peptide alpha-N-acetyltransferase inhibitor

0,566 0,018 Glyoxylate reductase inhibitor

0,564 0,018 Peptide-N4-(N-acetyl-beta-glucosaminyl)asparagine amidase inhibitor

0,572 0,028 (R)-6-hydroxynicotine oxidase inhibitor

0,552 0,008 Lactaldehyde reductase inhibitor

0,547 0,005 Flavin-containing monooxygenase inhibitor

0,547 0,005 Picornain 3C inhibitor

0,548 0,007 Indanol dehydrogenase inhibitor

0,546 0,005 2,4-Diaminopentanoate dehydrogenase inhibitor

0,546 0,005 3-Hydroxybutyryl-CoA dehydrogenase inhibitor

0,546 0,005 Lysine 6-dehydrogenase inhibitor

0,549 0,008 Clavaminate synthase inhibitor

0,549 0,010 Pancreatic elastase inhibitor

0,551 0,012 Cyclomaltodextrinase inhibitor

0,546 0,009 CDP-4-dehydro-6-deoxyglucose reductase inhibitor

0,552 0,016 Thymidylate 5'-phosphatase inhibitor

0,554 0,018 Cardiovascular analeptic

0,578 0,043 GST A substrate

0,540 0,005 Trans-pentaprenyltranstransferase inhibitor

0,562 0,028 Fucosterol-epoxide lyase inhibitor

0,543 0,010 Cyanoalanine nitrilase inhibitor

0,542 0,008 Snapalysin inhibitor

0,544 0,011 Alkylglycerone-phosphate synthase inhibitor

0,556 0,024 NAD(P)+-arginine ADP-ribosyltransferase inhibitor

0,564 0,033 Spasmolytic, urinary

0,555 0,023 Antipruritic

0,536 0,005 tRNA nucleotidyltransferase inhibitor

0,546 0,015 Xylan endo-1,3-beta-xylosidase inhibitor

0,543 0,013 CYP4A11 substrate

0,558 0,028 CYP2C9 substrate

0,540 0,011 Aryldialkylphosphatase inhibitor

0,537 0,009 Dementia treatment

0,538 0,011 Uroporphyrinogen-III synthase inhibitor

0,537 0,010 UGT1A4 substrate

0,549 0,023 CYP3A4 inducer

0,534 0,009 3-Hydroxy-4-oxoquinoline 2,4-dioxygenase inhibitor

0,555 0,031 Ovulation inhibitor

0,543 0,019 UGT1A9 substrate

0,527 0,005 Vanilloid 1 agonist

0,546 0,025 Neurotransmitter antagonist

0,534 0,013 Carboxypeptidase D inhibitor

0,551 0,031 Lipid metabolism regulator

0,532 0,012 Chitosanase inhibitor

0,543 0,024 Nucleoside oxidase (H2O2-forming) inhibitor

0,527 0,008 Aminomuconate-semialdehyde dehydrogenase inhibitor

0,529 0,010 Dimethylmaleate hydratase inhibitor

0,524 0,010 Membrane permeability enhancer

0,520 0,006 Pediculicide

0,544 0,031 Ecdysone 20-monooxygenase inhibitor

0,533 0,021 Hydroxylamine oxidase inhibitor

0,526 0,014 Anthranilate-CoA ligase inhibitor

0,521 0,009 CDP-diacylglycerol-glycerol-3-phosphate 3-phosphatidyltransferase inhibitor

0,516 0,004 Ferrochelatase inhibitor

0,519 0,008 3-Cyanoalanine hydratase inhibitor

0,585 0,075 CYP2H substrate

0,537 0,026 Mucinaminylserine mucinaminidase inhibitor

0,534 0,024 CYP3A inducer

0,515 0,007 CYP2A6 inhibitor

0,509 0,004 Alkylglycerophosphoethanolamine phosphodiesterase inhibitor

0,514 0,009 CYP7 inhibitor

0,515 0,012 Antipyretic

0,515 0,012 Aldehyde dehydrogenase (NADP+) inhibitor

0,511 0,009 Aminocarboxymuconate-semialdehyde decarboxylase inhibitor

0,508 0,008 Gly-X carboxypeptidase inhibitor

0,517 0,017 Sulfite oxidase inhibitor

0,501 0,002 Thermomycolin inhibitor

0,507 0,008 Cyclopentanone monooxygenase inhibitor

0,507 0,009 2-Haloacid dehalogenase (configuration-inverting) inhibitor

0,500 0,003 Alpha 1L adrenoreceptor agonist

0,507 0,010 Vitamin-K-epoxide reductase (warfarin-insensitive) inhibitor

0,501 0,005 Antiuremic

0,514 0,018 Phosphopantothenoylcysteine decarboxylase inhibitor

0,526 0,030 CYP2A1 substrate

0,518 0,023 Leukotriene-B4 20-monooxygenase inhibitor

0,507 0,012 Hydroxylamine reductase (NADH) inhibitor

0,503 0,010 Myosin ATPase inhibitor

0,502 0,009 3-Carboxyethylcatechol 2,3-dioxygenase inhibitor

0,517 0,025 Cytochrome P450 stimulant

0,527 0,035 Fibrinogen receptor antagonist

0,502 0,010 CYP2E1 inhibitor

0,501 0,010 DNA-3-methyladenine glycosylase I inhibitor

0,516 0,025 Mitochondrial processing peptidase inhibitor

0,497 0,006 Tryptophan transaminase inhibitor

0,508 0,017 Acylaminoacyl-peptidase inhibitor

0,534 0,043 Methylenetetrahydrofolate reductase (NADPH) inhibitor

0,510 0,021 Cis-1,2-dihydro-1,2-dihydroxynaphthalene dehydrogenase inhibitor

0,535 0,046 CYP2C8 inhibitor

0,501 0,012 Sphinganine-1-phosphate aldolase inhibitor

0,506 0,017 CYP2E1 substrate

0,492 0,004 Aspergillopepsin II inhibitor

0,520 0,034 CYP3A5 substrate

0,521 0,036 Peptidyl-dipeptidase Dcp inhibitor

0,505 0,020 Dolichyl-diphosphooligosaccharide-protein glycotransferase inhibitor

0,503 0,018 CYP2E substrate

0,508 0,025 Nicotine dehydrogenase inhibitor

0,494 0,012 Arylalkyl acylamidase inhibitor

0,505 0,023 CYP2C11 substrate

0,495 0,013 Imidazoline receptor agonist

0,491 0,010 Styrene-oxide isomerase inhibitor

0,508 0,028 Antisecretoric

0,509 0,030 5 Hydroxytryptamine uptake stimulant

0,498 0,019 Rubredoxin-NAD+ reductase inhibitor

0,488 0,010 N-Acyl-D-amino-acid deacylase inhibitor

0,505 0,028 MMP9 expression inhibitor

0,492 0,016 L-glucuronate reductase inhibitor

0,516 0,040 CYP2C substrate

0,506 0,029 Malate dehydrogenase (acceptor) inhibitor

0,501 0,026 S-formylglutathione hydrolase inhibitor

0,509 0,034 HMOX1 expression enhancer

0,477 0,002 SULT1A2 substrate

0,490 0,016 Neuropeptide Y2 antagonist

0,510 0,036 Respiratory analeptic

0,487 0,013 Taurine-2-oxoglutarate transaminase inhibitor

0,496 0,022 4-Hydroxyphenylacetate 3-monooxygenase inhibitor

0,478 0,004 3-Aminobutyryl-CoA ammonia-lyase inhibitor

0,488 0,015 CYP2C9 inducer

0,510 0,038 Peroxidase inhibitor

0,482 0,010 Loop diuretic

0,541 0,071 TP53 expression enhancer

0,481 0,012 Glutathione dehydrogenase (ascorbate) inhibitor

0,486 0,017 Aldehyde dehydrogenase (pyrroloquinoline-quinone) inhibitor

0,486 0,018 Nicotinate dehydrogenase inhibitor

0,514 0,045 Insulysin inhibitor

0,495 0,027 Apyrase inhibitor

0,484 0,016 Leucolysin inhibitor

0,482 0,015 D-alanine 2-hydroxymethyltransferase inhibitor

0,562 0,094 Nootropic

0,486 0,020 Atherosclerosis treatment

0,473 0,008 Quercetin 2,3-dioxygenase inhibitor

0,483 0,019 Hyponitrite reductase inhibitor

0,483 0,019 Fructan beta-fructosidase inhibitor

0,475 0,012 3-Hydroxybenzoate 4-monooxygenase inhibitor

0,500 0,038 Superoxide dismutase inhibitor

0,465 0,003 Raynaud's phenomenon treatment

0,492 0,030 CYP3A3 substrate

0,466 0,004 Sodium channel blocker class Ib

0,479 0,018 Undecaprenyldiphospho-muramoylpentapeptide beta-N-acetylglucosaminyltransferase inhibitor

0,465 0,005 Bile-salt sulfotransferase inhibitor

0,478 0,018 Mannose isomerase inhibitor

0,472 0,012 Linoleoyl-CoA desaturase inhibitor

0,497 0,038 Histamine release inhibitor

0,483 0,024 Threonine aldolase inhibitor

0,508 0,050 Preneoplastic conditions treatment

0,475 0,016 Tetrahydroxynaphthalene reductase inhibitor

0,476 0,019 Glycerol-3-phosphate dehydrogenase inhibitor

0,508 0,051 5 Hydroxytryptamine release stimulant

0,477 0,020 Malate oxidase inhibitor

0,486 0,030 Arylacetonitrilase inhibitor

0,470 0,013 Glycine dehydrogenase (decarboxylating) inhibitor

0,476 0,019 Glycerol-3-phosphate oxidase inhibitor

0,493 0,037 Sulfur reductase inhibitor

0,490 0,034 CYP2A6 substrate

0,485 0,030 Antiviral (Rhinovirus)

0,486 0,033 TNF expression inhibitor

0,468 0,016 Horrilysin inhibitor

0,470 0,018 EIF4E expression inhibitor

0,466 0,014 CYP19A1 expression inhibitor

0,464 0,012 6-Carboxyhexanoate-CoA ligase inhibitor

0,464 0,012 Triacetate-lactonase inhibitor

0,464 0,012 Homoaconitate hydratase inhibitor

0,464 0,012 Biotin-CoA ligase inhibitor

0,461 0,010 Bisphosphoglycerate mutase inhibitor

0,494 0,044 Pin1 inhibitor

0,455 0,005 CYP2C3 substrate

0,468 0,018 CYP2G1 substrate

0,480 0,030 Cyclohexanone monooxygenase inhibitor

0,464 0,015 Retinoic acid metabolism inhibitor

0,456 0,008 Trimethylamine dehydrogenase inhibitor

0,455 0,008 Indolepyruvate C-methyltransferase inhibitor

0,456 0,011 Sclerosant

0,462 0,017 Transketolase inhibitor

0,455 0,011 3-Demethylubiquinone-9 3-O-methyltransferase inhibitor

0,460 0,016 Glucuronate isomerase inhibitor

0,470 0,028 Simian immunodeficiency virus proteinase inhibitor

0,492 0,051 Aldehyde oxidase inhibitor

0,451 0,010 Sulfur dioxygenase inhibitor

0,468 0,028 N-hydroxyarylamine O-acetyltransferase inhibitor

0,451 0,012 Antiparkinsonian, rigidity relieving

0,442 0,003 Transforming growth factor beta 1 agonist

0,455 0,017 CYP2A5 substrate

0,472 0,035 CYP2C19 substrate

0,471 0,036 Analeptic

0,448 0,013 Alpha-N-acetylglucosaminidase inhibitor

0,440 0,007 Aldehyde dehydrogenase (NAD+) inhibitor

0,453 0,021 Bothrolysin inhibitor

0,440 0,008 Antinaupathic

0,459 0,027 N-methylhydantoinase (ATP-hydrolysing) inhibitor

0,443 0,012 Nardilysin inhibitor

0,435 0,005 Allantoinase inhibitor

0,458 0,028 Antihypercholesterolemic

0,475 0,046 Manganese peroxidase inhibitor

0,432 0,004 Tropinesterase inhibitor

0,458 0,030 Antiviral (Influenza)

0,446 0,018 Beta-carotene 15,15'-monooxygenase inhibitor

0,462 0,034 Renal tissue kallikrein inhibitor

0,457 0,030 Gonadotropin antagonist

0,437 0,010 Phosphoenolpyruvate-protein phosphotransferase inhibitor

0,436 0,009 CYP2B10 substrate

0,454 0,027 1-Acylglycerol-3-phosphate O-acyltransferase inhibitor

0,443 0,018 UGT2B4 substrate

0,437 0,013 Signal peptidase I inhibitor

0,455 0,030 GABA aminotransferase inhibitor

0,430 0,006 UGT2B9 substrate

0,448 0,024 Mediator release inhibitor

0,439 0,016 Oryzin inhibitor

0,441 0,018 2,3,4,5-Tetrahydropyridine-2,6-dicarboxylate N-succinyltransferase inhibitor

0,444 0,022 Phosphatidylglycerophosphatase inhibitor

0,449 0,028 Antimyopathies

0,430 0,010 Lombricine kinase inhibitor

0,430 0,011 Arylformamidase inhibitor

0,424 0,006 Glucan endo-1,3-alpha-glucosidase inhibitor

0,437 0,019 Phenylacetate-CoA ligase inhibitor

0,443 0,026 Adenylyl-sulfate reductase inhibitor

0,428 0,012 Aminoacylase inhibitor

0,449 0,033 4-Hydroxyproline epimerase inhibitor

0,438 0,022 Antiamyloidogenic

0,429 0,016 Morphine 6-dehydrogenase inhibitor

0,419 0,005 Retinal dehydrogenase inhibitor

0,447 0,035 AR expression inhibitor

0,417 0,004 Alpha-pinene-oxide decyclase inhibitor

0,432 0,021 Quinoprotein glucose dehydrogenase inhibitor

0,437 0,026 CYP2C19 inducer

0,438 0,027 UGT1A substrate

0,442 0,031 CYP2D2 inhibitor

0,424 0,012 Creatinine deaminase inhibitor

0,440 0,029 4-Methoxybenzoate monooxygenase (O-demethylating) inhibitor

0,441 0,029 CDK9/cyclin T1 inhibitor

0,432 0,021 Meprin B inhibitor

0,421 0,011 Farnesyltranstransferase inhibitor

0,432 0,023 Ethanolamine-phosphate cytidylyltransferase inhibitor

0,460 0,051 Cyclic AMP agonist

0,461 0,053 Thromboxane B2 antagonist

0,424 0,016 Serine-pyruvate transaminase inhibitor

0,415 0,007 Vascular dementia treatment

0,415 0,008 Antiinflammatory, ophthalmic

0,432 0,025 Antiparasitic

0,423 0,016 2,6-Dihydroxypyridine 3-monooxygenase inhibitor

0,432 0,025 Coccolysin inhibitor

0,420 0,014 Antihelmintic

0,447 0,042 CYP2A substrate

0,470 0,065 Antiviral (Picornavirus)

0,423 0,018 Oxidizing agent

0,412 0,007 Leucine dehydrogenase inhibitor

0,431 0,027 Lipid peroxidase inhibitor

0,407 0,004 Haloacetate dehalogenase inhibitor

0,407 0,004 Prepilin peptidase inhibitor

0,409 0,007 Alpha-glucuronidase inhibitor

0,436 0,034 CYP2B substrate

0,418 0,016 Anesthetic

0,454 0,052 Histamine release stimulant

0,427 0,025 Phosphatidylinositol diacylglycerol-lyase inhibitor

0,433 0,031 3-Phytase inhibitor

0,416 0,015 Dihydroxy-acid dehydratase inhibitor

0,433 0,033 Enteropeptidase inhibitor

0,410 0,010 Thiamine pyridinylase inhibitor

0,425 0,026 Salicylate 1-monooxygenase inhibitor

0,411 0,012 N-acetylneuraminate synthase inhibitor

0,408 0,009 Cholesterol oxidase inhibitor

0,446 0,047 Hypolipemic

0,446 0,047 5 Hydroxytryptamine release inhibitor

0,406 0,008 Diisopropyl-fluorophosphatase inhibitor

0,401 0,004 Transcription factor NF kappa A inhibitor

0,415 0,018 2-Oxoglutarate decarboxylase inhibitor

0,420 0,025 CYP4A substrate

0,406 0,012 Trans-1,2-dihydrobenzene-1,2-diol dehydrogenase inhibitor

0,407 0,013 Antipruritic, non-allergic

0,404 0,011 Penicillin amidase inhibitor

0,416 0,023 Succinate-semialdehyde dehydrogenase [NAD(P)+] inhibitor

0,415 0,022 Antiprotozoal (Amoeba)

0,398 0,005 Aldehyde ferredoxin oxidoreductase inhibitor

0,398 0,006 Nav1.5 sodium channel blocker

0,404 0,011 ICAM1 expression inhibitor

0,421 0,029 Carnitinamidase inhibitor

0,409 0,017 N-carbamoyl-L-amino-acid hydrolase inhibitor

0,407 0,017 Antiprotozoal (Coccidial)

0,465 0,076 Glyceryl-ether monooxygenase inhibitor

0,400 0,010 Serine 3-dehydrogenase inhibitor

0,399 0,010 UGT2B17 substrate

0,404 0,015 [acyl-carrier-protein] S-acetyltransferase inhibitor

0,402 0,015 Polygalacturonase inhibitor

0,446 0,059 Vasodilator, peripheral

0,403 0,017 Phosphoenolpyruvate mutase inhibitor

0,396 0,010 Aureolysin inhibitor

0,389 0,003 Tyrosine kinase stimulant

0,394 0,008 Valine decarboxylase inhibitor

0,423 0,037 X-methyl-His dipeptidase inhibitor

0,416 0,031 DNA-(apurinic or apyrimidinic site) lyase inhibitor

0,391 0,006 Insecticide

0,418 0,033 P-benzoquinone reductase (NADPH) inhibitor

0,410 0,028 Diamine N-acetyltransferase inhibitor

0,402 0,020 D-xylulose reductase inhibitor

0,428 0,047 Dermatologic

0,396 0,014 Aspartyl aminopeptidase inhibitor

0,414 0,033 Eye irritation, inactive

0,391 0,010 3-Hydroxyphenylacetate 6-hydroxylase inhibitor

0,389 0,008 Brachyurin inhibitor

0,410 0,030 Formate-dihydrofolate ligase inhibitor

0,389 0,010 Peptidylamidoglycolate lyase inhibitor

0,417 0,039 Antiulcerative

0,401 0,023 Glutamate-tRNA ligase inhibitor

0,417 0,040 Antipsoriatic

0,398 0,021 Acetylornithine deacetylase inhibitor

0,398 0,021 Methane monooxygenase inhibitor

0,409 0,033 Leukotriene-C4 synthase inhibitor

0,388 0,012 Maltose-transporting ATPase inhibitor

0,380 0,004 Antileprosy

0,401 0,026 Skin irritation, inactive

0,388 0,013 NAD+ synthase (glutamine-hydrolysing) inhibitor

0,395 0,020 N-acetyllactosamine synthase inhibitor

0,383 0,010 UGT2B1 substrate

0,402 0,029 Histidinol-phosphatase inhibitor

0,378 0,004 Urticaria treatment

0,385 0,012 Undecaprenyl-diphosphatase inhibitor

0,377 0,005 2-Oxoisovalerate dehydrogenase (acylating) inhibitor

0,415 0,044 Menopausal disorders treatment

0,396 0,025 Procollagen N-endopeptidase inhibitor

0,400 0,029 Hematopoietic inhibitor

0,376 0,006 Gingipain R inhibitor

0,392 0,022 FMO3 substrate

0,387 0,018 Hyaluronic acid agonist

0,383 0,013 Photosensitizer

0,391 0,022 GABA C receptor agonist

0,402 0,033 Cytochrome-b5 reductase inhibitor

0,383 0,015 Xaa-Pro dipeptidyl-peptidase inhibitor

0,389 0,021 Alpha-Methylacyl-CoA racemase inhibitor

0,389 0,020 Rhamnulose-1-phosphate aldolase inhibitor

0,385 0,018 Thiamine-triphosphatase inhibitor

0,400 0,032 Levanase inhibitor

0,373 0,006 Glyceraldehyde-3-phosphate dehydrogenase (phosphorylating) inhibitor

0,384 0,017 Pseudouridylate synthase inhibitor

0,385 0,018 Inulinase inhibitor

0,381 0,015 Pappalysin-1 inhibitor

0,369 0,004 SULT1A1 substrate

0,376 0,011 Pantoate 4-dehydrogenase inhibitor

0,392 0,027 Laccase inhibitor

0,384 0,021 Licheninase inhibitor

0,374 0,011 Glycerol-1-phosphatase inhibitor

0,384 0,021 2,4-Dichlorophenol 6-monooxygenase inhibitor

0,379 0,017 D-threo-aldose 1-dehydrogenase inhibitor

0,386 0,024 Dipeptidase E inhibitor

0,377 0,016 Steroid synthesis inhibitor

0,394 0,033 Antituberculosic

0,401 0,041 Calcium regulator

0,375 0,016 Adrenaline release stimulant

0,375 0,016 Tryptophan 2,3-dioxygenase inhibitor

0,370 0,012 Orotate reductase (NADH) inhibitor

0,395 0,037 Monodehydroascorbate reductase (NADH) inhibitor

0,398 0,040 Alcohol dehydrogenase (acceptor) inhibitor

0,396 0,038 Thiol oxidase inhibitor

0,371 0,013 Aspartate-tRNA ligase inhibitor

0,381 0,024 4-Hydroxybenzoate nonaprenyltransferase inhibitor

0,406 0,049 RNA-directed RNA polymerase inhibitor

0,400 0,043 Leukopoiesis inhibitor

0,388 0,032 Phosphatidate phosphatase inhibitor

0,360 0,004 Farnesoid X receptor antagonist

0,368 0,012 CYP4A2 substrate

0,377 0,021 Mitochondrial intermediate peptidase inhibitor

0,379 0,024 Glycerol 2-dehydrogenase (NADP+) inhibitor

0,380 0,025 Gamma-D-Glutamyl-meso-diaminopimelate peptidase inhibitor

0,365 0,010 Cycloartenol synthase inhibitor

0,378 0,023 Phenylalanine(histidine) transaminase inhibitor

0,394 0,040 NADPH-ferrihemoprotein reductase inhibitor

0,370 0,017 Acetate kinase inhibitor

0,368 0,015 Aspartoacylase inhibitor

0,364 0,012 CYP2B2 substrate

0,458 0,106 Acute neurologic disorders treatment

0,374 0,022 Glycine amidinotransferase inhibitor

0,364 0,012 Sulfotransferase substrate

0,366 0,015 Dopamine release stimulant

0,390 0,038 4-Coumarate-CoA ligase inhibitor

0,372 0,021 TRPA1 agonist

0,368 0,019 Prunasin beta-glucosidase inhibitor

0,429 0,080 CYP3A4 substrate

0,353 0,003 Vascular adhesion protein 1 inhibitor

0,384 0,036 Phosphatidylcholine-sterol O-acyltransferase inhibitor

0,366 0,017 Antiparkinsonian, tremor relieving

0,370 0,021 L-threonine 3-dehydrogenase inhibitor

0,363 0,014 Cystic fibrosis treatment

0,391 0,042 Myc inhibitor

0,396 0,048 CYP1A2 substrate

0,398 0,051 Reductant

0,386 0,039 Pitrilysin inhibitor

0,363 0,017 Fumarate reductase (NADH) inhibitor

0,367 0,022 UGT1A1 substrate

0,359 0,013 Lipoprotein disorders treatment

0,373 0,027 Mandelate 4-monooxygenase inhibitor

0,371 0,025 Non-steroidal antiinflammatory agent

0,365 0,019 Arylsulfatase inhibitor

0,361 0,016 Palmitoyl-CoA hydrolase inhibitor

0,361 0,016 Acyl-CoA hydrolase inhibitor

0,372 0,027 Magnesium-protoporphyrin IX monomethyl ester (oxidative) cyclase inhibitor

0,353 0,008 Deoxyribose-phosphate aldolase inhibitor

0,366 0,022 Phospholipase C inhibitor

0,353 0,009 Alkene monooxygenase inhibitor

0,365 0,021 UGT2B substrate

0,357 0,014 Sterol 3-beta-glucosyltransferase inhibitor

0,385 0,042 Aspergillus nuclease S1 inhibitor

0,361 0,018 CYP1A2 inducer

0,346 0,004 Nitric oxide scavenger

0,380 0,038 Antiviral (Adenovirus)

0,368 0,027 Flavin-containing monooxygenase substrate

0,356 0,015 3-Chloro-D-alanine dehydrochlorinase inhibitor

0,349 0,008 Pancreatic endopeptidase E inhibitor

0,345 0,004 Choloylglycine hydrolase inhibitor

0,355 0,015 3-Oxoadipate enol-lactonase inhibitor

0,354 0,014 Allantoate deiminase inhibitor

0,349 0,009 Antihyperlipoproteinemic

0,356 0,016 Hydroxymethylbilane synthase inhibitor

0,383 0,044 Antimycobacterial

0,381 0,043 H+-transporting two-sector ATPase inhibitor

0,389 0,052 CYP1A substrate

0,394 0,056 Caspase 8 stimulant

0,360 0,023 CYP2A3 substrate

0,353 0,016 Antiperistaltic

0,351 0,015 N-Carbamoyl-D-amino acid hydrolase inhibitor

0,369 0,034 Opioid kappa 3 receptor antagonist

0,361 0,026 Peroxidase substrate

0,355 0,022 Gallate decarboxylase inhibitor

0,402 0,069 Alcohol O-acetyltransferase inhibitor

0,340 0,007 ATPase inhibitor

0,351 0,018 Hydroxylamine reductase inhibitor

0,357 0,024 Ubiquitin thiolesterase inhibitor

0,340 0,008 Anthrax lethal factor inhibitor

0,346 0,014 Antianorexic

0,350 0,019 Lysyl oxidase inhibitor

0,336 0,004 Viral entry inhibitor

0,356 0,025 2,3-Dihydroxyindole 2,3-dioxygenase inhibitor

0,361 0,031 Yeast ribonuclease inhibitor

0,342 0,013 Glycerone-phosphate O-acyltransferase inhibitor

0,364 0,035 Antiinflammatory, intestinal

0,344 0,016 2,5-Dihydroxypyridine 5,6-dioxygenase inhibitor

0,365 0,037 4-Nitrophenylphosphatase inhibitor

0,333 0,006 Quinoline-4-carboxylate 2-oxidoreductase inhibitor

0,370 0,043 4-Hydroxymandelate oxidase inhibitor

0,333 0,006 GABA B receptor agonist

0,372 0,045 Protein-glutamate methylesterase inhibitor

0,346 0,020 Glycopeptide alpha-N-acetylgalactosaminidase inhibitor

0,343 0,017 UGT2B15 substrate

0,331 0,005 CYP2B18 substrate

0,363 0,037 Para amino benzoic acid antagonist

0,409 0,083 CYP3A substrate

0,347 0,022 UGT2B7 substrate

0,354 0,030 Chaperonin ATPase inhibitor

0,371 0,047 RELA expression inhibitor

0,354 0,030 Gamma-glutamyltransferase inhibitor

0,376 0,052 Radioprotector

0,333 0,010 Granzyme A inhibitor

0,339 0,016 Amyloid beta precursor protein antagonist

0,348 0,025 Galactolipase inhibitor

0,350 0,027 4-Chlorobenzoyl-CoA dehalogenase inhibitor

0,345 0,023 Rhodotorulapepsin inhibitor

0,327 0,006 Aldehyde dehydrogenase [NAD(P)+] inhibitor

0,356 0,036 Cancer associated disorders treatment

0,342 0,022 2-Acylglycerol O-acyltransferase inhibitor

0,340 0,021 UGT1A3 substrate

0,326 0,007 Retinyl-palmitate esterase inhibitor

0,350 0,030 CYP2C6 substrate

0,365 0,047 Nitrite reductase [NAD(P)H] inhibitor

0,330 0,011 Acetylenecarboxylate hydratase inhibitor

0,326 0,008 Vanilloid agonist

0,359 0,041 Venom exonuclease inhibitor

0,385 0,069 Immunosuppressant

0,331 0,015 (S)-3-amino-2-methylpropionate transaminase inhibitor

0,327 0,011 Aldehyde dehydrogenase 2 substrate

0,346 0,032 GST M substrate

0,344 0,030 Catalase inhibitor

0,319 0,005 Carboxylate reductase inhibitor

0,353 0,040 UDP-glucuronosyltransferase substrate

0,362 0,049 Cell adhesion molecule inhibitor

0,360 0,047 Intermittent claudication treatment

0,335 0,023 Nitrate reductase inhibitor

0,318 0,006 Retinol O-fatty-acyltransferase inhibitor

0,329 0,017 Paraoxonase substrate

0,327 0,016 Phenylpyruvate decarboxylase inhibitor

0,335 0,025 UGT2B28 substrate

0,356 0,045 Cardioprotectant

0,368 0,058 Antifungal

0,334 0,024 Guanidinoacetase inhibitor

0,317 0,007 Tryptophanyl aminopeptidase inhibitor

0,333 0,024 Inorganic diphosphatase inhibitor

0,332 0,025 Alpha-amylase inhibitor

0,326 0,020 Lactate 2-monooxygenase inhibitor

0,367 0,061 Spasmolytic

0,330 0,024 Subtilisin inhibitor

0,330 0,024 15-Hydroxyprostaglandin-D dehydrogenase (NADP+) inhibitor

0,354 0,049 Botulin neurotoxin A light chain inhibitor

0,315 0,010 Oligopeptidase B inhibitor

0,362 0,057 Chitinase inhibitor

0,313 0,007 Valine dehydrogenase (NADP+) inhibitor

0,325 0,020 Methylaspartate ammonia-lyase inhibitor

0,347 0,043 Acidifying agent non gastric

0,313 0,009 GST T1-1 substrate

0,313 0,009 GST T substrate

0,359 0,055 Antiviral (Herpes)

0,321 0,017 Monophenol monooxygenase inhibitor

0,320 0,016 Sulfate adenylyltransferase (ADP) inhibitor

0,310 0,006 Catechol 1,2-dioxygenase inhibitor

0,321 0,018 Cyclamate sulfohydrolase inhibitor

0,308 0,004 DNA-3-methyladenine glycosylase II inhibitor

0,320 0,017 3-Isopropylmalate dehydratase inhibitor

0,315 0,012 CYP4B substrate

0,367 0,065 Caspase 3 stimulant

0,315 0,013 Creatinase inhibitor

0,334 0,032 NOS2 expression inhibitor

0,347 0,045 Skeletal muscle relaxant

0,335 0,034 Ferredoxin hydrogenase inhibitor

0,316 0,015 Anthranilate 3-monooxygenase (deaminating) inhibitor

0,329 0,028 Glutaminase inhibitor

0,335 0,035 Tyrosine 3 hydroxylase inhibitor

0,317 0,018 CNS active muscle relaxant

0,312 0,013 2,2-Dialkylglycine decarboxylase (pyruvate) inhibitor

0,382 0,083 Heat shock protein 27 antagonist

0,311 0,011 Cholate-CoA ligase inhibitor

0,310 0,011 Pectin lyase inhibitor

0,340 0,041 Spasmolytic, Papaverin-like

0,356 0,057 Sigma receptor agonist

0,373 0,074 Diabetic neuropathy treatment

0,310 0,011 Protein-S-isoprenylcysteine O-methyltransferase inhibitor

0,356 0,057 1-Alkylglycerophosphocholine O-acetyltransferase inhibitor

0,338 0,040 Antineurogenic pain

0,334 0,035 Envelysin inhibitor

0,312 0,013 Ligase inhibitor

0,316 0,018 Gametolysin inhibitor

0,320 0,022 Age-related macular degeneration treatment

0,377 0,079 Nucleotide metabolism regulator

0,317 0,020 UGT1A8 substrate

0,308 0,011 D-cysteine desulfhydrase inhibitor

0,344 0,047 Antialcoholic

0,321 0,024 Arylamine N-acetyltransferase inhibitor

0,318 0,022 Retinol dehydrogenase inhibitor

0,301 0,006 Diabetic nephropathy treatment

0,307 0,012 Pyridoxine 4-oxidase inhibitor

0,314 0,018 Ferredoxin-nitrite reductase inhibitor

0,319 0,024 Peptide-tryptophan 2,3-dioxygenase inhibitor

0,303 0,008 Fructose-2,6-bisphosphate 6-phosphatase inhibitor

0,351 0,056 Choline-phosphate cytidylyltransferase inhibitor

0,320 0,025 1,2-alpha-L-fucosidase inhibitor

0,310 0,015 Benzoate 4-monooxygenase inhibitor

0,329 0,035 DNA ligase (ATP) inhibitor

0,302 0,008 Glucuronolactone reductase inhibitor

0,308 0,014 Peptidyl-glycinamidase inhibitor

0,322 0,028 Guanidinoacetate N-methyltransferase inhibitor

0,300 0,007 Renilla-luciferin 2-monooxygenase inhibitor

0,323 0,031 Bontoxilysin inhibitor

0,334 0,043 Monoamine uptake inhibitor

0,303 0,012 Acyl-lysine deacylase inhibitor

0,313 0,023 (S)-3-hydroxyacid ester dehydrogenase inhibitor

0,308 0,019 Hydroxysteroid dehydrogenase inhibitor

0,297 0,008 Pyridoxine 5-dehydrogenase inhibitor

0,297 0,009 Chloramphenicol O-acetyltransferase inhibitor

0,309 0,022 Phosphatidate cytidylyltransferase inhibitor

0,327 0,040 Antitoxic

0,314 0,027 Glucan 1,4-beta-glucosidase inhibitor

0,302 0,015 Uric acid excretion stimulant

0,319 0,033 D-amino-acid dehydrogenase inhibitor

0,317 0,031 Aminopeptidase Y inhibitor

0,305 0,019 Alpha-N-arabinofuranosidase inhibitor

0,302 0,017 Glycerol dehydrogenase (NADP+) inhibitor

0,295 0,010 HIV-2 reverse transcriptase inhibitor

0,303 0,018 Formaldehyde dehydrogenase inhibitor

0,299 0,014 Aromatic-hydroxylamine O-acetyltransferase inhibitor

0,305 0,020 Endo-1,3(4)-beta-glucanase inhibitor

0,306 0,022 Galactokinase inhibitor

0,313 0,029 N-acetyllactosaminide beta-1,3-N-acetylglucosaminyltransferase inhibitor

0,306 0,023 Tauropine dehydrogenase inhibitor

0,306 0,023 3-Methylbutanal reductase inhibitor

0,301 0,020 (R,R)-butanediol dehydrogenase inhibitor

0,293 0,011 D-2-hydroxy-acid dehydrogenase inhibitor

0,297 0,016 Thermitase inhibitor

0,294 0,013 D-lactate dehydrogenase (cytochrome) inhibitor

0,334 0,053 Immunomodulator

0,300 0,019 Shikimate 5-dehydrogenase inhibitor

0,306 0,026 Antiprotozoal (Trichomonas)

0,294 0,014 Potassium channel intermediate-conductance Ca-activated activator

0,294 0,014 K(Ca) 3.1 channel activator

0,295 0,015 Neurotrophic factor enhancer

0,297 0,019 Anesthetic local

0,285 0,007 Orotate reductase (NADPH) inhibitor

0,305 0,028 Apoptosis antagonist

0,288 0,011 Methyltransferase substrate

0,304 0,027 Retinoprotector

0,285 0,008 Ribitol 2-dehydrogenase inhibitor

0,283 0,006 Glucosamine-1-phosphate N-acetyltransferase inhibitor

0,297 0,021 Aryl hydrocarbon receptor agonist

0,327 0,051 Hepatoprotectant

0,286 0,010 Cytochrome-c3 hydrogenase inhibitor

0,303 0,027 Vomilenine glucosyltransferase inhibitor

0,308 0,033 Glutaminyl-peptide cyclotransferase inhibitor

0,305 0,030 Clostripain inhibitor

0,293 0,018 Phloroglucinol reductase inhibitor

0,282 0,009 Triose-phosphate isomerase inhibitor

0,291 0,018 Magnesium-protoporphyrin IX methyltransferase inhibitor

0,295 0,023 Metallocarboxypeptidase D inhibitor

0,286 0,014 Hydroxymethylglutaryl-CoA lyase inhibitor

0,285 0,013 3-Ketovalidoxylamine C-N-lyase inhibitor

0,284 0,012 Selenocysteine lyase inhibitor

0,284 0,012 Picornain 2A inhibitor

0,295 0,024 N6-methyl-lysine oxidase inhibitor

0,295 0,024 Acyl-CoA oxidase inhibitor

0,304 0,033 Choleretic

0,291 0,022 Aldosterone antagonist

0,312 0,043 Wound healing agent

0,295 0,026 CYP1A1 inhibitor

0,299 0,030 Antiviral (CMV)

0,292 0,023 UGT1A10 substrate

0,278 0,010 Phenylacetaldehyde dehydrogenase inhibitor

0,280 0,012 CYP4B1 substrate

0,273 0,006 Aspartate racemase inhibitor

0,285 0,019 Allantoin racemase inhibitor

0,271 0,004 Pregnane X receptor agonist

0,275 0,008 Stromelysin 2 inhibitor

0,294 0,028 Oxytocic

0,271 0,004 Haloalkane dehalogenase inhibitor

0,290 0,024 Isopenicillin-N synthase inhibitor

0,296 0,030 2-Enoate reductase inhibitor

0,319 0,053 CYP2C19 inhibitor

0,301 0,035 Alpha-1,6-mannosyl-glycoprotein 4-beta-N-acetylglucosaminyltransferase inhibitor

0,290 0,024 Aryl sulfotransferase inhibitor

0,330 0,064 CYP3A7 substrate

0,293 0,028 Cell wall biosynthesis inhibitor

0,277 0,011 Cysteine desulfurase inhibitor

0,287 0,022 Choline-sulfatase inhibitor

0,286 0,022 Interleukin 6 antagonist

0,286 0,022 N-acetylneuraminate 4-O-acetyltransferase inhibitor

0,300 0,036 Chemoprotective

0,268 0,005 Antithyroid

0,282 0,019 L-iduronidase inhibitor

0,276 0,013 Carboxymethylenebutenolidase inhibitor

0,266 0,004 Glycine receptor agonist

0,270 0,007 Guanidinodeoxy-scyllo-inositol-4-phosphatase inhibitor

0,297 0,035 Sodium channel blocker

0,298 0,036 Acetylserotonin O-methyltransferase inhibitor

0,274 0,013 Estradiol 17alpha-dehydrogenase inhibitor

0,280 0,018 Guanosine-3',5'-bis(diphosphate) 3'-diphosphatase inhibitor

0,284 0,022 Antifibrinolytic

0,275 0,014 Saccharopine dehydrogenase (NADP+, L-glutamate-forming) inhibitor

0,268 0,008 CYP1A3 substrate

0,287 0,026 Chloride channel activator

0,280 0,019 Thiamine-phosphate kinase inhibitor

0,297 0,037 Lactose synthase inhibitor

0,284 0,025 CYP2D1 substrate

0,292 0,033 Malate dehydrogenase inhibitor

0,311 0,053 CYP1A1 substrate

0,301 0,042 Calcium channel activator

0,291 0,032 Galactose oxidase inhibitor

0,273 0,014 Acetoacetate decarboxylase inhibitor

0,269 0,011 Glycerol dehydratase inhibitor

0,273 0,015 L-galactonolactone oxidase inhibitor

0,336 0,079 Bilirubin oxidase inhibitor

0,280 0,024 N-methyl-2-oxoglutaramate hydrolase inhibitor

0,266 0,010 Aconitate decarboxylase inhibitor

0,267 0,011 Protein synthesis stimulant

0,267 0,011 Choline dehydrogenase inhibitor

0,261 0,006 Vanillyl-alcohol oxidase inhibitor

0,260 0,005 4-Carboxymethyl-4-methylbutenolide mutase inhibitor

0,265 0,011 Protein kinase stimulant

0,285 0,031 Glyoxylate reductase (NADP+) inhibitor

0,262 0,008 Scytalone dehydratase inhibitor

0,263 0,009 Nav1.1 sodium channel blocker

0,298 0,044 Calpain inhibitor

0,268 0,015 Protein-glucosylgalactosylhydroxylysine glucosidase inhibitor

0,272 0,019 Carnitine 3-dehydrogenase inhibitor

0,300 0,047 5 Hydroxytryptamine 2B agonist

0,318 0,065 Diuretic inhibitor

0,260 0,008 5-Oxoprolinase (ATP-hydrolysing) inhibitor

0,257 0,006 Acetolactate synthase inhibitor

0,255 0,004 CYP3C substrate

0,272 0,022 Hippurate hydrolase inhibitor

0,268 0,018 Coproporphyrinogen oxidase inhibitor

0,273 0,023 Phenylalanine racemase (ATP-hydrolysing) inhibitor

0,255 0,004 Acid-sensing ion channel blocker

0,274 0,024 Inositol-polyphosphate 5-phosphatase inhibitor

0,276 0,026 Cyclooxygenase substrate

0,274 0,024 Rhizopuspepsin inhibitor

0,264 0,014 Antihematotoxic

0,266 0,017 Glycerol-3-phosphate O-acyltransferase inhibitor

0,276 0,027 Antihelmintic (Fasciola)

0,271 0,023 2-Dehydropantoate aldolase inhibitor

0,342 0,094 Anticonvulsant

0,261 0,014 Threonine ammonia-lyase inhibitor

0,257 0,010 (R)-limonene 6-monooxygenase inhibitor

0,278 0,031 Serratia marcescens nuclease inhibitor

0,291 0,044 Dactylysin inhibitor

0,250 0,003 DELTA24-sterol reductase inhibitor

0,260 0,014 Allophanate hydrolase inhibitor

0,280 0,034 Cellulose 1,4-beta-cellobiosidase inhibitor

0,301 0,055 Inotropic

0,266 0,020 Urate-ribonucleotide phosphorylase inhibitor

0,264 0,018 Deoxyribonuclease I inhibitor

0,333 0,087 CYP2D substrate

0,323 0,078 Antiprotozoal (Trypanosoma)

0,269 0,024 Gamma-butyrobetaine dioxygenase inhibitor

0,251 0,007 6-Methylsalicylate decarboxylase inhibitor

0,270 0,026 Calcium-sensing receptor agonist

0,253 0,009 CYP2B4 substrate

0,299 0,055 Opioid dependency treatment

0,257 0,013 NAD(P)+ transhydrogenase (B-specific) inhibitor

0,282 0,038 Anti-Helicobacter pylori

0,272 0,028 Cerebroside-sulfatase inhibitor

0,252 0,009 Hepatocyte nuclear factor antagonist

0,252 0,009 Hepatocyte nuclear factor 4 alpha antagonist

0,283 0,040 Guanidinoacetate kinase inhibitor

0,257 0,015 Tyrosine-ester sulfotransferase inhibitor

0,271 0,028 Proteasome endopeptidase complex inhibitor

0,276 0,034 MO15-related protein kinase Pfmrk inhibitor

0,319 0,077 Prion diseases treatment

0,258 0,016 Mevalonate kinase inhibitor

0,267 0,026 Gentisate 1,2-dioxygenase inhibitor

0,255 0,014 Alpha-N-acetylgalactosaminidase inhibitor

0,269 0,028 Cholesterol synthesis inhibitor

0,253 0,013 Cytochrome-b5 reductase substrate

0,268 0,027 Lipoxygenase substrate

0,260 0,020 Malate-CoA ligase inhibitor

0,271 0,031 Tripeptide aminopeptidase inhibitor

0,261 0,022 N-(5-amino-5-carboxypentanoyl)-L-cysteinyl-D-valine synthase inhibitor

0,275 0,035 Cyclopropane-fatty-acyl-phospholipid synthase inhibitor

0,247 0,008 D-stereospecific aminopeptidase inhibitor

0,247 0,008 Coagulant

0,274 0,036 O-aminophenol oxidase inhibitor

0,383 0,145 Proteasome ATPase inhibitor

0,266 0,029 Glycogen (starch) synthase inhibitor

0,261 0,024 Beta-mannosidase inhibitor

0,242 0,005 Cyclooxygenase 3 inhibitor

0,255 0,017 Carnitine dehydratase inhibitor

0,278 0,041 3C-like protease (Human coronavirus) inhibitor

0,294 0,058 Endopeptidase La inhibitor

0,257 0,021 Iron-cytochrome-c reductase inhibitor

0,267 0,031 Peptidyl-Lys metalloendopeptidase inhibitor

0,262 0,026 Acylglycerone-phosphate reductase inhibitor

0,263 0,027 Atrolysin A inhibitor

0,250 0,014 Sodium channel (voltage-gated) blocker

0,267 0,031 Glutamate 5-kinase inhibitor

0,273 0,038 Dynein ATPase inhibitor

0,278 0,043 Tripeptidyl-peptidase I inhibitor

0,250 0,015 Neuropsin inhibitor

0,271 0,036 GABA receptor agonist

0,303 0,069 Pyroglutamyl-peptidase II inhibitor

0,252 0,018 Isovaleryl-CoA dehydrogenase inhibitor

0,265 0,031 Expectorant

0,238 0,004 Bombesin 1 receptor antagonist

0,258 0,024 Tryptophan dimethylallyltransferase inhibitor

0,257 0,024 Gout treatment

0,273 0,041 Acrosin inhibitor

0,263 0,031 CYP2B1 substrate

0,253 0,021 Phosphoinositide phospholipase C inhibitor

0,255 0,024 Laxative

0,247 0,017 Deacetoxycephalosporin-C synthase inhibitor

0,240 0,010 D-arabinonolactone oxidase inhibitor

0,258 0,027 Glucose 1-dehydrogenase inhibitor

0,270 0,040 Hemostatic

0,320 0,090 Rhinitis treatment

0,250 0,020 Quisqualate antagonist

0,249 0,020 Methionine decarboxylase inhibitor

0,269 0,039 Saluretic

0,247 0,018 Glucarate dehydratase inhibitor

0,242 0,013 Purinergic P2X3 antagonist

0,249 0,020 Galactose 1-dehydrogenase (NADP+) inhibitor

0,249 0,020 Beta-alanine-pyruvate transaminase inhibitor

0,249 0,022 Lysyl endopeptidase inhibitor

0,259 0,032 Peptidyl-dipeptidase B inhibitor

0,258 0,030 Antimycoplasmal

0,250 0,023 Ferredoxin-NADP+ reductase inhibitor

0,272 0,046 CYP1A inducer

0,326 0,099 Vasodilator, coronary

0,247 0,020 Isoquinoline 1-oxidoreductase inhibitor

0,257 0,030 CYP4F2 substrate

0,245 0,019 Catenin beta inhibitor

0,238 0,013 Beta-amylase inhibitor

0,239 0,013 Huntington's disease treatment

0,281 0,056 Proliferative diseases treatment

0,257 0,031 Tentoxilysin inhibitor

0,242 0,018 Calcium antagonist

0,294 0,069 Uterine relaxant

0,292 0,068 ATP phosphoribosyltransferase inhibitor

0,264 0,040 Anticataract

0,270 0,047 Antidote

0,233 0,009 Deoxyribonuclease (pyrimidine dimer) inhibitor

0,257 0,034 Nucleoside-diphosphatase inhibitor

0,247 0,024 ATP adenylyltransferase inhibitor

0,319 0,097 CYP2D6 substrate

0,251 0,028 Tankyrase inhibitor

0,381 0,159 General pump inhibitor

0,249 0,028 Mucolytic

0,238 0,017 Arachidonic acid antagonist

0,235 0,014 Biphenyl-2,3-diol 1,2-dioxygenase inhibitor

0,228 0,007 Demethylsterigmatocystin 6-O-methyltransferase inhibitor

0,269 0,049 Antitussive

0,244 0,024 Sarcosine oxidase inhibitor

0,232 0,012 Malonate-semialdehyde dehydrogenase inhibitor

0,252 0,033 1,4-Alpha-glucan branching enzyme inhibitor

0,227 0,008 Choline acetyltransferase stimulant

0,223 0,005 Pyridoxamine-phosphate oxidase inhibitor

0,246 0,028 Aldehyde dehydrogenase inhibitor

0,242 0,024 Testosterone 17beta-dehydrogenase inhibitor

0,226 0,009 CYP26A substrate

0,253 0,036 Protein-tyrosine sulfotransferase inhibitor

0,244 0,026 Glucan 1,6-alpha-glucosidase inhibitor

0,244 0,027 Pectate lyase inhibitor

0,227 0,010 3-Isopropylmalate dehydrogenase inhibitor

0,311 0,094 Antithrombotic

0,234 0,017 Fructose-2,6-bisphosphate 2-phosphatase inhibitor

0,254 0,037 Carnosine synthase inhibitor

0,235 0,018 Cerevisin inhibitor

0,231 0,016 CDP-diacylglycerol-inositol 3-phosphatidyltransferase inhibitor

0,233 0,018 Phosphoglycerate mutase inhibitor

0,254 0,039 Sedoheptulose-bisphosphatase inhibitor

0,257 0,043 Procollagen C-endopeptidase inhibitor

0,317 0,104 Cyclic AMP phosphodiesterase inhibitor

0,231 0,017 Geranylgeranyl-diphosphate geranylgeranyltransferase inhibitor

0,216 0,003 Acetyl-CoA transferase inhibitor

0,240 0,028 Maleate isomerase inhibitor

0,229 0,017 D-lactate dehydrogenase inhibitor

0,225 0,013 Prolactin inhibitor

0,226 0,014 D-Ornithine 4,5-aminomutase inhibitor

0,253 0,042 Glutathione peroxidase inhibitor

0,227 0,016 Diaminopropionate ammonia-lyase inhibitor

0,225 0,014 Pantothenase inhibitor

0,240 0,030 Glutamin-(asparagin-)ase inhibitor

0,215 0,005 Purinergic P2X2 antagonist

0,264 0,055 Bone diseases treatment

0,232 0,023 Carboxylesterase inhibitor

0,246 0,037 Glycine C-acetyltransferase inhibitor

0,226 0,017 SMN2 expression enhancer

0,223 0,014 Mannose-6-phosphate isomerase inhibitor

0,213 0,005 Heat shock protein agonist

0,293 0,085 Antimetastatic

0,241 0,032 Acid phosphatase inhibitor

0,213 0,005 VEGF expression inhibitor

0,222 0,014 Trimethyllysine dioxygenase inhibitor

0,221 0,014 3-Oxosteroid 1-dehydrogenase inhibitor

0,232 0,026 Galacturan 1,4-alpha-galacturonidase inhibitor

0,231 0,025 Sphingosine 1-phosphate receptor antagonist

0,223 0,016 Leucine transaminase inhibitor

0,244 0,038 Gastric antisecretory

0,237 0,031 UGT2B10 substrate

0,222 0,016 (S)-carnitine 3-dehydrogenase inhibitor

0,216 0,010 Aerobactin synthase inhibitor

0,229 0,023 UGT1A7 substrate

0,218 0,012 Sorbose dehydrogenase inhibitor

0,222 0,016 Glycine transaminase inhibitor

0,228 0,023 LDLR expression enhancer

0,244 0,038 CYP1A1 inducer

0,234 0,029 tRNA adenylyltransferase inhibitor

0,215 0,010 Long-chain-fatty-acid-CoA ligase inhibitor

0,248 0,043 Interleukin 10 agonist

0,277 0,072 RNA synthesis inhibitor

0,235 0,031 Saccharolysin inhibitor

0,211 0,007 3(or 17)alpha-hydroxysteroid dehydrogenase inhibitor

0,258 0,054 Cyclic AMP modulator

0,240 0,036 Choline kinase inhibitor

0,231 0,028 DNA directed RNA polymerase inhibitor

0,226 0,023 Hexokinase inhibitor

0,224 0,021 Diiodophenylpyruvate reductase inhibitor

0,276 0,074 Antiviral (Poxvirus)

0,227 0,024 Serine-phosphoethanolamine synthase inhibitor

0,217 0,014 Formyltetrahydrofolate dehydrogenase inhibitor

0,204 0,002 L lactate dehydrogenase inhibitor

0,239 0,037 L-amino-acid oxidase inhibitor

0,228 0,026 Macular degeneration treatment

0,234 0,032 Uterine stimulant

0,228 0,026 GABA A receptor agonist

0,212 0,011 CYP3A5 inducer

0,230 0,028 Antihypotensive

0,226 0,025 Melanin inhibitor

0,222 0,021 Glutamate-1-semialdehyde 2,1-aminomutase inhibitor

0,208 0,007 Glutamate release inhibitor

0,223 0,022 Nav1.2 sodium channel blocker

0,227 0,027 Antirickettsial

0,295 0,094 Antinephritic

0,229 0,028 8-Amino-7-oxononanoate synthase inhibitor

0,259 0,060 Thiopurine S-methyltransferase inhibitor

0,223 0,024 Nicotinamidase inhibitor

0,220 0,022 Lactaldehyde reductase (NADPH) inhibitor

0,214 0,015 Uroporphyrinogen decarboxylase inhibitor

0,215 0,017 4,5-Dihydroxyphthalate decarboxylase inhibitor

0,222 0,024 Prephenate dehydrogenase inhibitor

0,238 0,040 Phenol 2-monooxygenase inhibitor

0,268 0,070 HIV attachment inhibitor

0,216 0,018 Aryl-aldehyde dehydrogenase (NADP+) inhibitor

0,221 0,024 Nitrilase inhibitor

0,217 0,020 Porphobilinogen synthase inhibitor

0,228 0,031 Phenylalanine decarboxylase inhibitor

0,219 0,021 Biotin carboxylase inhibitor

0,207 0,010 L-fuconate dehydratase inhibitor

0,213 0,016 Gluconate dehydratase inhibitor

0,220 0,024 Omega-amidase inhibitor

0,221 0,025 Acetoin dehydrogenase inhibitor

0,210 0,014 Sphingosine 1-phosphate receptor 4 antagonist

0,209 0,014 Histamine agonist

0,230 0,035 Aryl-alcohol dehydrogenase (NADP+) inhibitor

0,206 0,011 Keratoses actinic (solar) treatment

0,305 0,110 CF transmembrane conductance regulator agonist

0,232 0,037 UGT1A5 substrate

0,236 0,043 CTGF expression inhibitor

0,234 0,041 Thimet oligopeptidase inhibitor

0,198 0,005 11-Cis-retinyl-palmitate hydrolase inhibitor

0,218 0,025 Poly(3-hydroxybutyrate) depolymerase inhibitor

0,232 0,039 Ribonucleoside triphosphate reductase inhibitor

0,218 0,026 Valine-tRNA ligase inhibitor

0,216 0,023 Cysteine-S-conjugate beta-lyase inhibitor

0,202 0,009 2-Dehydro-3-deoxy-L-arabinonate dehydratase inhibitor

0,218 0,026 Polar-amino-acid-transporting ATPase inhibitor

0,200 0,008 Dihydrouracil oxidase inhibitor

0,216 0,024 Cyclooxygenase 1 inhibitor

0,224 0,032 ADP-ribosylarginine hydrolase inhibitor

0,203 0,012 2-Nitrophenol 2-monooxygenase inhibitor

0,265 0,075 Anticarcinogenic

0,265 0,076 Analgesic stimulant

0,195 0,005 Shab potassium channel blocker

0,213 0,024 L-lysine 6-transaminase inhibitor

0,207 0,018 Antineoplastic, alkylator

0,214 0,025 Contraceptive female

0,223 0,035 Acylphosphatase inhibitor

0,316 0,127 Radiosensitizer

0,228 0,040 Acetylspermidine deacetylase inhibitor

0,222 0,033 Diphosphomevalonate decarboxylase inhibitor

0,247 0,058 Histidinol dehydrogenase inhibitor

0,215 0,026 Phosphoserine phosphatase inhibitor

0,209 0,021 Beta-cyclopiazonate dehydrogenase inhibitor

0,215 0,027 Iduronate-2-sulfatase inhibitor

0,212 0,024 Riboflavin phosphotransferase inhibitor

0,216 0,028 Acetolactate decarboxylase inhibitor

0,266 0,078 MAP kinase kinase 4 inhibitor

0,220 0,033 Oxalate oxidase inhibitor

0,206 0,019 Antiprotozoal (Babesia)

0,206 0,019 Antiemphysemic

0,220 0,034 Benzoylformate decarboxylase inhibitor

0,212 0,026 Skin whitener

0,212 0,026 Cyclooxygenase inhibitor

0,201 0,015 Sodium/bile acid cotransporter inhibitor

0,206 0,021 Deoxyribonuclease X inhibitor

0,205 0,020 2-Aminoadipate transaminase inhibitor

0,222 0,037 Menstruation disorders treatment

0,204 0,018 Alkanal monooxygenase (FMN-linked) inhibitor

0,211 0,026 Endo-1,4-beta-xylanase inhibitor

0,233 0,048 Catechol oxidase inhibitor

0,205 0,020 Trehalose-phosphatase inhibitor

0,202 0,018 Diaminopimelate dehydrogenase inhibitor

0,244 0,060 Antimutagenic

0,218 0,034 Ganglioside galactosyltransferase inhibitor

0,218 0,034 Cysteine synthase inhibitor

0,197 0,014 Proline dehydrogenase inhibitor

0,201 0,018 Glutamate N-acetyltransferase inhibitor

0,201 0,018 Dihydrouracil dehydrogenase (NAD+) inhibitor

0,212 0,029 Beta-ureidopropionase inhibitor

0,230 0,047 Histone acetyltransferase inhibitor

0,192 0,009 Dry eye syndrome treatment

0,197 0,014 Dichloromuconate cycloisomerase inhibitor

0,199 0,016 2-Dehydropantolactone reductase (A-specific) inhibitor

0,208 0,025 Alcohol oxidase inhibitor

0,210 0,028 Asparagine-tRNA ligase inhibitor

0,186 0,004 Heat shock protein 70 agonist

0,303 0,121 P-glycoprotein substrate

0,207 0,025 Nav1.8 sodium channel blocker

0,197 0,015 Cysteine transaminase inhibitor

0,221 0,040 Laminaribiose phosphorylase inhibitor

0,255 0,075 Aryl-acylamidase inhibitor

0,201 0,021 D-glutamate oxidase inhibitor

0,208 0,028 Xylose isomerase inhibitor

0,234 0,054 Chemopreventive

0,214 0,034 Glucosamine-6-phosphate deaminase inhibitor

0,192 0,012 CYP4A1 substrate

0,232 0,053 Sweetener

0,207 0,029 X-His dipeptidase inhibitor

0,221 0,043 Dolichol kinase inhibitor

0,197 0,019 3-Hydroxyacyl-CoA dehydrogenase inhibitor

0,193 0,015 Glutathione reductase inhibitor

0,185 0,007 Secretase alpha stimulant

0,185 0,007 Secretase stimulant

0,235 0,057 Antiosteoporotic

0,215 0,037 Microtubule formation inhibitor

0,198 0,020 Allantoicase inhibitor

0,202 0,025 Nitric-oxide synthase stimulant

0,187 0,010 Female sexual dysfunction treatment

0,196 0,019 Cytosine deaminase inhibitor

0,208 0,031 Pyruvate dehydrogenase (lipoamide) inhibitor

0,182 0,006 Lactaldehyde dehydrogenase inhibitor

0,192 0,016 Xanthommatin reductase inhibitor

0,201 0,025 Trimethylamine-N-oxide reductase inhibitor

0,183 0,007 Histamine H2 receptor agonist

0,198 0,021 Agmatinase inhibitor

0,183 0,008 CYP2A13 substrate

0,193 0,018 Glycosylphosphatidylinositol diacylglycerol-lyase inhibitor

0,181 0,005 Lysosomal Pro-X carboxypeptidase inhibitor

0,198 0,023 4-Alpha-glucanotransferase inhibitor

0,199 0,025 Phosphonoacetate hydrolase inhibitor

0,208 0,033 Diacylglycerol cholinephosphotransferase inhibitor

0,183 0,009 2-Methylcitrate dehydratase inhibitor

0,199 0,025 Creatine kinase inhibitor

0,197 0,023 Thiol S-methyltransferase inhibitor

0,205 0,031 Succinate-CoA ligase (ADP-forming) inhibitor

0,204 0,031 Excitatory amino acid transporter EAAC1 inhibitor

0,207 0,034 Tryptophan alpha,beta-oxidase inhibitor

0,200 0,026 HDL-cholesterol increasing

0,181 0,008 Leukocyte elastase inhibitor

0,191 0,018 UDP-N-acetylglucosamine 1-carboxyvinyltransferase inhibitor

0,254 0,082 Transcription factor inhibitor

0,175 0,003 Parathyroid hormone secretion stimulant

0,175 0,002 Apolipoprotein B-100 inhibitor

0,203 0,031 Phosphate acetyltransferase inhibitor

0,197 0,024 Nuclease inhibitor

0,182 0,009 2-Methyleneglutarate mutase inhibitor

0,188 0,016 HIV-1 integrase (Overall Integration) inhibitor

0,184 0,012 Heat shock protein antagonist

0,197 0,024 VCAM1 expression inhibitor

0,183 0,011 Succinate-semialdehyde dehydrogenase inhibitor

0,207 0,036 Furin inhibitor

0,214 0,042 Gastritis treatment

0,209 0,038 Polynucleotide 5'-hydroxy-kinase inhibitor

0,175 0,004 Hyperparathyroidism treatment

0,185 0,014 Lysine decarboxylase inhibitor

0,212 0,043 FMN reductase inhibitor

0,183 0,013 Muconate cycloisomerase inhibitor

0,298 0,128 Apoptosis agonist

0,189 0,020 Aspartate-ammonia ligase (ADP-forming) inhibitor

0,192 0,023 Vasculitis treatment

0,186 0,018 Liver X receptor antagonist

0,220 0,052 Aminopeptidase B inhibitor

0,230 0,062 RNA directed DNA polymerase inhibitor

0,185 0,017 Serine-glyoxylate transaminase inhibitor

0,202 0,034 Candidapepsin inhibitor

0,207 0,039 Antineoplastic (bladder cancer)

0,206 0,039 Pyruvate carboxylase inhibitor

0,180 0,013 Guanidinopropionase inhibitor

0,172 0,004 Nav1.4 sodium channel blocker

0,185 0,018 Potassium channel (Ca-activated) activator

0,187 0,021 Polyamine oxidase inhibitor

0,215 0,048 3'-Nucleotidase inhibitor

0,175 0,009 Beta-N-acetylgalactosaminidase inhibitor

0,245 0,079 Amyotrophic lateral sclerosis treatment

0,180 0,014 Lanosterol 14 alpha demethylase inhibitor

0,182 0,016 Putrescine oxidase inhibitor

0,178 0,012 Tagaturonate reductase inhibitor

0,178 0,012 Fructuronate reductase inhibitor

0,195 0,029 O-acetylhomoserine aminocarboxypropyltransferase inhibitor

0,181 0,015 D-proline reductase (dithiol) inhibitor

0,189 0,024 1-Aminocyclopropane-1-carboxylate deaminase inhibitor

0,180 0,015 GABA inverse agonist

0,180 0,015 GABA A inverse agonist

0,183 0,019 Glutamine-pyruvate transaminase inhibitor

0,200 0,036 Cellulase inhibitor

0,248 0,084 Antibacterial

0,173 0,009 ATP deaminase inhibitor

0,185 0,022 Galactoside O-acetyltransferase inhibitor

0,172 0,009 Fatty-acyl-ethyl-ester synthase inhibitor

0,180 0,017 Sepsis treatment

0,189 0,026 Hypoxia inducible factor 1 alpha inhibitor

0,182 0,019 Astringent

0,186 0,024 Succinic dehydrogenase inhibitor

0,184 0,022 Antibacterial, ophthalmic

0,178 0,017 Succinate dehydrogenase inhibitor

0,176 0,015 Fumarylacetoacetase inhibitor

0,211 0,050 CYP1B substrate

0,169 0,008 MDM2 inhibitor

0,194 0,033 Aldehyde oxidase substrate

0,185 0,026 Interferon antagonist

0,178 0,019 CYP19 substrate

0,168 0,009 L-lactate dehydrogenase (cytochrome) inhibitor

0,193 0,034 Glutamate synthase (ferredoxin) inhibitor

0,191 0,033 N4-(beta-N-acetylglucosaminyl)-L-asparaginase inhibitor

0,181 0,023 UDP-N-acetylglucosamine-dolichyl-phosphate N-acetylglucosaminephosphotransferase inhibitor

0,167 0,009 Homospermidine synthase inhibitor

0,165 0,008 Movement disorders treatment

0,189 0,033 Gaucher disease treatment

0,259 0,103 Prostate disorders treatment

0,172 0,015 Biliverdin reductase inhibitor

0,165 0,008 Bcl2 antagonist

0,172 0,016 Glutamate (mGluR6) antagonist

0,180 0,024 Interferon gamma antagonist

0,183 0,027 Adenine deaminase inhibitor

0,178 0,022 Streptopain inhibitor

0,187 0,033 Lactosylceramide alpha-2,3-sialyltransferase inhibitor

0,162 0,009 Imidazoleglycerol-phosphate dehydratase inhibitor

0,160 0,007 Vanilloid 1 antagonist

0,180 0,027 DNA nucleotidylexotransferase inhibitor

0,199 0,046 Ethanolaminephosphotransferase inhibitor

0,175 0,022 Nitrile hydratase inhibitor

0,182 0,030 (S)-2-hydroxy-acid oxidase inhibitor

0,168 0,016 Propanediol dehydratase inhibitor

0,200 0,048 Penicillopepsin inhibitor

0,186 0,034 Formate dehydrogenase inhibitor

0,212 0,061 Growth factor agonist

0,200 0,048 Sphingosine 1-phosphate receptor 1 antagonist

0,196 0,045 Serralysin inhibitor

0,154 0,003 Retinoid X alpha receptor agonist

0,243 0,092 CYP2C9 inhibitor

0,183 0,032 Transcription factor NF kappa B inhibitor

0,191 0,040 Adenylyl-sulfate kinase inhibitor

0,177 0,026 Cathepsin H inhibitor

0,179 0,029 Glycine N-acyltransferase inhibitor

0,189 0,039 Psychosexual dysfunction treatment

0,209 0,059 P-glycoprotein 1 inhibitor

0,184 0,034 Sulfate adenylyltransferase inhibitor

0,175 0,026 Oxoglutarate dehydrogenase (lipoamide) inhibitor

0,169 0,020 Cytosole dipeptidase inhibitor

0,165 0,016 Cellobiose phosphorylase inhibitor

0,171 0,022 Urolithiasis treatment

0,150 0,002 Thyroid hormone alpha agonist

0,150 0,002 Thyroid hormone beta 1 agonist

0,150 0,002 Thyroid hormone alpha 1 agonist

0,169 0,021 TH expression enhancer

0,271 0,123 Antiprotozoal (Leishmania)

0,185 0,037 Contraceptive

0,190 0,043 Adenylate kinase inhibitor

0,175 0,028 Pim-3 kinase inhibitor

0,259 0,112 CYP1A inhibitor

0,180 0,033 Adenylate cyclase stimulant

0,152 0,005 Paget's disease treatment

0,168 0,022 N-acetylneuraminate lyase inhibitor

0,155 0,008 CYP2A5 inhibitor

0,171 0,024 AICAR transformylase inhibitor

0,171 0,025 Glycerophosphocholine cholinephosphodiesterase inhibitor

0,169 0,023 2-Aminoethylphosphonate-pyruvate transaminase inhibitor

0,162 0,017 NMDA receptor polyamine site antagonist

0,156 0,011 Phosphatidylinositol 3-kinase stimulant

0,161 0,016 Tyrosine 2,3-aminomutase inhibitor

0,179 0,034 Acetyl-CoA C-acyltransferase inhibitor

0,185 0,041 DOPA decarboxylase inhibitor

0,162 0,018 Decylcitrate synthase inhibitor

0,186 0,042 Homoserine dehydrogenase inhibitor

0,165 0,021 CMP-N-acetylneuraminate monooxygenase inhibitor

0,154 0,010 Heat shock protein 90 antagonist

0,181 0,038 Leukotriene synthesis inhibitor

0,181 0,038 Cancer procoagulant inhibitor

0,233 0,091 Muscle relaxant

0,164 0,022 Glycoprotein 3-alpha-L-fucosyltransferase inhibitor

0,168 0,026 Beta-D-fucosidase inhibitor

0,147 0,006 Orcinol 2-monooxygenase inhibitor

0,154 0,012 Nicotinic receptor alpha7 subunit antagonist

0,237 0,096 DNA synthesis inhibitor

0,176 0,035 5-Formyltetrahydrofolate cyclo-ligase inhibitor

0,182 0,041 Pyruvate dehydrogenase inhibitor

0,176 0,035 Nav1.3 sodium channel blocker

0,173 0,032 UDP-glucose-hexose-1-phosphate uridylyltransferase inhibitor

0,152 0,012 Squalene epoxidase inhibitor

0,165 0,025 Site-specific DNA-methyltransferase (adenine-specific) inhibitor

0,148 0,008 Diamine oxidase inhibitor

0,164 0,024 1,5-Anhydro-D-fructose reductase inhibitor

0,157 0,018 Arabinose isomerase inhibitor

0,177 0,039 4-Phytase inhibitor

0,170 0,032 Glycerol-3-phosphate dehydrogenase (NAD+) inhibitor

0,139 0,002 Corticotropin releasing factor antagonist

0,172 0,035 Dethiobiotin synthase inhibitor

0,151 0,014 CDK5/p25 inhibitor

0,145 0,008 CYP26 substrate

0,145 0,008 4a-Hydroxytetrahydrobiopterin dehydratase inhibitor

0,159 0,022 Guanidinobutyrase inhibitor

0,163 0,027 Narcolepsy treatment

0,153 0,016 Alternansucrase inhibitor

0,251 0,115 CYP1A2 inhibitor

0,215 0,080 Respiratory distress syndrome treatment

0,170 0,035 Glutamine-tRNA ligase inhibitor

0,170 0,035 Saccharopine dehydrogenase (NAD+, L-lysine-forming) inhibitor

0,167 0,032 NADH dehydrogenase inhibitor

0,201 0,066 Sickle-cell anemia treatment

0,155 0,020 Alpha-L-rhamnosidase inhibitor

0,181 0,046 Glutamate (mGluR5) agonist

0,155 0,021 UDP-N-acetylglucosamine diphosphorylase inhibitor

0,160 0,027 Chondroitin 6-sulfotransferase inhibitor

0,172 0,038 NAD+ kinase inhibitor

0,143 0,010 Lactate-malate transhydrogenase inhibitor

0,154 0,021 2-Hydroxy-3-oxopropionate reductase inhibitor

0,158 0,025 Cyclooxygenase 2 inhibitor

0,151 0,018 Scyllo-inosamine-4-phosphate amidinotransferase inhibitor

0,168 0,036 Lysine 2-monooxygenase inhibitor

0,197 0,065 Gastrointestinal motility stimulant

0,150 0,018 UDP-N-acetylglucosamine 2-epimerase inhibitor

0,168 0,036 NADPH oxidase inhibitor

0,140 0,008 Alpha 1 adrenoreceptor agonist

0,238 0,107 Alzheimer's disease treatment

0,152 0,021 Vitamin

0,142 0,011 CYP26 inhibitor

0,152 0,021 Xylan 1,4-beta-xylosidase inhibitor

0,156 0,026 Glucan 1,3-beta-glucosidase inhibitor

0,201 0,071 Severe acute respiratory syndrome treatment

0,152 0,021 Deoxycytidine deaminase inhibitor

0,137 0,007 Ca2+-transporting ATPase inhibitor

0,162 0,032 Venombin A inhibitor

0,171 0,041 Homogentisate 1,2-dioxygenase inhibitor

0,145 0,016 4-Hydroxy-4-methyl-2-oxoglutarate aldolase inhibitor

0,163 0,033 Acyloxyacyl hydrolase inhibitor

0,166 0,037 Adenosylmethionine-8-amino-7-oxononanoate transaminase inhibitor

0,187 0,058 5 Hydroxytryptamine 7 agonist

0,165 0,036 CYP19 inhibitor

0,152 0,024 Antitreponemal

0,165 0,037 Estrone sulfotransferase inhibitor

0,147 0,019 NOS3 expression enhancer

0,163 0,035 5-Aminolevulinate synthase inhibitor

0,144 0,016 L-lysine oxidase inhibitor

0,178 0,050 Inositol oxygenase inhibitor

0,141 0,013 Squalene-hopene cyclase inhibitor

0,166 0,039 Corticosteroid antagonist

0,153 0,026 Homocysteine desulfhydrase inhibitor

0,136 0,010 Glutamate (mGluR1) antagonist

0,157 0,030 Thiosulfate sulfurtransferase inhibitor

0,137 0,011 Monocarboxylic acid transporter 1 inhibitor

0,137 0,011 Monocarboxylic acid transporter inhibitor

0,217 0,091 Hematopoietic

0,192 0,066 Fibromyalgia syndrome treatment

0,169 0,043 Dimethylallyltranstransferase inhibitor

0,162 0,036 Aspartate transaminase inhibitor

0,156 0,031 Dihydropyrimidinase inhibitor

0,164 0,039 Restless leg syndrome treatment

0,182 0,057 Lipotropic

0,149 0,024 Malyl-CoA lyase inhibitor

0,154 0,029 Phenylalanine dehydrogenase inhibitor

0,152 0,027 D-malate dehydrogenase (decarboxylating) inhibitor

0,144 0,020 Saccharopine dehydrogenase (NAD+, L-glutamate-forming) inhibitor

0,155 0,031 UDP-glucose 4-epimerase inhibitor

0,132 0,008 Anesthetic inhalation

0,138 0,014 Cytokine modulator

0,204 0,080 Lipoprotein lipase stimulant

0,142 0,019 Amino-acid racemase inhibitor

0,163 0,040 Dolichyl-phosphate beta-D-mannosyltransferase inhibitor

0,171 0,048 Atrolysin C inhibitor

0,140 0,017 Mycodextranase inhibitor

0,180 0,057 Myosin-light-chain-phosphatase inhibitor

0,153 0,030 Diacylglycerol kinase inhibitor

0,136 0,013 (S)-2-Methylmalate dehydratase inhibitor

0,134 0,011 4-Pyridoxolactonase inhibitor

0,205 0,083 Antidiarrheal

0,207 0,085 Diuretic

0,188 0,066 Antidepressant, Imipramin-like

0,154 0,032 Galactoside 2-alpha-L-fucosyltransferase inhibitor

0,138 0,016 Maleate hydratase inhibitor

0,160 0,038 Membrane dipeptidase inhibitor

0,136 0,016 Epoxide hydrolase substrate

0,134 0,014 Autophagy inducer

0,148 0,028 UGT2B11 substrate

0,153 0,033 Pyrimidine-deoxynucleoside 2'-dioxygenase inhibitor

0,130 0,010 Erythro-3-hydroxyaspartate ammonia-lyase inhibitor

0,232 0,113 Chloride channel blocker

0,146 0,027 Mannonate dehydratase inhibitor

0,143 0,024 Arginyltransferase inhibitor

0,131 0,012 Anticholelithogenic

0,146 0,027 2-Pyrocatechuate decarboxylase inhibitor

0,130 0,011 Heme oxygenase inhibitor

0,149 0,030 Ribonuclease inhibitor

0,149 0,030 Neuropeptide Y1 antagonist

0,144 0,026 Trimerelysin II inhibitor

0,162 0,043 Formaldehyde dehydrogenase (glutathione) inhibitor

0,201 0,083 Polarisation inhibitor

0,125 0,006 Glutamate uptake inhibitor

0,168 0,050 D-Octopine dehydrogenase inhibitor

0,154 0,036 Alanine transaminase inhibitor

0,143 0,025 Isomaltulose synthase inhibitor

0,154 0,036 Heparan-alpha-glucosaminide N-acetyltransferase inhibitor

0,164 0,046 NAD(P)+ transhydrogenase (AB-specific) inhibitor

0,128 0,010 Glycine transporter 2 inhibitor

0,141 0,024 Inositol 1,4,5-trisphosphate 3-kinase inhibitor

0,158 0,041 HCV NS3-helicase inhibitor

0,138 0,021 Anthranilate synthase inhibitor

0,137 0,020 3-Deoxy-7-phosphoheptulonate synthase inhibitor

0,169 0,052 Alcohol dehydrogenase inhibitor

0,137 0,021 Globoside alpha-N-acetylgalactosaminyltransferase inhibitor

0,140 0,023 Phosphoenolpyruvate carboxykinase (ATP) inhibitor

0,136 0,019 Cathepsin G inhibitor

0,153 0,037 Methylenetetrahydrofolate dehydrogenase (NADP+) inhibitor

0,134 0,018 High-mannose-oligosaccharide beta-1,4-N-acetylglucosaminyltransferase inhibitor

0,129 0,013 Acetylcholine M3 receptor agonist

0,126 0,011 CYP2C2 substrate

0,163 0,048 Procollagen-lysine 5-dioxygenase inhibitor

0,121 0,006 CYP1B1 inducer

0,139 0,024 4-Hydroxybenzoate-CoA ligase inhibitor

0,134 0,020 Ribulose-bisphosphate carboxylase inhibitor

0,129 0,015 Oxaloacetate tautomerase inhibitor

0,163 0,049 Endoglycosylceramidase inhibitor

0,141 0,026 D-alanine transaminase inhibitor

0,133 0,019 Purine biosynthesis inhibitor

0,195 0,082 Phospholipase A1 inhibitor

0,170 0,057 Alkaline phosphatase inhibitor

0,169 0,056 Thioredoxin reductase inhibitor

0,151 0,039 Beta lactamase inhibitor

0,145 0,033 Acetate-CoA ligase inhibitor

0,135 0,023 Gluconokinase inhibitor

0,130 0,017 Alpha-L-fucosidase inhibitor

0,135 0,023 Aspartate-semialdehyde dehydrogenase inhibitor

0,148 0,036 2-Aminohexano-6-lactam racemase inhibitor

0,187 0,075 Cerebrovascular disordes treatment

0,130 0,018 Sterol 24-C-methyltransferase inhibitor

0,145 0,033 Nicotinamide-nucleotide adenylyltransferase inhibitor

0,137 0,025 Glycoprotein-fucosylgalactoside alpha-N-acetylgalactosaminyltransferase inhibitor

0,128 0,016 Kallikrein 3 inhibitor

0,136 0,024 Diguanidinobutanase inhibitor

0,115 0,003 Urate transporter 1 inhibitor

0,117 0,005 Farnesoid X receptor agonist

0,115 0,004 Oxytocin agonist

0,173 0,062 GABA C receptor antagonist

0,122 0,010 Necroptosis inhibitor

0,115 0,004 PRL phosphatase inhibitor

0,156 0,045 Protein kinase B alpha inhibitor

0,141 0,030 Insulin secretagoues

0,131 0,021 (R)-aminopropanol dehydrogenase inhibitor

0,163 0,052 Chitin synthase inhibitor

0,123 0,013 Alpha,alpha-trehalose phosphorylase inhibitor

0,131 0,021 N-acetylglucosamine-6-phosphate deacetylase inhibitor

0,140 0,029 Photinus-luciferin 4-monooxygenase (ATP-hydrolysing) inhibitor

0,147 0,037 L-ascorbate peroxidase inhibitor

0,138 0,028 Polynucleotide adenylyltransferase inhibitor

0,148 0,038 Aspartate kinase inhibitor

0,192 0,083 Free radical scavenger

0,140 0,030 Histone deacetylase SIRT2 inhibitor

0,135 0,027 Isoamylase inhibitor

0,166 0,057 Alcohol dehydrogenase (NADP+) inhibitor

0,120 0,012 Carboxypeptidase U inhibitor

0,131 0,023 Malate dehydrogenase (NADP+) inhibitor

0,142 0,035 Malate dehydrogenase (oxaloacetate-decarboxylating) inhibitor

0,138 0,031 3-Hydroxybutyrate dehydrogenase inhibitor

0,126 0,019 Contraceptive male

0,136 0,029 Colony stimulating factor agonist

0,166 0,059 Calcium channel blocker

0,132 0,025 Glyceraldehyde-3-phosphate dehydrogenase (NADP+) inhibitor

0,136 0,029 Cholestenone 5alpha-reductase inhibitor

0,120 0,014 Indoleamine-pyrrole 2,3-dioxygenase inhibitor

0,131 0,025 Phosphatidylglycerol-membrane-oligosaccharide glycerophosphotransferase inhibitor

0,133 0,027 Phosphoenolpyruvate carboxykinase (diphosphate) inhibitor

0,220 0,114 Transcription factor STAT3 inhibitor

0,129 0,024 Protein kinase C stimulant

0,141 0,036 Agaritine gamma-glutamyltransferase inhibitor

0,153 0,048 Antihemorrhagic

0,132 0,026 Hair growth stimulant

0,122 0,017 NAT1 substrate

0,137 0,032 tRNA (cytosine-5-)-methyltransferase inhibitor

0,122 0,017 Glutamate (mGluR8) agonist

0,144 0,040 MAO inhibitor

0,139 0,034 Deoxycytidylate 5-hydroxymethyltransferase inhibitor

0,132 0,028 Uridine nucleosidase inhibitor

0,134 0,030 Cyclin-dependent kinase 7 inhibitor

0,190 0,086 P-glycoprotein inhibitor

0,145 0,041 5 Hydroxytryptamine 2A agonist

0,126 0,022 Urocanate hydratase inhibitor

0,109 0,005 Endothelial nitric-oxide synthase inhibitor

0,135 0,031 Anabolic

0,153 0,050 Methylglutamate dehydrogenase inhibitor

0,154 0,051 2-Nitropropane dioxygenase inhibitor

0,140 0,037 Asparaginase inhibitor

0,164 0,061 Glucosyl transferase inhibitor

0,143 0,040 X-Pro dipeptidase inhibitor

0,115 0,012 Interleukin 1a antagonist

0,122 0,019 Peroxisome proliferator-activated receptor gamma antagonist

0,129 0,027 CDC25A inhibitor

0,115 0,013 ABCA1 expression enhancer

0,127 0,025 Tyrosine-protein kinase TYRO 10 inhibitor

0,106 0,004 CYP24 inhibitor

0,136 0,034 Cystathionine beta-synthase inhibitor

0,107 0,005 1-Deoxy-D-xylulose-5-phosphate reductoisomerase inhibitor

0,123 0,021 Carboxypeptidase M inhibitor

0,127 0,025 Argininosuccinate lyase inhibitor

0,129 0,028 Phosphoglycolate phosphatase inhibitor

0,152 0,051 Electrolyte absorption antagonist

0,148 0,047 Tryptophan 2-monooxygenase inhibitor

0,137 0,037 Glutamate dehydrogenase inhibitor

0,105 0,004 Thyroid hormone agonist

0,147 0,047 Poly(ADP-ribose) glycohydrolase inhibitor

0,152 0,051 Antidiabetic (type 1)

0,103 0,003 Protease (Human cytomegalovirus) inhibitor

0,125 0,026 CYP4F substrate

0,116 0,017 Aralkylamine dehydrogenase inhibitor

0,122 0,023 Inositol-3-phosphate synthase inhibitor

0,146 0,048 Kexin inhibitor

0,127 0,030 Ketohexokinase inhibitor

0,119 0,021 Branched-chain-amino-acid transaminase inhibitor

0,132 0,034 Bipolar disorder treatment

0,133 0,035 Ornithine-oxo-acid transaminase inhibitor

0,137 0,040 Arginine deiminase inhibitor

0,120 0,023 Glycerophosphocholine phosphodiesterase inhibitor

0,112 0,015 CYP4F12 substrate

0,132 0,035 D-benzoylarginine-4-nitroanilide amidase inhibitor

0,119 0,023 Glycerol kinase inhibitor

0,124 0,028 Oxaloacetate decarboxylase inhibitor

0,114 0,018 Alpha adrenoreceptor agonist

0,119 0,024 Gentamicin 2''-nucleotidyltransferase inhibitor

0,109 0,013 N-acetylglucosaminyldiphosphoundecaprenol N-acetyl-beta-D-mannosaminyltransferase inhibitor

0,120 0,024 Betaine-aldehyde dehydrogenase inhibitor

0,123 0,028 Galactosylgalactosylglucosylceramidase inhibitor

0,101 0,006 Glycine N-choloyltransferase inhibitor

0,126 0,032 2-Oxoaldehyde dehydrogenase (NAD+) inhibitor

0,131 0,037 NAT2 substrate

0,117 0,022 4-Hydroxy-2-oxoglutarate aldolase inhibitor

0,159 0,065 Cytosol alanyl aminopeptidase inhibitor

0,154 0,060 3-Hydroxyanthranilate oxidase inhibitor

0,113 0,019 Long-chain-acyl-CoA dehydrogenase inhibitor

0,103 0,009 3-Alpha-hydroxysteroid dehydrogenase (B-specific) inhibitor

0,124 0,031 Beta amyloid protein antagonist

0,112 0,019 Alpha-1,6-mannosyl-glycoprotein 6-beta-N-acetylglucosaminyltransferase inhibitor

0,131 0,038 5 Hydroxytryptamine 4A antagonist

0,115 0,022 Nitric-oxide synthase inhibitor

0,118 0,025 S-methyl-5-thioribose kinase inhibitor

0,126 0,033 Acyl-CoA dehydrogenase (NADP+) inhibitor

0,118 0,026 Aminolevulinate transaminase inhibitor

0,097 0,004 CYP24 substrate

0,127 0,034 Thiamine-phosphate diphosphorylase inhibitor

0,106 0,014 Hydroperoxide dehydratase inhibitor

0,129 0,037 Shikimate kinase inhibitor

0,121 0,029 Glycine N-benzoyltransferase inhibitor

0,126 0,034 CYP1B1 inhibitor

0,103 0,012 Propionate CoA-transferase inhibitor

0,096 0,004 CXC chemokine 1 receptor antagonist

0,131 0,040 Hyperprolactinemia treatment

0,111 0,020 Formimidoylglutamase inhibitor

0,115 0,023 2-Methylcitrate synthase inhibitor

0,116 0,025 Sulfinoalanine decarboxylase inhibitor

0,121 0,030 Ribonuclease U2 inhibitor

0,096 0,005 Potassium channel Kv1.3 blocker

0,123 0,032 3-Deoxy-8-phosphooctulonate synthase inhibitor

0,107 0,017 Quinate 5-dehydrogenase inhibitor

0,112 0,021 Cyanate hydratase inhibitor

0,213 0,123 Cytochrome P450 inhibitor

0,099 0,009 4-Hydroxyphenylacetate 1-monooxygenase inhibitor

0,117 0,028 Homoserine O-succinyltransferase inhibitor

0,225 0,136 Antidiabetic

0,110 0,021 Polyamine biosynthesis inhibitor

0,123 0,034 Cystathionine beta-lyase inhibitor

0,105 0,017 Catechol 2,3-dioxygenase inhibitor

0,121 0,033 Acetylcholine release stimulant

0,104 0,016 Mycolysin inhibitor

0,203 0,116 Hepatic disorders treatment

0,105 0,017 Alpha,alpha-phosphotrehalase inhibitor

0,122 0,035 CTP synthase inhibitor

0,108 0,021 Peroxisome proliferator-activated receptor antagonist

0,109 0,022 L-3-cyanoalanine synthase inhibitor

0,105 0,018 Steroid sulfotransferase inhibitor

0,305 0,218 Antineoplastic (non-Hodgkin's lymphoma)

0,112 0,025 Factor XIIIa stimulant

0,102 0,015 Diiodotyrosine transaminase inhibitor

0,124 0,038 Pyridoxal kinase inhibitor

0,100 0,013 Endonuclease (influenza) inhibitor

0,201 0,115 Antiinfertility, female

0,108 0,022 4-Hydroxybenzoate decarboxylase inhibitor

0,108 0,022 Alpha,alpha-trehalose-phosphate synthase (UDP-forming) inhibitor

0,094 0,008 Heme oxygenase stimulant

0,093 0,007 Kinesin antagonist

0,093 0,007 Kinesin-like protein 1 inhibitor

0,108 0,023 Phospholipase D inhibitor

0,091 0,005 CYP3A11 substrate

0,114 0,029 Diacylglycerol O-acyltransferase inhibitor

0,130 0,044 Alpha,alpha-trehalase inhibitor

0,103 0,018 4-Hydroxybenzoate 1-hydroxylase inhibitor

0,115 0,030 Aminopeptidase I inhibitor

0,103 0,018 Tocolytic

0,136 0,051 Caspase 3 inhibitor

0,130 0,045 Luteinizing hormone-releasing hormone agonist

0,131 0,047 Lysozyme inhibitor

0,140 0,055 Histone deacetylase class III inhibitor

0,115 0,031 Nicotinic alpha2beta4 receptor agonist

0,102 0,018 Chitin deacetylase inhibitor

0,104 0,020 [acyl-carrier-protein] S-malonyltransferase inhibitor

0,089 0,005 Calcium-sensing receptor antagonist

0,095 0,012 3-Hydroxyanthranilate 3,4-dioxygenase inhibitor

0,111 0,027 Isocitrate dehydrogenase (NADP+) inhibitor

0,085 0,002 L lactate dehydrogenase A inhibitor

0,121 0,038 Argininosuccinate synthase inhibitor

0,146 0,063 Iodide peroxidase inhibitor

0,121 0,038 5-Methyltetrahydropteroyltriglutamate-homocysteine S-methyltransferase inhibitor

0,098 0,015 1-Alkyl-2-acetylglycerol O-acyltransferase inhibitor

0,170 0,087 Male reproductive disfunction treatment

0,097 0,014 ACK1 kinase inhibitor

0,116 0,034 Telomerase inhibitor

0,186 0,104 Antiepileptic

0,089 0,008 Thyroid hormone antagonist

0,121 0,040 Isocitrate dehydrogenase (NAD+) inhibitor

0,116 0,035 Guanylate kinase inhibitor

0,095 0,014 Immunoglobulin E antagonist

0,101 0,020 Peptidoglycan beta-N-acetylmuramidase inhibitor

0,116 0,036 Capillary fragility treatment

0,206 0,125 Cytostatic

0,097 0,016 D-lactate-2-sulfatase inhibitor

0,107 0,027 [myelin basic protein]-arginine N-methyltransferase inhibitor

0,136 0,056 Antibiotic

0,211 0,131 Ophthalmic drug

0,113 0,032 Indole-3-acetaldehyde reductase (NADPH) inhibitor

0,127 0,046 JNK mitogen-activated protein kinase inhibitor

0,101 0,021 Malate synthase inhibitor

0,097 0,017 Butyrylcholinesterase inhibitor

0,096 0,016 NMDA receptor polyamine site agonist

0,088 0,008 Malonyl-CoA decarboxylase inhibitor

0,132 0,052 Bacterial efflux pump inhibitor

0,111 0,031 Neolactotetraosylceramide alpha-2,3-sialyltransferase inhibitor

0,199 0,120 Channel-conductance-controlling ATPase inhibitor

0,127 0,048 Homocitrate synthase inhibitor

0,117 0,038 Leucine-tRNA ligase inhibitor

0,107 0,028 Neuropeptide FF agonist

0,107 0,028 Neuropeptide FF2 agonist

0,104 0,025 2-Dehydro-3-deoxy-phosphogluconate aldolase inhibitor

0,098 0,020 Sphingosine 1-phosphate receptor 5 agonist

0,104 0,026 Tryptophan-tRNA ligase inhibitor

0,098 0,020 CDC25C inhibitor

0,099 0,021 Enoyl-CoA hydratase inhibitor

0,118 0,041 Transthyretin amyloid fibril formation inhibitor

0,120 0,042 Trypanothione-disulfide reductase inhibitor

0,079 0,001 L lactate dehydrogenase B inhibitor

0,079 0,001 L lactate dehydrogenase B4 inhibitor

0,097 0,020 UDP-glucuronate decarboxylase inhibitor

0,105 0,028 3(or 17)beta-hydroxysteroid dehydrogenase inhibitor

0,123 0,045 Rhodopsin kinase inhibitor

0,141 0,063 Endothelin-converting enzyme inhibitor

0,135 0,058 Dyspepsia treatment

0,109 0,032 Beta-glucosidase inhibitor

0,175 0,098 Endopeptidase inhibitor

0,095 0,019 Altronate dehydratase inhibitor

0,086 0,010 Elastase inhibitor

0,111 0,035 Galactosylceramide sulfotransferase inhibitor

0,101 0,025 Heat shock protein 70 antagonist

0,090 0,014 Glutathione reductase (NADPH) stimulant

0,090 0,014 Glutathione reductase stimulant

0,102 0,026 L-Serine ammonia-lyase inhibitor

0,078 0,003 Cholesterol esterase inhibitor

0,100 0,024 Sulfonylureas

0,129 0,053 Narcotic antagonist

0,130 0,054 DNA directed DNA polymerase inhibitor

0,083 0,008 Isopentenyl-diphosphate DELTA-isomerase inhibitor

0,099 0,024 L-iditol 2-dehydrogenase inhibitor

0,116 0,041 Interferon agonist

0,084 0,009 CDC25B inhibitor

0,081 0,007 Prostaglandin-E synthase inhibitor

0,121 0,047 Cell adhesion inhibitor

0,128 0,054 Glutathione synthase inhibitor

0,096 0,022 Hydroxyquinol 1,2-dioxygenase inhibitor

0,129 0,055 Calcium channel (voltage-sensitive) blocker

0,113 0,039 DNA methyltransferase I inhibitor

0,124 0,050 Antispirochetal

0,104 0,030 Alanine carboxypeptidase inhibitor

0,091 0,018 Inducible nitric-oxide synthase inhibitor

0,092 0,019 Deoxyhypusine synthase inhibitor

0,099 0,026 Glucosamine 6-phosphate N-acetyltransferase inhibitor

0,081 0,008 Dihydroorotate oxidase inhibitor

0,136 0,063 Sepiapterin reductase inhibitor

0,155 0,082 Alkylator

0,102 0,030 N-acetylglucosamine kinase inhibitor

0,106 0,034 Glutamate dehydrogenase (NADP+) inhibitor

0,086 0,014 Sleep apnea treatment

0,105 0,033 Beta-N-acetylhexosaminidase inhibitor

0,077 0,006 Anhydrotetracycline monooxygenase inhibitor

0,097 0,025 SULT2A1 substrate

0,131 0,060 Protease inhibitor

0,093 0,022 3-Oxoacyl-[acyl-carrier-protein] synthase III inhibitor

0,088 0,017 CXC chemokine receptor antagonist

0,136 0,065 Carboxypeptidase E inhibitor

0,095 0,024 Asparagine-oxo-acid transaminase inhibitor

0,123 0,052 Phenylalanine 4-hydroxylase inhibitor

0,111 0,041 Cell wall synthesis inhibitor

0,087 0,016 Potassium channel small-conductance Ca-activated 3 activator

0,095 0,025 Substance P antagonist

0,079 0,009 Glycolaldehyde dehydrogenase inhibitor

0,112 0,042 Bis(5'-adenosyl)-triphosphatase inhibitor

0,106 0,036 Granulocyte macrophage colony stimulating factor agonist

0,088 0,019 D-Serine ammonia-lyase inhibitor

0,090 0,021 Linoleate isomerase inhibitor

0,104 0,035 Cystathionine gamma-lyase inhibitor

0,085 0,016 Tannase inhibitor

0,108 0,039 Granzyme B inhibitor

0,117 0,048 Adenylylsulphatase inhibitor

0,099 0,029 L-aminoadipate-semialdehyde dehydrogenase inhibitor

0,089 0,020 Parathyroid hormone antagonist

0,095 0,025 Dextransucrase inhibitor

0,093 0,023 mRNA (guanine-N7-)-methyltransferase inhibitor

0,187 0,118 Gestagen antagonist

0,141 0,072 Diabetic retinopathy treatment

0,118 0,049 Glycine hydroxymethyltransferase inhibitor

0,073 0,004 CC chemokine 9 receptor antagonist

0,131 0,062 Antineoplastic antimetabolite

0,097 0,028 Dihydrolipoamide S-acetyltransferase inhibitor

0,109 0,041 Dihydropteroate synthase inhibitor

0,098 0,029 3-Oxoacyl-[acyl-carrier-protein] synthase inhibitor

0,090 0,022 Arabinogalactan endo-1,4-beta-galactosidase inhibitor

0,102 0,034 UDP-N-acetylmuramate dehydrogenase inhibitor

0,110 0,042 Octadecanal decarbonylase inhibitor

0,110 0,043 tRNA cytidylyltransferase inhibitor

0,080 0,014 Phosphomannomutase inhibitor

0,083 0,016 Alpha 2 adrenoreceptor agonist

0,207 0,140 Vasodilator

0,108 0,042 Ethanolamine kinase inhibitor

0,072 0,005 Cardiac myosin stimulant

0,092 0,026 Bacterial leucyl aminopeptidase inhibitor

0,086 0,020 Thromboxane A2 antagonist

0,104 0,038 Isocitrate lyase inhibitor

0,070 0,004 Calcitonin stimulant

0,088 0,023 Prephenate dehydratase inhibitor

0,100 0,034 Phosphoribulokinase inhibitor

0,102 0,036 tRNA (guanine-N2-)-methyltransferase inhibitor

0,081 0,016 Plus-end-directed kinesin ATPase inhibitor

0,070 0,005 Vitamin K-like

0,079 0,014 Dihydroorotate dehydrogenase inhibitor

0,080 0,015 Amylosucrase inhibitor

0,125 0,060 Somatostatin 2 agonist

0,077 0,012 CYP17 substrate

0,112 0,047 Phosphofructokinase-1 inhibitor

0,090 0,026 Protein C (activated) inhibitor

0,102 0,038 Succinate-hydroxymethylglutarate CoA-transferase inhibitor

0,097 0,033 6-Phosphofructo-2-kinase inhibitor

0,100 0,035 Estrogen agonist

0,086 0,022 Butyryl-CoA dehydrogenase inhibitor

0,086 0,022 Thioether S-methyltransferase inhibitor

0,093 0,029 Aspartate 1-decarboxylase inhibitor

0,107 0,044 CMP-N-acylneuraminate phosphodiesterase inhibitor

0,072 0,008 Macrophage scavenger receptor antagonist

0,098 0,035 Glutamate (mGluR group I) antagonist

0,090 0,027 Protein kinase B stimulant

0,104 0,041 Hydroxymethylglutaryl-CoA reductase inhibitor

0,100 0,037 Chymotrypsin inhibitor

0,078 0,015 Glutamate (mGluR group III) antagonist

0,110 0,047 Antiacne

0,082 0,019 Stipitatonate decarboxylase inhibitor

0,071 0,008 Dichloromethane dehalogenase inhibitor

0,095 0,032 Thromboxane antagonist

0,097 0,034 Hypoxanthine phosphoribosyltransferase inhibitor

0,090 0,029 DNA methylase inhibitor

0,078 0,017 Hepatocyte nuclear factor agonist

0,078 0,017 Hepatocyte nuclear factor 4 alpha agonist

0,176 0,115 Pancreatic disorders treatment

0,088 0,026 Maleylacetoacetate isomerase inhibitor

0,077 0,016 Ubiquitin-protein ligase inhibitor

0,103 0,042 Pyrroline-5-carboxylate reductase inhibitor

0,073 0,012 Carboxypeptidase B inhibitor

0,074 0,014 2-Ethylmalate synthase inhibitor

0,099 0,039 Arginine decarboxylase inhibitor

0,101 0,041 G-protein-coupled bile acid receptor 1 agonist

0,065 0,005 Retinoid X receptor agonist

0,080 0,019 D-Alanine-poly(phosphoribitol) ligase inhibitor

0,107 0,047 Aromatic-amino-acid transaminase inhibitor

0,072 0,012 Tyrosine phenol-lyase inhibitor

0,094 0,034 Ribose-5-phosphate isomerase inhibitor

0,066 0,007 GABA B receptor antagonist

0,097 0,038 Shock treatment

0,084 0,025 3-Methyl-2-oxobutanoate dehydrogenase (lipoamide) inhibitor

0,099 0,040 Phenylalanine-tRNA ligase inhibitor

0,085 0,026 Inositol 1,4,5-triphosphate receptor antagonist

0,084 0,025 UGT2B7Y substrate

0,084 0,025 UGT2B7H substrate

0,077 0,018 5-Aminovalerate transaminase inhibitor

0,087 0,029 N-acylneuraminate cytidylyltransferase inhibitor

0,075 0,016 Bcl-xL inhibitor

0,063 0,004 ATPase (Vacuolar H+) inhibitor

0,073 0,014 Prostaglandin-I synthase inhibitor

0,102 0,043 Oligo-1,6-glucosidase inhibitor

0,195 0,136 Xenobiotic-transporting ATPase inhibitor

0,097 0,039 [hydroxymethylglutaryl-CoA reductase (NADPH)] kinase inhibitor

0,085 0,027 3-Hydroxydecanoyl-[acyl-carrier-protein] dehydratase inhibitor

0,084 0,026 Aldose reductase inhibitor

0,099 0,041 Antihypertriglyceridemic

0,062 0,005 Sodium/hydrogen exchanger 3 inhibitor

0,091 0,034 3-Dehydroquinate dehydratase inhibitor

0,061 0,003 Juvenile-hormone esterase inhibitor

0,093 0,036 4-Hydroxybenzoate 3-monooxygenase inhibitor

0,089 0,032 Sphingosine kinase inhibitor

0,073 0,016 Serine carboxypeptidase inhibitor

0,086 0,029 Aldose 1-epimerase inhibitor

0,119 0,063 Meprin A inhibitor

0,060 0,004 Cholesterol ester transfer protein antagonist

0,064 0,008 NADPH oxidoreductase inhibitor

0,108 0,052 Extrapyramidal disorders treatment

0,076 0,020 2-Methyl-branched-chain-enoyl-CoA reductase inhibitor

0,083 0,027 Transcription factor AP-1 inhibitor

0,090 0,034 Sucrose synthase inhibitor

0,127 0,071 Glutathione S-transferase substrate

0,254 0,199 Muramoyltetrapeptide carboxypeptidase inhibitor

0,080 0,025 Acidifying agent gastric

0,144 0,089 Tumour necrosis factor antagonist

0,070 0,015 5-Alpha-reductase inhibitor

0,088 0,033 15-Lipoxygenase inhibitor

0,073 0,019 dCTP deaminase inhibitor

0,081 0,026 Procollagen glucosyltransferase inhibitor

0,074 0,020 UGT2B4D substrate

0,074 0,020 UGT2B4E substrate

0,074 0,020 UGT2B19 substrate

0,074 0,020 UGT2B30 substrate

0,074 0,020 UGT2B23 substrate

0,065 0,011 Glucagon-like peptide 1 receptor antagonist

0,077 0,023 Shaker potassium channel blocker

0,076 0,022 Aromatase inhibitor

0,064 0,011 Androst-4-ene-3,17-dione monooxygenase inhibitor

0,108 0,054 Glutamate decarboxylase inhibitor

0,073 0,020 AMPA receptor agonist

0,057 0,003 Imidazoline receptor antagonist

0,070 0,017 Carboxy-cis,cis-muconate cyclase inhibitor

0,059 0,006 ADAM10 endopeptidase inhibitor

0,080 0,028 Phenylalanine 2-monooxygenase inhibitor

0,081 0,028 MAO B inhibitor

0,117 0,064 Pyroglutamyl-peptidase I inhibitor

0,082 0,030 MAO A inhibitor

0,067 0,014 Na+ K+ transporting ATPase inhibitor

0,075 0,023 Plasminogen activator stimulant

0,085 0,034 Serine-tRNA ligase inhibitor

0,081 0,029 Somatostatin agonist

0,077 0,025 Mandelate racemase inhibitor

0,075 0,024 Osmotic diuretic

0,066 0,015 Aryl hydrocarbon receptor antagonist

0,078 0,026 NMDA 2 receptor antagonist

0,102 0,050 Xanthine dehydrogenase inhibitor

0,068 0,017 Andropause treatment

0,080 0,030 dUTP diphosphatase inhibitor

0,074 0,023 CMP-KDO synthase inhibitor

0,065 0,014 Methylaspartate mutase inhibitor

0,070 0,020 Glutamate (mGluR3) agonist

0,096 0,046 Glycerol dehydrogenase inhibitor

0,090 0,040 Alanine dehydrogenase inhibitor

0,174 0,125 Thiol protease inhibitor

0,082 0,032 3,4-Dihydroxyphenylacetate 2,3-dioxygenase inhibitor

0,065 0,016 NMDA 2A receptor antagonist

0,073 0,024 Prolyl endopeptidase inhibitor

0,097 0,048 Ribose-5-phosphate-ammonia ligase inhibitor

0,058 0,009 HMG CoA reductase inhibitor

0,059 0,010 Peptidyl-prolyl cis-trans isomerase inhibitor

0,084 0,035 Pyruvate, phosphate dikinase inhibitor

0,075 0,026 Protein-tyrosine phosphatase 2C inhibitor

0,059 0,011 Pregnane X receptor antagonist

0,080 0,031 Antiprotozoal (Histomonas)

0,054 0,006 Puromycin-sensitive aminopeptidase inhibitor

0,085 0,036 Amylo-alpha-1,6-glucosidase inhibitor

0,075 0,027 Cellulose synthase (UDP-forming) inhibitor

0,111 0,063 Liver cirrhosis treatment

0,065 0,017 Potassium channel (Inward rectifier) blocker

0,059 0,011 5-Alpha-reductase 1 inhibitor

0,087 0,039 Cushing's syndrome treatment

0,070 0,022 Biotin-[propionyl-CoA-carboxylase (ATP-hydrolysing)] ligase inhibitor

0,056 0,008 Orsellinate decarboxylase inhibitor

0,079 0,032 NMN nucleosidase inhibitor

0,072 0,024 Androgen agonist

0,070 0,023 Glucan 1,3-alpha-glucosidase inhibitor

0,077 0,030 Beta-1,4-mannosyl-glycoprotein 4-beta-N-acetylglucosaminyltransferase inhibitor

0,089 0,042 Deoxycytidine kinase inhibitor

0,064 0,016 3-Galactosyl-N-acetylglucosaminide 4-alpha-L-fucosyltransferase inhibitor

0,074 0,026 Alpha-1,6-mannosyl-glycoprotein 2-beta-N-acetylglucosaminyltransferase inhibitor

0,051 0,004 Octopamine agonist

0,065 0,018 Tubulin GTPase inhibitor

0,070 0,022 Deoxyadenosine kinase inhibitor

0,100 0,053 Hypercalcemia treatment

0,092 0,045 Adenine nucleotide translocase inhibitor

0,080 0,033 Dihydrodipicolinate synthase inhibitor

0,072 0,025 Chalcone isomerase inhibitor

0,066 0,019 Epoxide hydrolase inhibitor

0,064 0,018 Cannabinoid receptor antagonist

0,076 0,029 H+/K+-transporting ATPase inhibitor

0,075 0,029 Hot flush treatment

0,070 0,024 Estrogen-related receptor alpha antagonist

0,080 0,034 Glutamate (mGluR5) antagonist

0,060 0,014 Antiviral (Parainfluenza)

0,070 0,024 Sucrose alpha-glucosidase inhibitor

0,120 0,074 Adenylate cyclase inhibitor

0,058 0,013 Potassium channel Kv1.1 blocker

0,117 0,072 Orexin receptor antagonist

0,087 0,042 Beta 1 adrenoreceptor agonist

0,050 0,005 Nociceptin (N/OFQ) receptor agonist

0,073 0,028 Carbamoyl-serine ammonia-lyase inhibitor

0,093 0,048 Transcription factor STAT6 inhibitor

0,069 0,024 Glucan 1,4-alpha-glucosidase inhibitor

0,067 0,022 Aldose reductase 2 inhibitor

0,078 0,034 Mannitol-1-phosphate 5-dehydrogenase inhibitor

0,093 0,048 Retinoic acid beta receptor agonist

0,188 0,143 Platelet aggregation inhibitor

0,072 0,028 Acyl-CoA dehydrogenase inhibitor

0,063 0,019 D(-)-tartrate dehydratase inhibitor

0,061 0,016 Aminoacyl-tRNA hydrolase inhibitor

0,172 0,127 Metabolic disease treatment

0,048 0,004 ICAM 1 antagonist

0,096 0,052 Nucleotide diphosphatase inhibitor

0,056 0,012 Antimitotic, Podophyllotoxin-like

0,047 0,003 Fatty acid elongase inhibitor

0,102 0,058 GABA transporter 3 inhibitor

0,072 0,029 Malate dehydrogenase (decarboxylating) inhibitor

0,089 0,046 Dopachrome isomerase inhibitor

0,059 0,016 Riboflavin synthase inhibitor

0,060 0,017 Delayed rectifier potassium channel blocker

0,087 0,044 Kynureninase inhibitor

0,064 0,021 Sucrose-phosphate phosphatase inhibitor

0,085 0,043 Alpha glucosidase inhibitor

0,046 0,004 Chloride/bicarbonate exchanger inhibitor

0,068 0,026 Tryptophan 5 hydroxylase inhibitor

0,064 0,022 Cytidylate kinase inhibitor

0,059 0,017 Strombine dehydrogenase inhibitor

0,074 0,032 Alpha,alpha-trehalose phosphorylase (configuration-retaining) inhibitor

0,084 0,042 Cysteine-tRNA ligase inhibitor

0,080 0,038 Pantoate-beta-alanine ligase inhibitor

0,050 0,008 Riboflavin kinase inhibitor

0,051 0,009 Vitamin D3 hydroxylase inhibitor

0,063 0,021 Arginine racemase inhibitor

0,065 0,024 Protein N-acetylglucosaminyltransferase inhibitor

0,055 0,014 CXC chemokine 2 receptor antagonist

0,103 0,062 Interleukin 10 antagonist

0,066 0,026 3'(2'),5'-Bisphosphate nucleotidase inhibitor

0,078 0,038 Protocatechuate 3,4-dioxygenase inhibitor

0,071 0,031 Prostaglandin agonist

0,045 0,005 Cortisone alpha-reductase inhibitor

0,081 0,041 X-Trp aminopeptidase inhibitor

0,046 0,006 Dolichyldiphosphatase inhibitor

0,069 0,028 NAD+ diphosphatase inhibitor

0,061 0,021 Nucleoside-triphosphate-adenylate kinase inhibitor

0,050 0,010 Acetyl-CoA transferase 2 inhibitor

0,047 0,007 Glucagon receptor antagonist

0,053 0,013 Potassium channel KCNQ blocker

0,086 0,046 5 Hydroxytryptamine 3 agonist

0,064 0,024 1,3-Beta-glucan synthase inhibitor

0,091 0,051 Choline sulfotransferase inhibitor

0,090 0,051 Aminopeptidase microsomal inhibitor

0,070 0,030 GTP cyclohydrolase I inhibitor

0,053 0,014 Oleamide hydrolase inhibitor

0,150 0,111 Janus tyrosine kinase 2 inhibitor

0,074 0,035 Collagenase inhibitor

0,074 0,035 Sphingosine 1-phosphate receptor 3 antagonist

0,081 0,042 Glutaminyl-tRNA synthase (glutamine-hydrolysing) inhibitor

0,064 0,026 Aldehyde dehydrogenase 1A2 inhibitor

0,059 0,021 Pyridoxamine-oxaloacetate transaminase inhibitor

0,073 0,035 [acetyl-CoA carboxylase] kinase inhibitor

0,065 0,027 Nicotinate phosphoribosyltransferase inhibitor

0,064 0,026 Vanilloid 4 antagonist

0,066 0,028 Glutamate (mGluR1) agonist

0,077 0,039 3-Dehydroquinate synthase inhibitor

0,044 0,006 Nucleotidyltransferase inhibitor

0,066 0,028 Assemblin inhibitor

0,088 0,050 MAO B substrate

0,057 0,020 Peroxisome proliferator-activated receptor alpha agonist

0,092 0,054 Insulin sensitizer

0,050 0,013 CYP4F8 substrate

0,047 0,010 Protein-tyrosine phosphatase epsilon inhibitor

0,061 0,024 Methylcrotonoyl-CoA carboxylase inhibitor

0,050 0,014 Endo-beta-N-acetylglucosaminidase inhibitor

0,050 0,013 Carbonic anhydrase XIV inhibitor

0,039 0,003 Imidazoline I2 receptor antagonist

0,072 0,036 Phosphoglucomutase inhibitor

0,065 0,029 CC chemokine receptor agonist

0,065 0,029 Chemokine receptor agonist

0,052 0,016 Potassium channel KCNQ activator

0,052 0,016 Potassium channel (Voltage-sensitive) activator

0,052 0,016 Delayed rectifier potassium channel activator

0,071 0,035 UDP-glucose 6-dehydrogenase inhibitor

0,064 0,028 Cathepsin E inhibitor

0,084 0,048 Gastricsin inhibitor

0,082 0,046 1-Pyrroline-5-carboxylate dehydrogenase inhibitor

0,065 0,030 Glutamate (mGluR) antagonist

0,070 0,035 Androgen antagonist

0,040 0,005 Phosphodiesterase IX inhibitor

0,050 0,015 Adenylate cyclase I inhibitor

0,084 0,049 Trans-octaprenyltranstransferase inhibitor

0,090 0,055 Chelator

0,067 0,032 Arginine kinase inhibitor

0,052 0,017 Chelator, Iron

0,044 0,009 Nicotinamide-nucleotide amidase inhibitor

0,144 0,110 Antioxidant

0,126 0,092 Obsessive-compulsive disorder treatment

0,055 0,020 Thymidine kinase 1 inhibitor

0,039 0,005 Apolipoprotein B inhibitor

0,191 0,157 Transcription factor STAT inhibitor

0,070 0,036 Methylthioadenosine nucleosidase inhibitor

0,055 0,022 Asparaginyl endopeptidase inhibitor

0,060 0,026 Cysteine dioxygenase inhibitor

0,162 0,128 CYP2A11 substrate

0,071 0,038 Dipeptidyl peptidase I inhibitor

0,053 0,020 Thymidine-triphosphatase inhibitor

0,111 0,078 CDK2/cyclin A inhibitor

0,073 0,040 Tubulin-tyrosine ligase inhibitor

0,084 0,051 Glutathione-disulfide reductase inhibitor

0,073 0,041 Citrate (Si)-synthase inhibitor

0,041 0,008 TRPA1 antagonist

0,214 0,181 CYP17 inhibitor

0,060 0,027 Liver X receptor alpha agonist

0,071 0,038 Alpha-mannosidase inhibitor

0,043 0,011 Valine-pyruvate transaminase inhibitor

0,050 0,017 Prostaglandin-E2 synthase 1 inhibitor

0,062 0,030 Selectin E antagonist

0,093 0,062 T cell inhibitor

0,054 0,022 Chorismate mutase inhibitor

0,035 0,003 Retinoid X gamma receptor antagonist

0,105 0,073 Kallikrein inhibitor

0,035 0,004 Thyroid hormone beta agonist

0,109 0,078 Bronchodilator

0,042 0,011 Peroxisome proliferator-activated receptor delta agonist

0,064 0,033 Acetylcholine muscarinic agonist

0,061 0,030 Protein-glutamate O-methyltransferase inhibitor

0,064 0,033 GABA receptor antagonist

0,181 0,150 Vascular (periferal) disease treatment

0,105 0,074 Cytidine deaminase inhibitor

0,191 0,160 5 Hydroxytryptamine 1E antagonist

0,079 0,049 Carbamoyl-phosphate synthase (ammonia) inhibitor

0,078 0,048 Histidine ammonia-lyase inhibitor

0,157 0,127 Lymphocytopoiesis inhibitor

0,037 0,007 Acetyl-CoA transferase 1 inhibitor

0,062 0,032 1-Phosphofructokinase inhibitor

0,067 0,038 Cathepsin L inhibitor

0,108 0,079 Fibroblast growth factor 2 antagonist

0,084 0,055 Phosphorylase kinase inhibitor

0,031 0,002 Actin polymerization inhibitor

0,039 0,010 Free fatty acid receptor 1 agonist

0,111 0,082 Cocain dependency treatment

0,030 0,001 L lactate dehydrogenase A4 inhibitor

0,079 0,050 Nav1.7 sodium channel blocker

0,074 0,045 Preterm labor treatment

0,042 0,013 Luteinizing hormone-releasing hormone antagonist

0,050 0,021 Kallikrein 13 inhibitor

0,067 0,039 Dihydrofolate synthase inhibitor

0,061 0,032 D-Lysine 5,6-aminomutase inhibitor

0,075 0,047 TTK protein kinase inhibitor

0,048 0,019 CYP2C18 inhibitor

0,032 0,004 Potassium channel (Inward rectifier) 1 blocker

0,059 0,031 UGT2B20 substrate

0,040 0,012 Phosphodiesterase 9A inhibitor

0,030 0,002 Corticotropin releasing factor 1 receptor antagonist

0,059 0,032 Uracil phosphoribosyltransferase inhibitor

0,037 0,009 I(Ks) voltage-gated potassium channel blocker

0,055 0,028 Biotin-[methylmalonyl-CoA-carboxytransferase] ligase inhibitor

0,049 0,022 Phosphorylase a inhibitor

0,042 0,015 Homoisocitrate dehydrogenase inhibitor

0,062 0,035 ATP diphosphatase inhibitor

0,041 0,014 Neuronal nitric-oxide synthase inhibitor

0,028 0,001 L lactate dehydrogenase C inhibitor

0,028 0,001 L lactate dehydrogenase C4 inhibitor

0,037 0,010 Dihydrofolate reductase inhibitor

0,040 0,014 5-Alpha-reductase 2 inhibitor

0,046 0,019 Glycolipid 2-alpha-mannosyltransferase inhibitor

0,058 0,031 Thromboxane synthase inhibitor

0,066 0,039 Phospholipase D1 inhibitor

0,034 0,007 Sigma 3 receptor antagonist

0,062 0,036 Gamma-glutamyl hydrolase inhibitor

0,048 0,022 G protein-coupled receptor agonist

0,043 0,017 Endothelin receptor antagonist

0,046 0,020 Tartrate decarboxylase inhibitor

0,059 0,033 Aspartate ammonia-lyase inhibitor

0,034 0,008 CYP27A1 inhibitor

0,066 0,041 Proline racemase inhibitor

0,052 0,026 Cytokine production stimulant

0,061 0,036 Angiotensin-converting enzyme inhibitor

0,032 0,007 CYP27A substrate

0,049 0,024 Actin depolymerization stimulant

0,065 0,040 UDP-N-acetylmuramoylalanine-D-glutamate ligase inhibitor

0,029 0,005 Nucleotidyltransferase (FIV) inhibitor

0,029 0,004 Thyroid hormone alpha 1 antagonist

0,126 0,102 Antiviral (HIV)

0,060 0,036 Peroxisome proliferator-activated receptor agonist

0,037 0,013 Cadherin antagonist

0,090 0,066 Glutamine synthetase inhibitor

0,056 0,031 Beta 3 adrenoreceptor antagonist

0,066 0,042 FAD diphosphatase inhibitor

0,078 0,053 Aminopeptidase N inhibitor

0,036 0,013 2-Aminohexanoate transaminase inhibitor

0,029 0,005 Adenosine A1 receptor agonist enhancer

0,063 0,039 NAD+ nucleosidase inhibitor

0,224 0,200 Analgesic

0,055 0,031 Antifungal (Pneumocystis)

0,048 0,024 CPB protease inhibitor

0,087 0,064 Histamine N-methyltransferase inhibitor

0,064 0,041 Dynamin GTPase inhibitor

0,111 0,087 Thromboxane synthase stimulant

0,028 0,005 3-Oxoacyl-[acyl-carrier-protein] synthase II inhibitor

0,062 0,038 Glutamate formimidoyltransferase inhibitor

0,045 0,022 Neuropathy treatment

0,058 0,035 Sepiapterin deaminase inhibitor

0,052 0,029 Calcium channel L-type blocker

0,051 0,028 Betaine-homocysteine S-methyltransferase inhibitor

0,073 0,050 Aralkylamine N-acetyltransferase inhibitor

0,055 0,032 Potassium channel Kv1.5 blocker

0,032 0,009 Antidote, organophosphates

0,051 0,029 3-Phosphoshikimate 1-carboxyvinyltransferase inhibitor

0,027 0,004 Toll-Like receptor 8 agonist

0,164 0,141 Folate antagonist

0,038 0,016 Transaldolase inhibitor

0,173 0,151 Immunostimulant

0,042 0,020 CXC chemokine 4 receptor antagonist

0,050 0,028 N-acetylglucosaminyldiphosphodolichol N-acetylglucosaminyltransferase inhibitor

0,091 0,070 Amidase inhibitor

0,039 0,018 Glycerate kinase inhibitor

0,040 0,019 Sucrose phosphorylase inhibitor

0,050 0,029 GABA uptake inhibitor

0,048 0,027 Oxytocin antagonist

0,042 0,021 Antithrombocytopenic

0,026 0,005 Necrosis treatment

0,054 0,033 Mannosyl-oligosaccharide glucosidase inhibitor

0,027 0,007 CYP27 substrate

0,075 0,055 Glycine N-methyltransferase inhibitor

0,038 0,018 Ryanodine receptor 1 antagonist

0,038 0,018 Ryanodine receptor antagonist

0,057 0,037 Hydroxylysine kinase inhibitor

0,133 0,113 CYP2A10 substrate

0,044 0,024 Glutaryl-CoA dehydrogenase inhibitor

0,024 0,004 Retinoid X beta receptor antagonist

0,056 0,036 MAP-kinase-activated kinase 1 inhibitor

0,042 0,022 Bombesin 2 receptor antagonist

0,047 0,027 Glutamate (mGluR group II) agonist

0,043 0,023 Alpha-N-acetylgalactosaminide alpha-2,6-sialyltransferase inhibitor

0,066 0,046 Potassium channel (Inward rectifier) activator

0,066 0,046 Potassium channel (ATP-sensitive) activator

0,034 0,014 Phosphopyruvate hydratase inhibitor

0,096 0,077 Histidine decarboxylase inhibitor

0,044 0,025 Sodium-dependent vitamin C transporter 2 inhibitor

0,049 0,030 Glucose-1-phosphate adenylyltransferase inhibitor

0,028 0,008 Tankyrase 2 inhibitor

0,025 0,006 Microsomal triglyceride transfer protein inhibitor

0,063 0,044 Integrin alpha2beta1 antagonist

0,044 0,025 Glutamate (mGluR2) agonist

0,073 0,054 Aspartate carbamoyltransferase inhibitor

0,070 0,051 NAD(P)H dehydrogenase (quinone) inhibitor

0,031 0,012 Alpha 2b adrenoreceptor agonist

0,031 0,012 Vanilloid 4 agonist

0,039 0,020 Liver X receptor beta agonist

0,050 0,031 Phosphoketolase inhibitor

0,028 0,009 CYP17A substrate

0,041 0,022 Uridine kinase inhibitor

0,034 0,016 Geranyltranstransferase inhibitor

0,041 0,023 Glucosylceramidase inhibitor

0,036 0,017 D-Alanine-alanyl-poly(glycerolphosphate) ligase inhibitor

0,048 0,030 Nicotinic acid receptor 1 agonist

0,060 0,042 Tubulinyl-Tyr carboxypeptidase inhibitor

0,063 0,044 3-Oxoacid CoA-transferase inhibitor

0,023 0,005 Acetyl-CoA carboxylase 1 inhibitor

0,023 0,004 CC chemokine 10 receptor antagonist

0,036 0,018 Sodium/glucose cotransporter 1 inhibitor

0,069 0,051 Potassium channel activator

0,052 0,034 Phosphoglycerate dehydrogenase inhibitor

0,076 0,058 Glutamate carboxypeptidase inhibitor

0,074 0,056 MAP3K8 inhibitor

0,035 0,017 Uridine phosphorylase inhibitor

0,072 0,055 Glutathione S-transferase inhibitor

0,048 0,031 Deoxyguanosine kinase inhibitor

0,065 0,048 Saccharopine dehydrogenase (NADP+, L-lysine-forming) inhibitor

0,025 0,008 Progesterone receptor B1 antagonist

0,024 0,007 Urotensin II agonist

0,185 0,168 Undecaprenyl-phosphate mannosyltransferase inhibitor

0,026 0,009 Ketosteroid monooxygenase inhibitor

0,191 0,174 Pulmonary hypertension treatment

0,079 0,062 Chemokine receptor antagonist

0,020 0,003 RAS guanyl releasing protein antagonist

0,020 0,003 HIV fusion inhibitor

0,035 0,019 Thymine dioxygenase inhibitor

0,094 0,077 MAO A substrate

0,078 0,062 Ornithine decarboxylase inhibitor

0,037 0,021 Cortisol sulfotransferase inhibitor

0,059 0,043 Mannosidase inhibitor

0,051 0,035 Malate dehydrogenase (oxaloacetate-decarboxylating) (NADP+) inhibitor

0,045 0,029 Fructose 5-dehydrogenase (NADP+) inhibitor

0,068 0,052 Carnitine O-octanoyltransferase inhibitor

0,090 0,074 Lipoxygenase inhibitor

0,037 0,021 Membrane-oligosaccharide glycerophosphotransferase inhibitor

0,048 0,033 Bombesin antagonist

0,037 0,021 2-Methylacyl-CoA dehydrogenase inhibitor

0,038 0,023 Diphosphate-serine phosphotransferase inhibitor

0,060 0,045 Beta-fructofuranosidase inhibitor

0,019 0,004 Carbonic anhydrase XV inhibitor

0,048 0,033 Fumarate hydratase inhibitor

0,029 0,014 Inositol 1,4,5-trisphosphate 3-kinase B inhibitor

0,051 0,036 Glucose-6-phosphate translocase inhibitor

0,037 0,022 Nitric oxide donor

0,074 0,060 Melanocortin agonist

0,040 0,026 Histamine H4 receptor antagonist

0,045 0,031 [citrate-(pro-3S)-lyase] thiolesterase inhibitor

0,020 0,006 Formyl peptide receptor antagonist

0,041 0,026 Cyclohexadienyl dehydrogenase inhibitor

0,038 0,024 Fucokinase inhibitor

0,110 0,095 Bcr-Abl kinase inhibitor

0,054 0,040 GABA A receptor antagonist

0,039 0,025 Phosphoglycerate phosphatase inhibitor

0,046 0,032 Nerve growth factor antagonist

0,051 0,037 Nuclear receptor subfamily 4, group A, member 2 agonist

0,069 0,055 5-Lipoxygenase inhibitor

0,063 0,049 Glutamate-cysteine ligase inhibitor

0,029 0,015 Phenylpyruvate tautomerase inhibitor

0,063 0,049 Steroid DELTA-isomerase inhibitor

0,041 0,027 Vibriolysin inhibitor

0,030 0,016 Alpha glucosidase I inhibitor

0,048 0,035 Secretase beta 1 inhibitor

0,196 0,183 Systemic lupus erythematosus treatment

0,053 0,039 Pyruvate, water dikinase inhibitor

0,031 0,018 Glucose-dependent insulinotropic receptor agonist

0,050 0,036 Secretase beta inhibitor

0,034 0,021 Alpha-1,3-mannosyl-glycoprotein 2-beta-N-acetylglucosaminyltransferase inhibitor

0,018 0,005 Retinoic acid receptor gamma antagonist

0,019 0,006 Myristoyl transferase 1 inhibitor

0,018 0,005 Orexin receptor 2 antagonist

0,017 0,005 CYP19 inducer

0,020 0,008 Dopamine D2 agonist inducer

0,046 0,033 S-methyl-5-thioadenosine phosphorylase inhibitor

0,060 0,048 Chymase inhibitor

0,039 0,026 Kainate receptor agonist

0,017 0,005 Retinoic acid alpha receptor antagonist

0,035 0,023 2-(acetamidomethylene)succinate hydrolase inhibitor

0,049 0,037 Deoxyhypusine monooxygenase inhibitor

0,019 0,007 Antiprotozoal activity enhancer

0,058 0,046 Alpha 2d adrenoreceptor antagonist

0,043 0,031 Putrescine carbamoyltransferase inhibitor

0,016 0,004 5 Lipoxygenase activating protein inhibitor

0,079 0,067 Neurolysin inhibitor

0,027 0,015 MARK1 protein inhibitor

0,041 0,029 [Isocitrate dehydrogenase (NADP+)] kinase inhibitor

0,015 0,003 Retinoid X receptor antagonist

0,021 0,010 Prostaglandin A1 agonist

0,064 0,053 NMDA receptor agonist

0,024 0,013 Procollagen-proline 3-dioxygenase inhibitor

0,058 0,047 Phosphodiesterase 1B inhibitor

0,031 0,021 Osteoclast antagonist

0,031 0,021 AMP deaminase inhibitor

0,056 0,045 Cathepsin D inhibitor

0,061 0,050 GMP synthase inhibitor

0,026 0,016 GABA transporter inhibitor

0,032 0,021 Sphingosine 1-phosphate receptor 4 agonist

0,036 0,026 Protocatechuate 4,5-dioxygenase inhibitor

0,035 0,025 Ribose-5-phosphate adenylyltransferase inhibitor

0,358 0,348 Gluconate 2-dehydrogenase (acceptor) inhibitor

0,036 0,026 MAP kinase kinase inhibitor

0,072 0,063 Hydroxyacylglutathione hydrolase inhibitor

0,088 0,079 Antismoking

0,027 0,017 Cyclomaltodextrin glucanotransferase inhibitor

0,030 0,021 Glutamate (mGluR1a) agonist

0,030 0,021 Glutamate (mGluR5a) agonist

0,045 0,036 Spermine synthase inhibitor

0,013 0,003 Integrin alpha5beta6 antagonist

0,075 0,066 Antidote, heavy metal

0,048 0,038 Spermidine synthase inhibitor

0,043 0,034 Protein-tyrosine phosphatase yopH inhibitor

0,013 0,004 Cholesterol monooxygenase (side-chain-cleaving) inhibitor

0,019 0,010 CYP11B1 substrate

0,039 0,030 Glycogenin glucosyltransferase inhibitor

0,022 0,013 Helicase E1 (Human papillomavirus) inhibitor

0,014 0,005 CYP19A1 substrate

0,063 0,054 Tryptophan synthase inhibitor

0,073 0,064 Vascular endothelial growth factor 1 antagonist

0,012 0,003 Retinoid X alpha receptor antagonist

0,051 0,042 Beta galactosidase inhibitor

0,064 0,056 Leukotriene C antagonist

0,013 0,004 CYP7A1 substrate

0,224 0,216 Transcription factor NF kappa B stimulant

0,224 0,216 Transcription factor stimulant

0,047 0,038 [3-methyl-2-oxobutanoate dehydrogenase (lipoamide)] kinase inhibitor

0,043 0,035 Hydroxypyruvate reductase inhibitor

0,023 0,015 Retinoic acid receptor agonist

0,054 0,046 Phosphodiesterase VII inhibitor

0,037 0,029 Phosphoserine transaminase inhibitor

0,025 0,017 TGF beta receptor type II kinase inhibitor

0,014 0,007 Proline transporter inhibitor

0,014 0,006 CYP7 substrate

0,020 0,012 Sphingosine 1-phosphate receptor 3 agonist

0,044 0,036 Polypeptide N-acetylgalactosaminyltransferase inhibitor

0,028 0,020 GRP78 expression inhibitor

0,049 0,041 Purinergic P2X antagonist

0,055 0,048 Tyrosine transaminase inhibitor

0,043 0,035 Bis(5'-nucleosyl)-tetraphosphatase (asymmetrical) inhibitor

0,039 0,032 Cholesterol absorption inhibitor

0,022 0,015 Sphingosine kinase stimulant

0,035 0,028 Alpha 1L adrenoreceptor antagonist

0,025 0,018 Tartrate dehydrogenase inhibitor

0,012 0,005 Synaptic vesicle protein SV2, levetiracetam binding site antagonist

0,045 0,038 UDP-N-acetylglucosamine-lysosomal-enzyme N-acetylglucosaminephosphotransferase inhibitor

0,053 0,046 Methionine adenosyltransferase inhibitor

0,012 0,005 Growth hormone releasing factor antagonist

0,014 0,007 Thyroid hormone beta 1 antagonist

0,020 0,014 Vesicular glutamate transport inhibitor

0,060 0,054 Integrin alphaVbeta1 antagonist

0,013 0,006 Thyroid hormone alpha antagonist

0,013 0,007 Thyroid hormone beta antagonist

0,030 0,024 Nociceptin (N/OFQ) receptor antagonist

0,022 0,016 Fatty acid oxidation inhibitor

0,033 0,026 Glucose-6-phosphate isomerase inhibitor

0,040 0,034 Orotidylate decarboxylase inhibitor

0,038 0,032 [hydroxymethylglutaryl-CoA reductase (NADPH)]-phosphatase inhibitor

0,011 0,005 Prostaglandin EP4 agonist

0,108 0,102 Tumour necrosis factor alpha antagonist

0,018 0,012 Endothelin receptor agonist

0,038 0,033 dCMP deaminase inhibitor

0,020 0,015 Guanylate cyclase 1 inhibitor

0,048 0,043 Acetylcholine M5 receptor antagonist

0,019 0,014 GABA transporter 1 inhibitor

0,029 0,024 Alpha-N-acetylneuraminate alpha-2,8-sialyltransferase inhibitor

0,070 0,065 Glutamate synthase (NADPH) inhibitor

0,050 0,045 Potassium channel (ATP-sensitive) blocker

0,031 0,027 Methionyl aminopeptidase inhibitor

0,036 0,031 Nucleoside phosphoacylhydrolase inhibitor

0,043 0,039 Histone-arginine N-methyltransferase inhibitor

0,045 0,041 Nucleoside deoxyribosyltransferase inhibitor

0,044 0,039 Bis(5'-nucleosyl)-tetraphosphatase (symmetrical) inhibitor

0,022 0,018 Beta lactamase TEM-1 inhibitor

0,046 0,042 Nicotinate-nucleotide diphosphorylase (carboxylating) inhibitor

0,104 0,100 Phospholipase inhibitor

0,043 0,038 Polyphosphate kinase inhibitor

0,009 0,005 Beta lactamase II inhibitor

0,016 0,012 Myristoyl transferase inhibitor

0,041 0,037 Oligonucleotidase inhibitor

0,019 0,015 LFA antagonist

0,019 0,015 LFA-1 antagonist

0,054 0,050 Phosphogluconate dehydrogenase (decarboxylating) inhibitor

0,032 0,028 [Skp1-protein]-hydroxyproline N-acetylglucosaminyltransferase inhibitor

0,037 0,033 Potassium channel small-conductance Ca-activated blocker

0,071 0,067 Leukotriene antagonist

0,103 0,099 Gastrointestinal disorders treatment

0,084 0,080 Neuropeptide Y antagonist

0,007 0,004 Amylin agonist

0,008 0,005 CXC chemokine 5 receptor antagonist

0,028 0,024 Somatostatin 1 antagonist

0,016 0,012 Imidazoline I1 receptor antagonist

0,054 0,050 Amino-acid N-acetyltransferase inhibitor

0,032 0,029 Somatostatin 5 antagonist

0,009 0,005 Retinoic acid beta receptor antagonist

0,011 0,008 Histone acetylation inducer

0,015 0,012 Glutamate (mGluR2) antagonist

0,013 0,009 dTMP kinase (Mycobacterium tuberculosis) inhibitor

0,039 0,036 Dipeptidyl peptidase II inhibitor

0,012 0,009 Retinoic acid receptor antagonist

0,027 0,024 Melatonin 2 agonist

0,016 0,013 Gestagen-like

0,018 0,015 17-Beta-hydroxysteroid dehydrogenase 5 inhibitor

0,017 0,014 CYP27A1 substrate

0,032 0,030 Thymidine phosphorylase inhibitor

0,031 0,029 Lysine carboxypeptidase inhibitor

0,047 0,045 CC chemokine 2 receptor antagonist

0,063 0,061 Polo-like kinase-1 inhibitor

0,021 0,019 NAD(P)H dehydrogenase (quinone) 1 inhibitor

0,079 0,077 Neuropeptide antagonist

0,026 0,024 Theanine hydrolase inhibitor

0,029 0,027 Postcoital contraceptive

0,011 0,010 Demethylmacrocin O-methyltransferase inhibitor

0,014 0,012 Acetyl-CoA carboxylase inhibitor

0,092 0,091 Anthranilate phosphoribosyltransferase inhibitor

0,020 0,019 Endothelin B receptor antagonist

0,018 0,017 2-Hydroxyacylsphingosine 1-beta-galactosyltransferase inhibitor

0,012 0,011 Progesterone receptor A antagonist

0,010 0,008 MarA inhibitor

0,031 0,030 2-Isopropylmalate synthase inhibitor

0,043 0,042 Prostaglandin antagonist

0,021 0,020 Dopamine D2B antagonist

0,026 0,025 CYP7B1 substrate

0,026 0,025 CYP7B substrate

0,022 0,021 Methionyl aminopeptidase 1 inhibitor

0,035 0,034 Baculoviral IAP repeat-containing protein inhibitor

0,011 0,010 Macrocin O-methyltransferase inhibitor

0,016 0,015 UDP-3-O-acyl-N-acetylglucosamine deacetylase inhibitor

0,014 0,013 Vitamin D-like

0,082 0,081 Phosphodiesterase I inhibitor

**
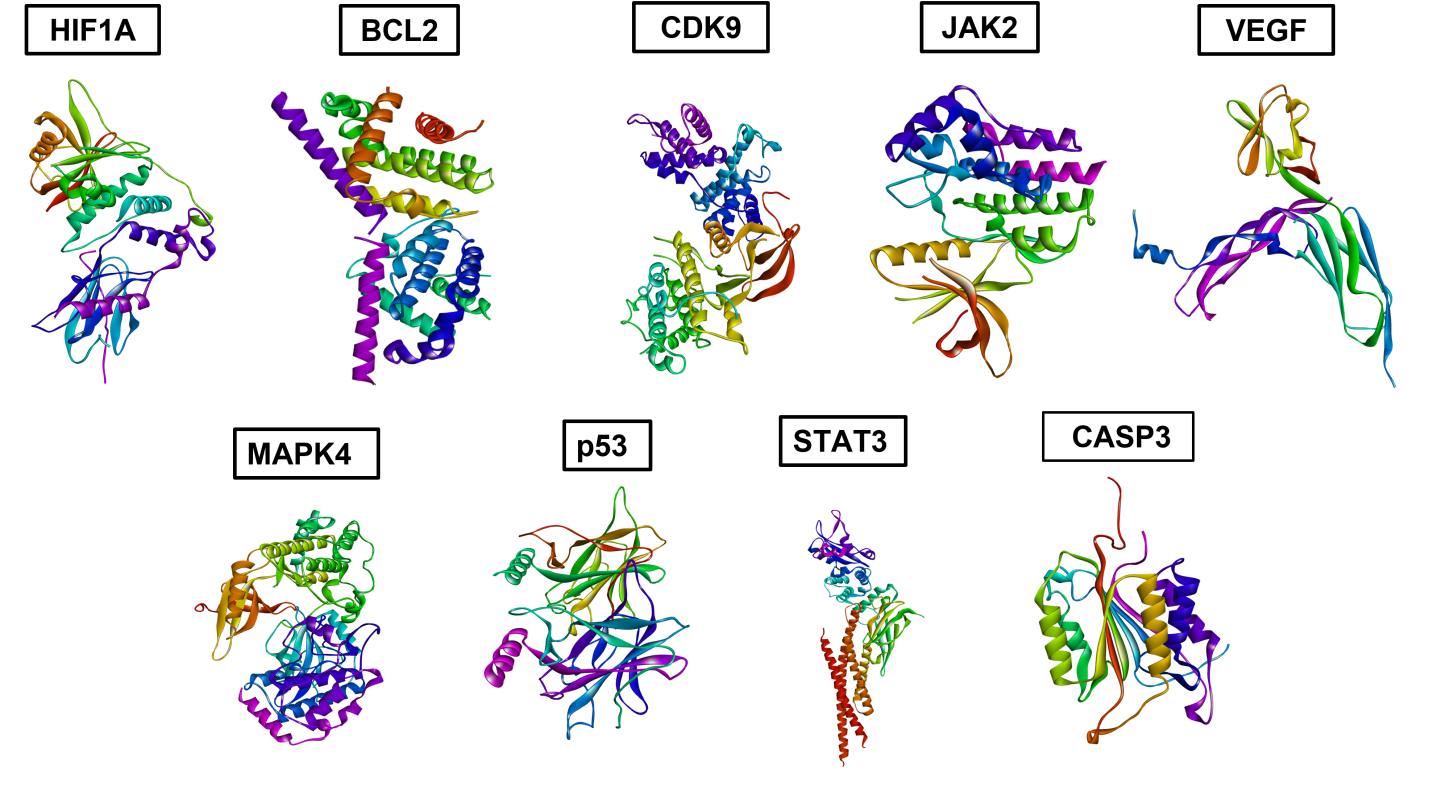
**

**Figure S1: 3D structures of various human proteins, highlighting their unique conformations and structural features through distinct color coding**


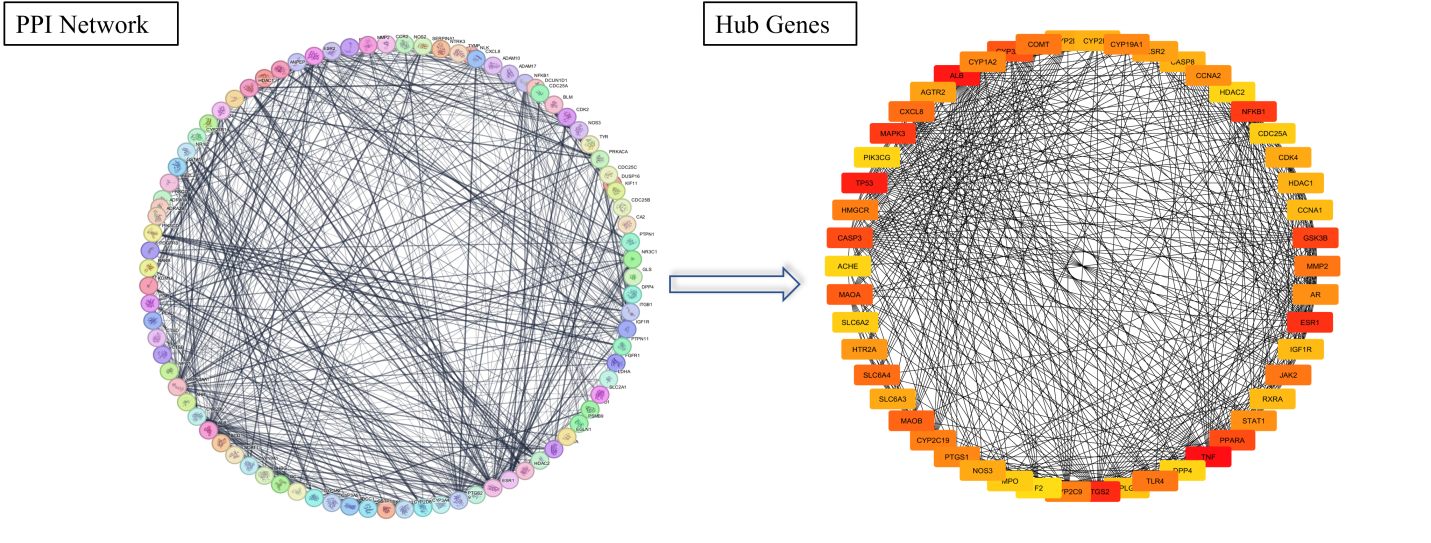


**Figure S2: Interaction network of potential targets of p-cymene** **showcasing various connections between proteins.** The left side displays the complete network, while the right side highlights the identified hub genes, which play central roles in the network.


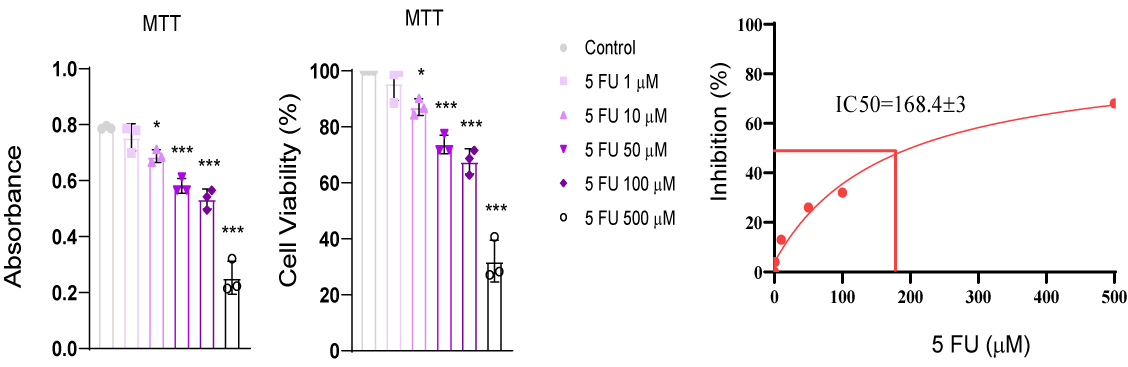


**Figure S3: IC50 of 5FU in HepG2 cells.** 5FU dose dependently reduced cell viability with an IC50 value of 168 µM. One-way ANOVA followed by Dunnett’s multiple comparison test was used to analyze the data, confirming the significant differences among the treatment groups. Significance levels were denoted as: *** ≤ 0.001, * ≤ 0.05 (Treated groups vs. negative control).


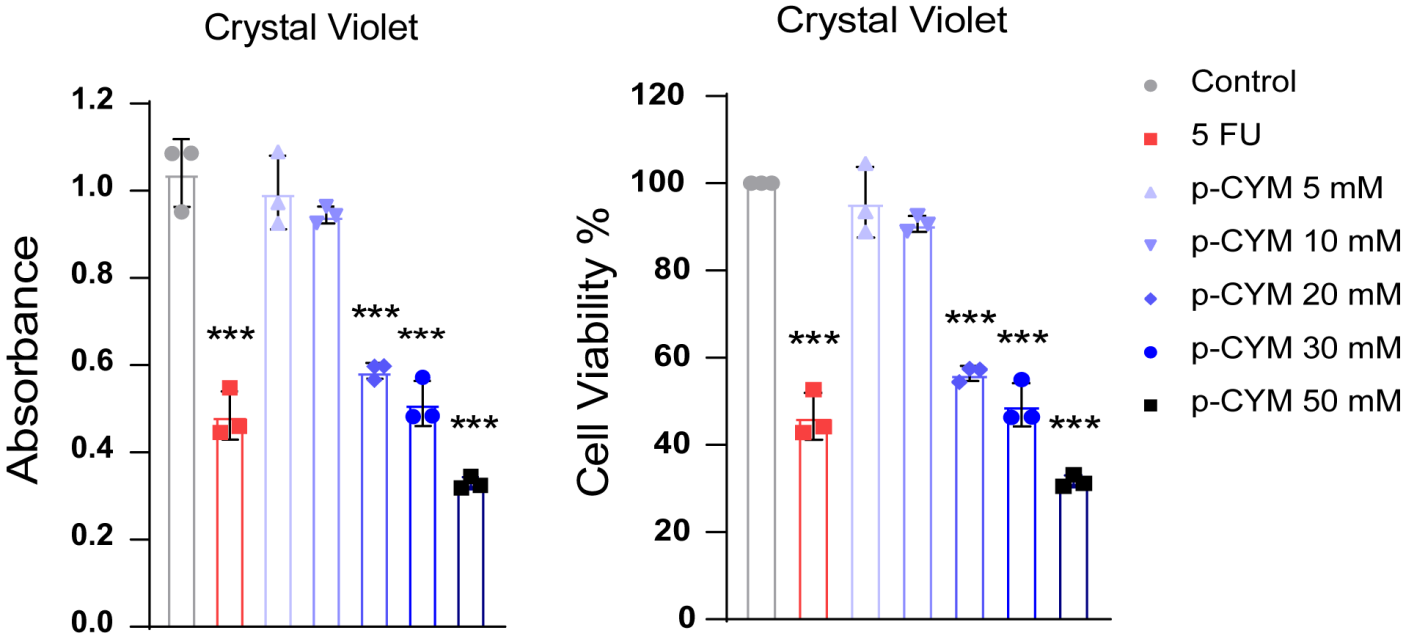


**Figure S4:** **Crystal Violet assay assessing the impact of p-Cymene on HepG2 cell viability.** The left panel shows absorbance values, while the right panel indicates percentage viability, with significant reductions at higher p-Cymene concentrations. Statistical analysis was performed using one-way ANOVA followed by Dunnett’s multiple comparison test.
